# Zno nanoparticles: improving photosynthesis, shoot development, and phyllosphere microbiome composition in tea plants

**DOI:** 10.1186/s12951-024-02667-2

**Published:** 2024-07-02

**Authors:** Hao Chen, Yujie Song, Yu Wang, Huan Wang, Zhaotang Ding, Kai Fan

**Affiliations:** 1 College of Horticulture, Qingdao Agricultural University, Qingdao, 266109 China; 2 Tea Research Institute, Shandong Academy of Agricultural Sciences, Jinan, 250100 China

**Keywords:** *Camellia sinensis* (L.) O. Kuntze, ZnO NPs, Photosynthetic capacity, Sprouting of new shoots, Epiphytic microorganisms, Endophytic microorganisms, Phyllosphere

## Abstract

**Background:**

Nanotechnology holds revolutionary potential in the field of agriculture, with zinc oxide nanoparticles (ZnO NPs) demonstrating advantages in promoting crop growth. Enhanced photosynthetic efficiency is closely linked to improved vigor and superior quality in tea plants, complemented by the beneficial role of phyllosphere microorganisms in maintaining plant health. However, the effects of ZnO NPs on the photosynthesis of tea plants, the sprouting of new shoots, and the community of phyllosphere microorganisms have not been fully investigated.

**Results:**

This study investigated the photosynthetic physiological parameters of tea plants under the influence of ZnO NPs, the content of key photosynthetic enzymes such as RubisCO, chlorophyll content, chlorophyll fluorescence parameters, transcriptomic and extensive targeted metabolomic profiles of leaves and new shoots, mineral element composition in these tissues, and the epiphytic and endophytic microbial communities within the phyllosphere. The results indicated that ZnO NPs could enhance the photosynthesis of tea plants, upregulate the expression of some genes related to photosynthesis, increase the accumulation of photosynthetic products, promote the development of new shoots, and alter the content of various mineral elements in the leaves and new shoots of tea plants. Furthermore, the application of ZnO NPs was observed to favorably influence the microbial community structure within the phyllosphere of tea plants. This shift in microbial community dynamics suggests a potential for ZnO NPs to contribute to plant health and productivity by modulating the phyllosphere microbiome.

**Conclusion:**

This study demonstrates that ZnO NPs have a positive impact on the photosynthesis of tea plants, the sprouting of new shoots, and the community of phyllosphere microorganisms, which can improve the growth condition of tea plants. These findings provide new scientific evidence for the application of ZnO NPs in sustainable agricultural development and contribute to advancing research in nanobiotechnology aimed at enhancing crop yield and quality.

**Graphical Abstract:**

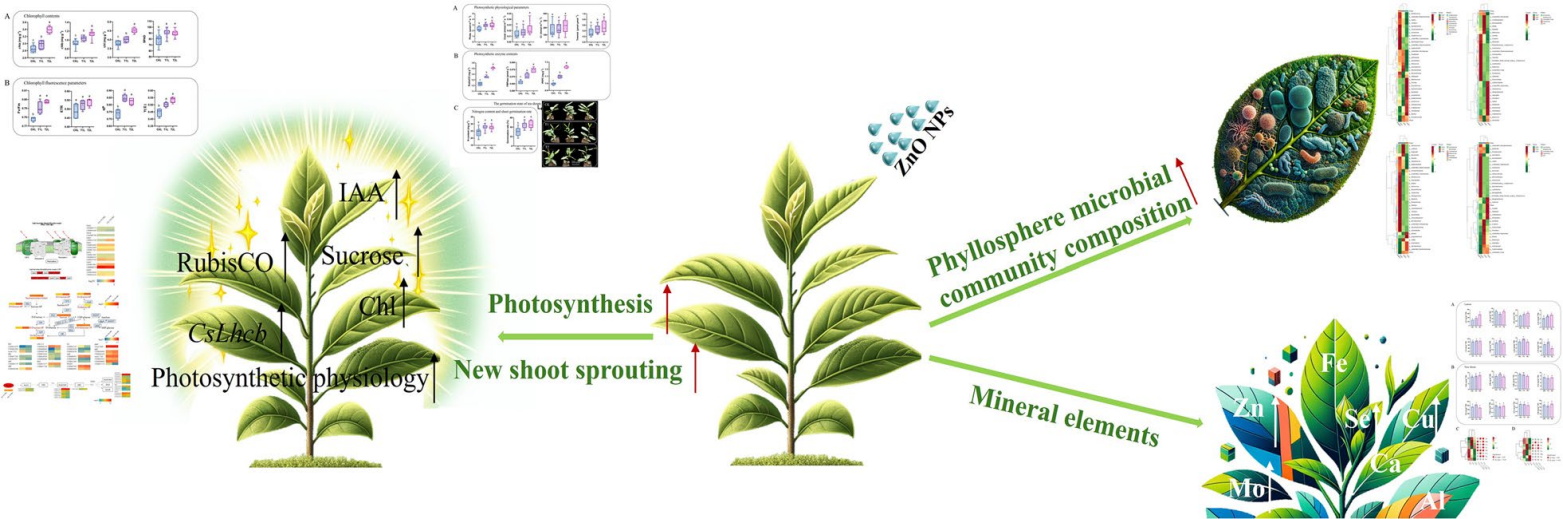

**Supplementary Information:**

The online version contains supplementary material available at 10.1186/s12951-024-02667-2.

## Background

Nanotechnology is emerging as a key innovator in agriculture, characterized by the unique properties of nanomaterials that include improved solubility, stability, and bioavailability [1, 2]. These attributes are pivotal when developing products for enhancing crop growth, managing pests and diseases, and mitigating environmental pollution [3–5].

Photosynthesis is the foundation of growth, development, and yield formation in tea plants. It is not only the key process to produce biomass and energy storage in tea plants but also directly affects the quality and yield of tea leaves [6]. The importance of photosynthesis for tea plants is also reflected in its impact on the synthesis of secondary metabolites. For example, the strength of photosynthetic capacity directly affects the synthesis of bioactive substances such as catechins in tea leaves, which are key factors determining the quality of tea [7]. At the same time, the sprouting of tea buds plays an important role in the life cycle of tea plants. From a biological perspective, the budding process is the cornerstone of plant growth, overall development, and reproduction [8]. From an economic perspective, tea buds are the main source of the economic value of tea plants and the raw material for producing premium finished tea. The earlier the tea buds sprout, the greater the economic value they can generate to some extent. Therefore, regulating the better sprouting of tea buds is our goal [9]. Thus, improving the efficiency of photosynthesis and promoting the sprouting of new shoots are crucial for tea production.

The positive effects of ZnO NPs on plants have been confirmed in multiple studies [10]. For instance, ZnO NPs have been shown to increase the growth rate and biomass of crops [11], enhance the efficiency of photosynthesis in plants [10], and improve the antioxidant system of plants [12]. These positive effects not only improve the overall health of plants but may also increase crop yield by enhancing the absorption and utilization rate of nutrients [13]. At the same time, ZnO NPs have been found to increase the zinc content and quality of wheat grains, enhance the aroma and nutritional components of rice, and strengthen rice resistance to blast disease [14–16]. Zinc plays multiple biological roles within plants, being a component of many enzymes and involved in nitrogen metabolism, protein synthesis, plant hormone regulation, and antioxidative defense [17]. Zinc is essential for plant growth and development. ZnO NPs serve as an innovative zinc source, offering plants the required nutrient and enhancing its utilization efficiency via nanoscale properties [18]. However, the impact of ZnO NPs on tea plants, especially in relation to photosynthesis, remains unknown.

As an emerging biological stimulant, ZnO NPs play a role by promoting plant growth, improving nutrient absorption efficiency, and enhancing plant resistance to environmental stress [19, 20]. Although current studies have shown the potential of ZnO NPs as biostimulants, their availability on the market may vary from region to region and is influenced by regulatory policies and safety assessments [21].

In the meantime, ZnO NPs are not the sole players in this domain; carbon dots (CDs) have also been recognized for their ability to augment plant photosynthesis, CDs offer unique advantages such as modifiable surface properties and low toxicity, making them suitable for agricultural applications [22]. The exploration of nanomaterials extends to a variety of other nanoparticles, including metallic nanoparticles like gold and silver, which have been shown to influence photosynthetic mechanisms and plant productivity [23, 24].

Phyllosphere microorganisms, including both epiphytic and endophytic species, are vital for plant health and productivity, influencing growth through nitrogen fixation, phosphate solubilization, hormone synthesis, and pathogen resistance [25–27]. These microbes also modulate photosynthesis and light energy utilization [28]. Despite their significance, phyllosphere microorganisms in tea plants are understudied in respect to soil microbiota [29].

Phyllosphere microorganisms (especially epiphytes) are highly susceptible to external environmental influences. Environmental factors such as climate conditions, atmospheric pollution, soil quality, and human agricultural activities can have direct or indirect effects on phyllosphere microorganisms [30, 31]. Chemicals used in agricultural activities can affect the diversity and activity of microbial communities by altering the chemical environment of the plant leaf surface [30]. The application of nanomaterials is considered an effective means to regulate the composition of phyllosphere microbial communities, as evidenced by recent studies exploring the impact of nanofertilizers on soil and plant-associated microbial communities [32]. These advances suggest that nanotechnology can play a role in shaping the microbial milieu of the phyllosphere, potentially influencing plant health and productivity. Studies have found that nanomaterials can affect plant growth by altering the community structure and function of phyllosphere microorganisms [33]. However, the impact of ZnO NPs on the microbial community of the tea plant phyllosphere remains unknown.

This article explores the effects of ZnO NPs on tea plant photosynthesis, sprouting of new shoots, mineral element content, and phyllosphere microbial communities (including both epiphytic and endophytic microorganisms). We hypothesize that ZnO NPs will have a positive impact on the growth of tea plants. At the same time, due to the strong bactericidal properties of ZnO NPs, we speculate that they will change the composition of the tea plant phyllosphere microbial community, potentially eliminating some potential plant pathogens residing in the phyllosphere. This study helps to understand the behavior and effects of ZnO NPs within plants, and through this research, we aim to provide a more in-depth and comprehensive scientific basis for the application of nanomaterials in sustainable agricultural development.

## Materials and methods

### Plant materials

In this study, one-year-old *Camellia sinensis* cv. Shuchazao tea plants were selected as the research subjects. The tea plants were planted in a nutrient-rich nursery substrate with an organic matter content of about 60%, a total porosity of about 75%, a bulk density of about 0.35%, and a pH of about 5.5. During the experiment, consistent irrigation conditions were maintained to keep the soil moisture content around 75%. Soil moisture was measured using a soil moisture meter (LD-G-300, Dongwan City Lide Technology Co., Ltd., Dongwan, China). The nursery substrate was provided by Shouguang Yixiandu Agricultural Science and Technology Co., Ltd. (Heze, China). The tea plants were grown under a photoperiod of 14 h of light and 10 h of darkness, with a daytime temperature of 28 °C, a nighttime temperature of 22 °C, light intensity of 10000 lx, and air humidity of 75%.

### Experimental design

Referring to the application of ZnO NPs on plants, three concentrations were selected for foliar spraying on tea plants: 0 mg L^−1^ (CK), 50 mg L^−1^ (T1), and 100 mg L^−1^ (T2) [15, 34, 35]. A total of 432 tea plants were planted, with 144 plants for each treatment, divided into 8 groups, with 18 plants per group. The zinc oxide nanoparticles used in this experiment had a particle size of 30 nm and a purity of more than 99.9%, provided by Shanghai Macklin Biochemical Co., Ltd. (Shanghai, China). Seven days after planting the tea plants, foliar spraying treatments were conducted every three days, for a total of three times. In this study, the first mature leaf below the new shoot was selected for research.

### Measurement of photosynthetic physiological parameters, photosynthetic enzyme content, nitrogen content, and sprouting rate of new shoots

The photosynthetic physiological parameters of tea plants (net photosynthetic rate (Photo), stomatal conductance (Cond), intercellular CO_2_ concentration (Ci), and transpiration rate (Trmmol)) were measured using a portable photosynthesis system (LI-6400XT, LI-COR, Inc. Lincoln, NE, USA). The measurements were taken at 9:00 AM, with an air flow rate set to 500 μmol m^−2^ s^−1^, a CO_2_ concentration of 400 μmol m^−2^ s^−1^, and light intensity consistent with the growth conditions of the tea plants. Twelve plants were randomly selected from each treatment for measurement.

The contents of ribulose-1,5-bisphosphate carboxylase/oxygenase (RubisCO), fructose-1,6-bisphosphatase (FBP), and phosphoenolpyruvate carboxylase (PEPC) were measured by Genepioneer Biotechnologies (Nanjing, China) using research reagent kits (ELISA method), provided by Jiangsu Jingmei Biotechnology Co., Ltd. (Yancheng, China) (product numbers A-P0018B, A-P0357B, and A-P0104B), with ten replicates for each treatment.

Leaf nitrogen content was measured using a plant nutrition analyzer (TYS-4H, Top Cloud-Agri, Zhejiang, China), with sixteen replicates for each treatment.

The sprouting rate of new shoots was determined by counting the number of sprouted shoots in each of the eight groups of tea plants (each group consisting of 18 plants) per treatment, with a shoot considered sprouted once it was fully expanded.

### Measurement of chlorophyll content and chlorophyll fluorescence parameters

Chlorophyll content was measured using a research reagent kit provided by Suzhou Grace Biotechnology Co., Ltd. (Suzhou, China) (product number G0601W), with ten replicates for each treatment. The soil plant analysis development (SPAD) value was measured using a plant nutrition analyzer (TYS-4H, Top Cloud-Agri, Zhejiang, China), with sixteen replicates for each treatment.

Chlorophyll fluorescence parameters were measured using a chlorophyll fluorescence imaging system (IMAGING-PAM, WALZ, Effeltrich, Germany). Leaves were dark-adapted for 30 min, and the instrument was calibrated for light intensity and Abs. The actinic light was set to 5, with other parameters set to the default values of the program. Four plants were randomly selected from each treatment for measurement.

### Measurement of mineral elements in leaves and new shoots

The content of mineral elements in leaves and new shoots was determined by atomic absorption spectrophotometry, with three biological replicates per treatment. Fresh tea plant samples were processed by weighing 1.0 g into a digestion vessel, followed by the addition of nitric acid and an overnight soaking. A stepwise heating protocol was applied, culminating at 160 °C, before cooling to room temperature. The digested sample was then transferred to a volumetric flask, rinsed, and diluted to volume with nitric acid for elemental analysis. A reagent blank was prepared in parallel. Element concentrations were quantified using an inductively coupled plasma emission spectrometer. The content of elements in the sample (mg kg^−1^) = c1V1 m^−1^, where c1 is the concentration of the element measured by the instrument, V1 is the volume of digestion (mL), and m is the mass of the sample (g).

### Transcriptome measurement of leaves and new shoots

The transcriptome sequencing process includes total RNA extraction, mRNA enrichment, double-stranded cDNA synthesis, end repair, A-tailing and adapter ligation, fragment selection and PCR amplification, library quality assessment, and Illumina sequencing (See Additional file 1 for specific steps). Three biological replicates were measured for each treatment (For the transcriptome analysis, the first mature leaf at the same leaf position under the new shoot of each tea plant was selected as the subject of study, with only one leaf being taken from each tea plant. From this selection, three leaves constituted a single sample, and each of these samples was precisely weighed to 0.1 g to ensure uniformity in the genetic material available for subsequent transcriptome determination. For the new shoots of the tea plant, three shoots from three tea plants constituted one sample, and each sample was also weighed to 0.1 g for transcriptome sequencing).

To ensure high-quality RNA and the absence of DNA contamination, the extracted RNA was rigorously analyzed for integrity and precisely tested for purity and concentration. The research process began with RNA samples prepared using the NEBNext Ultra RNA Library Prep Kit (New England Biolabs, Ipswich, MA, USA) for Illumina. Purified fragments underwent PCR, and their quality was evaluated using the Agilent Bioanalyzer 2100 system. Index-coded samples were clustered on a cBot Cluster Generation System and sequenced to generate 150 bp paired-end reads. The data was filtered using fastp to produce 'clean reads'. Clean reads were compared to the reference genome using HISAT. Gene prediction was performed with StringTie, which provided faster and more accurate transcript splicing. FeatureCounts assisted in gene alignment, and the FPKM of each gene was calculated based on their specific length.

### Extensive targeted metabolome measurement of leaves and new shoots

Biological samples (For leaves, the first mature leaf under the same leaf position below the shoot was selected, with only one leaf being taken from each tea plant, and the three leaves formed a sample, of which 0.5 g was weighed for determination from each sample. For the new shoots, five shoots from five tea plants constituted one sample, and each sample was also weighed to 0.5 g for extensive targeted metabolomics analysis) were processed using vacuum freeze-drying technology in a lyophilizer (Scientz-100F), followed by grinding at 30 Hz for 1.5 min using a grinder (MM 400, Retsch). 50 mg of the resulting sample powder was weighed with an electronic balance (MS105DΜ) and mixed with 1.2 mL of pre-cooled 70% methanolic aqueous solution containing an internal standard. The mixture was subjected to intermittent vortexing and centrifugation. After centrifugation, the supernatant was separated, filtered, and prepared for UPLC-MS/MS analysis.

Under UPLC conditions, an UPLC-ESI–MS/MS system (UPLC, ExionLC™ AD, https://sciex.com.cn/) and a tandem mass spectrometry system (https://sciex.com.cn/) were used to analyze the sample extracts. A precise mobile phase gradient was employed using solvent A (pure water with 0.1% formic acid) and solvent B (acetonitrile with 0.1% formic acid). Precise flow rate, temperature, and injection volumes were maintained throughout the process.

The operating parameters of the ESI-Q TRAP-MS/MS included preset values for source temperature, ion spray voltage, gas pressures, and collision-activated dissociation levels. The QQQ scans were performed as MRM experiments, with DP and CE adjusted for individual MRM transitions. Specific MRM transitions were closely monitored throughout the experiment, aimed at identifying the metabolites eluted during the period.

### Measurement of epiphytic and endophytic microorganisms in the phyllosphere

This study utilized 16S rDNA amplicon sequencing to measure epiphytic and endophytic bacteria and Internal Transcribed Spacer (ITS) sequencing for epiphytic and endophytic fungi [36].

#### Extraction of epiphytic microorganisms

For each sample, 15 g of tea leaves (approximately 50 leaves) were placed in a sterile tube. In the growing environment of tea plants, sterile gloves were used to remove tea leaves from the stem and quickly put them into sterile tubes, to which 150 mL of potassium phosphate buffer (0.1 mol L^−1^, pH = 8) were added. The samples were ultrasonicated for 1 min, vortexed for 10 s, these steps were repeated twice. After washing, the leaves were once again immersed in the aforementioned potassium phosphate buffer solution, followed by another round of ultrasonic washing and vortexing [37]. The washing liquid was filtered through a 0.22 μm filter membrane, which was then flash-frozen in liquid nitrogen and stored at − 80 °C.

#### Extraction of endophytic microorganisms

The leaf surfaces were successively soaked in 75% ethanol for 1 min, 3.25% sodium hypochlorite for 3 min, and 75% ethanol for 30 s, followed by rinsing three times with sterile distilled water. The leaves were then freeze-dried in liquid nitrogen [38].

#### Extraction of genomic DNA and PCR amplification

Genomic DNA was extracted from filters (for epiphytic microorganisms) or samples (for endophytic microorganisms) using the CTAB method [39]. The diluted genomic DNA served as a template for PCR, which was carried out using barcode-specific primers tailored to target various rRNA microbial gene regions. Primers for 16S V4 region (515F and 806R) were used to identify bacterial diversity. ITS1 region primers (ITS5-1737F and ITS2-2043R) were used to identify fungal diversity.. The PCR reactions utilized Phusion® High-Fidelity PCR Master Mix with GC Buffer (New England Biolabs), ensuring high amplification efficiency and accuracy. The reaction mixture consisted of 15 µL of the master mix, 2 µM of each forward and reverse primer, and approximately 10 ng of template DNA. The thermal cycling protocol involved an initial denaturation at 98 °C for 1 min, followed by 30 cycles of denaturation at 98 °C for 10 s, annealing at 50 °C for 30 s, and elongation at 72 °C for 30 s, concluding with a final extension at 72 °C for 5 min.

#### Mixing and purification of PCR products

The PCR products were evaluated by electrophoresis on a 2% agarose gel. Qualified PCR products were then purified using SPRIselect magnetic beads (Beckman Coulter, Brea, CA, USA) of size range 1–3 µm, enzymatically quantified, and pooled in equimolar ratios based on the concentration of the PCR products. Following thorough mixing, the pooled PCR products were subjected to a second electrophoresis on a 2% agarose gel. Target bands were subsequently excised and extracted using the QIAquick Gel Extraction Kit (Qiagen, Hilden, Germany).

#### Library construction and sequencing

Libraries were constructed using the TruSeq^®^ DNA PCR-Free Sample Preparation Kit (Illumina, San Diego, CA, USA), quantified by Qubit and Q-PCR, and sequenced on a NovaSeq6000 (Illumina, Inc., San Diego, CA, USA) after passing quality control.

#### Sequencing data processing

Sample data were demultiplexed from the raw data based on barcode sequences and PCR primer sequences, and the barcode and primer sequences were trimmed. High-quality reads were obtained by filtering the raw reads using fastp (v0.22.0, https://github.com/OpenGene/fastp).

### Quantitative real-time PCR validation

Reverse transcription amplification was performed using the SynScript^®^ III RT SuperMix for qPCR reverse transcription kit produced by Beijing Biotech Co., Ltd. (Beijing, China). The resulting cDNA products were diluted fourfold and used as templates for qPCR amplification with Tsingke ArtiCanCEO SYBR qPCR Mix. CsGAPDH was used as the reference gene, and gene expression was calculated using the 2^−ΔΔCT^ method. The qRT-PCR samples underwent three biological replicates and three technical replicates. All primers used in this study are listed in Table S1.

### Statistical and bioinformatics analyses

#### Bioinformatics analyses

Our data analysis employed several bioinformatics tools within the R environment and complementary software. Overlap among gene sets was visualized using Venn diagrams, created with the 'VennDiagram' package (v1.6.20) in R (v3.5.1). Functional enrichment analyses, encompassing both Gene Ontology (GO) and Kyoto Encyclopedia of Genes and Genomes (KEGG) pathway analyses, were executed using 'clusterProfiler' (v3.10.1) and 'ggplot2' (v3.3.0) packages in R (v3.5.1), respectively. Modules of highly correlated genes were identified through Weighted Gene Co-expression Network Analysis (WGCNA) using the 'WGCNA' package (v1.69) in R (v3.5.1). For integrative multi-omics data analysis, Orthogonal Partial Least Squares Discriminant Analysis (O2PLS) was performed with the 'OmicsPLS' package (v2.0.2) in R (v4.2.0).

#### Microbial community analysis

Species annotation for Amplicon Sequence Variants (ASVs) was conducted using Mothur (v1.48), referencing the SSUrRNA database from SILVA (v138.1). ASV analysis, providing higher taxonomic resolution than traditional OTU clustering, was performed, and sequences were aligned using MAFFT (v7.520). Data normalization across samples was achieved by equalizing to the minimum dataset size. Alpha diversity and Non-metric Multidimensional Scaling (NMDS) analyses were conducted using 'phyloseq' (v1.40.0) and 'vegan' (v2.6.2) packages in R (v4.2.0). Variance within and between microbial communities was assessed through Analysis of Molecular Variance (AMOVA) using the 'amova' function in Mothur. Biomarkers were determined using Linear Discriminant Analysis Effect Size (LEfSe) analysis (v1.1.2), with an LDA score threshold of 4.

#### Microbial community functional analysis

Functional capabilities of microbial communities were predicted with Tax4Fun2 (v1.1.3), correlating SILVA taxonomic identifiers with reference genomes. Ecological functions for prokaryotic taxa were annotated via the FAPROTAX tool (v1.2.4). Fungal taxa were categorized into functional guilds using FunGuild (v1.1), leveraging literature and database resources.

#### Correlation analyses

Significant features and correlations were identified using Random Forest analysis with the 'varSelRF' package (v0.7.8) and Pearson correlation analysis with the 'stats' package (v3.5.1) in R (v3.5.1).

#### General statistical analysis

All measured parameters underwent descriptive statistical analysis to compute means and standard deviations. Differences among treatment groups were tested using one-way ANOVA, followed by Tukey's post-hoc test for pairwise comparisons. A p-value < 0.05 was considered statistically significant. These analyses were performed using SPSS (v25.0, IBM Corp.). Data visualization was accomplished with GraphPad Prism (v8.0, GraphPad Software, Inc.).

## Results

### Effects of ZnO NPs on vegetative growth parameters in tea plants

**Fig. 1:**
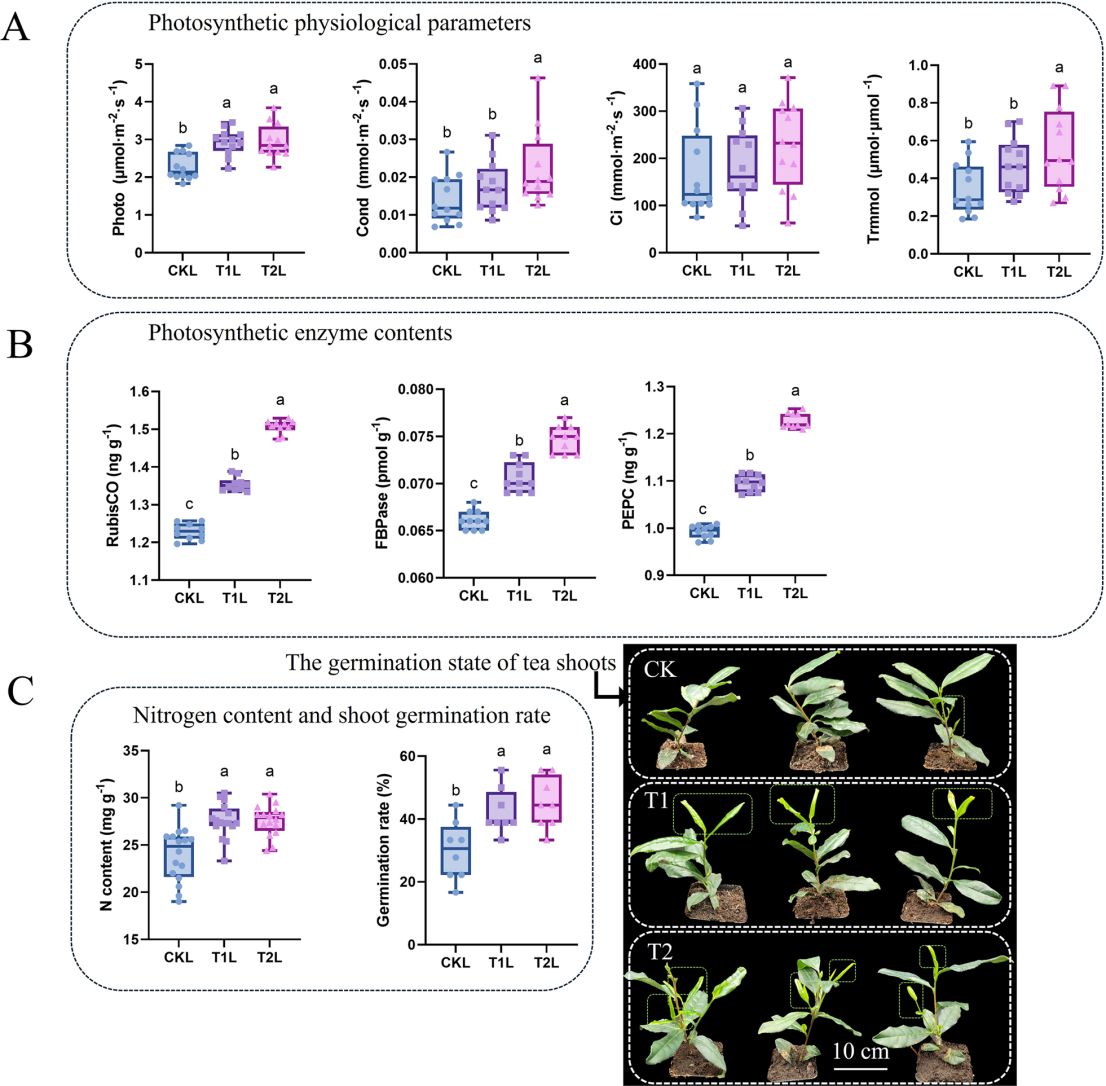
Effects of ZnO NPs on photosynthetic physiological parameters (**A**), photosynthetic enzyme content (**B**), nitrogen content, sprouting rate, and sprouting status of new shoots (**C**) in tea plants. Data were analyzed using one-way ANOVA, and post hoc comparisons were conducted using Tukey’s HSD test. The different letters indicate significantly different values (p < 0.05) according to the results of the Tukey’s test

To investigate the effects of ZnO NPs on photosynthesis and new shoot development in tea plants, we measured the Photo, Cond, Ci, Trmmol, RubisCO content, FBP content, PEPC content, nitrogen content, and sprouting rate of new shoots. As shown in Fig. 1, except for Ci, ZnO NPs significantly increased the Photo, Cond, and Trmmol of tea plants, with the most significant effects observed at a concentration of 100 mg L^−1^ (Fig. 1A). Simultaneously, ZnO NPs significantly increased the content of photosynthetic enzymes in the leaves (Fig. 1B). Additionally, the nitrogen content in tea leaves also increased under the influence of ZnO NPs (Fig. 1C). Interestingly, ZnO NPs also improved the sprouting rate of new shoots, although the two different concentrations did not show significant differences in their effects on the sprouting rate (Fig. 1D). In summary, ZnO NPs significantly enhanced the photosynthetic capacity of tea leaves and the sprouting rate of new shoots.

### Effects of ZnO NPs on chlorophyll content and chlorophyll fluorescence parameters in tea plants

**Fig. 2:**
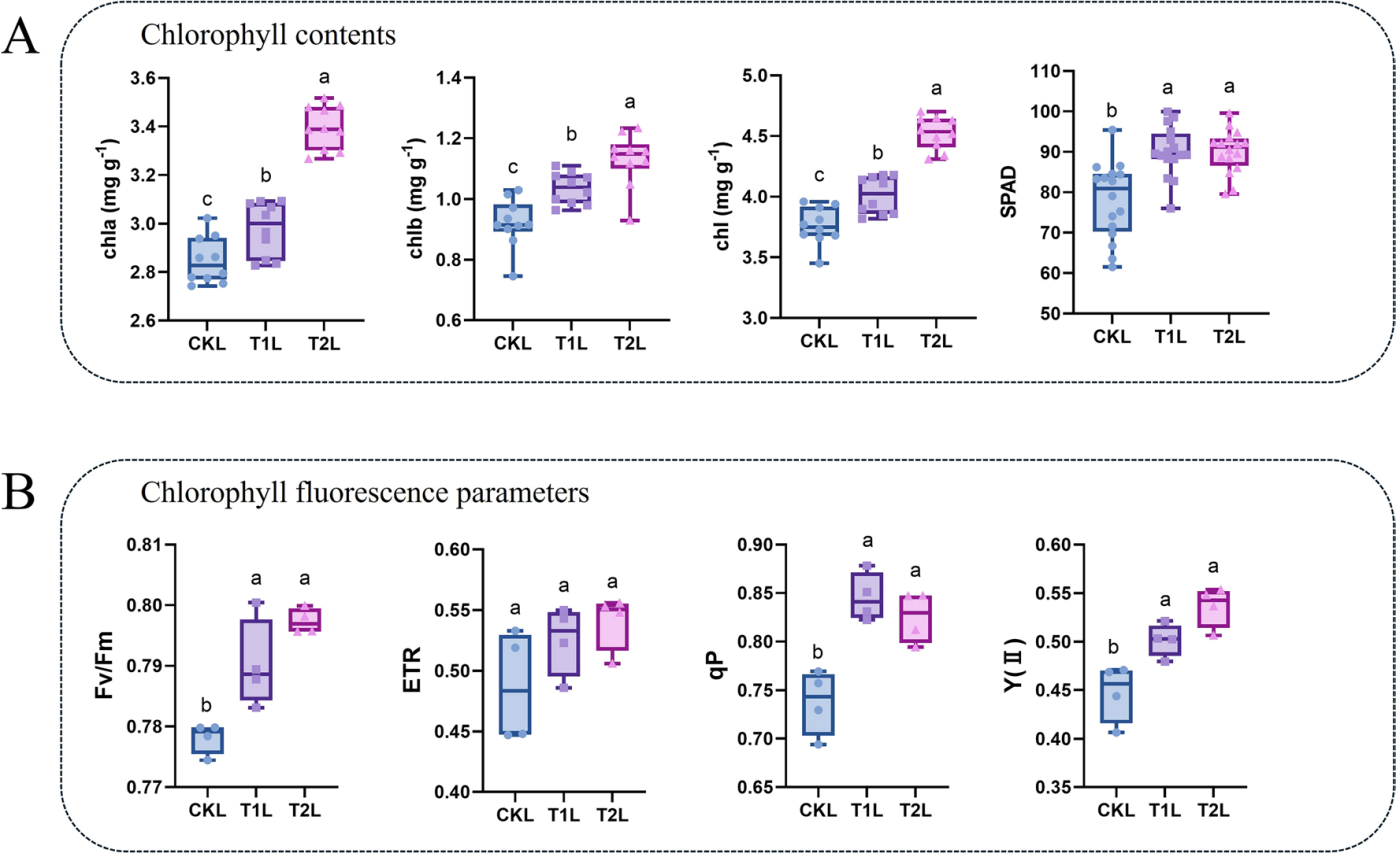
Effects of ZnO NPs on chlorophyll content (**A**) and chlorophyll fluorescence parameters (**B**) in tea plants. Data were analyzed using one-way ANOVA, and post hoc comparisons were conducted using Tukey's HSD test. The different letters indicate significantly different values (p < 0.05) according to the results of the Tukey’s test

We investigated the effects of ZnO NPs on chlorophyll levels and fluorescence characteristics in tea plants by assessing leaf chlorophyll content, SPAD values, and various chlorophyll fluorescence parameters (Fig. 2). The combined measurements of total chlorophyll, chlorophyll a (chl a), chlorophyll b (chl b), and SPAD values indicate that ZnO NPs can increase the chlorophyll content in tea leaves (Fig. 2A). In terms of chlorophyll fluorescence parameters, aside from the relative electron transport rate (ETR), ZnO NPs significantly enhanced the maximum photochemical efficiency of photosystem II (Fv/Fm), the photochemical quenching coefficient (qP), and the actual photochemical efficiency of photosystem II (Y(II)) (Fig. 2B). In summary, both the chlorophyll content and chlorophyll fluorescence parameters demonstrate that ZnO NPs can significantly improve the photosynthetic capacity of tea leaves.

### Effects of ZnO NPs on the content of mineral elements in tea leaves and new shoots

**Fig. 3:**
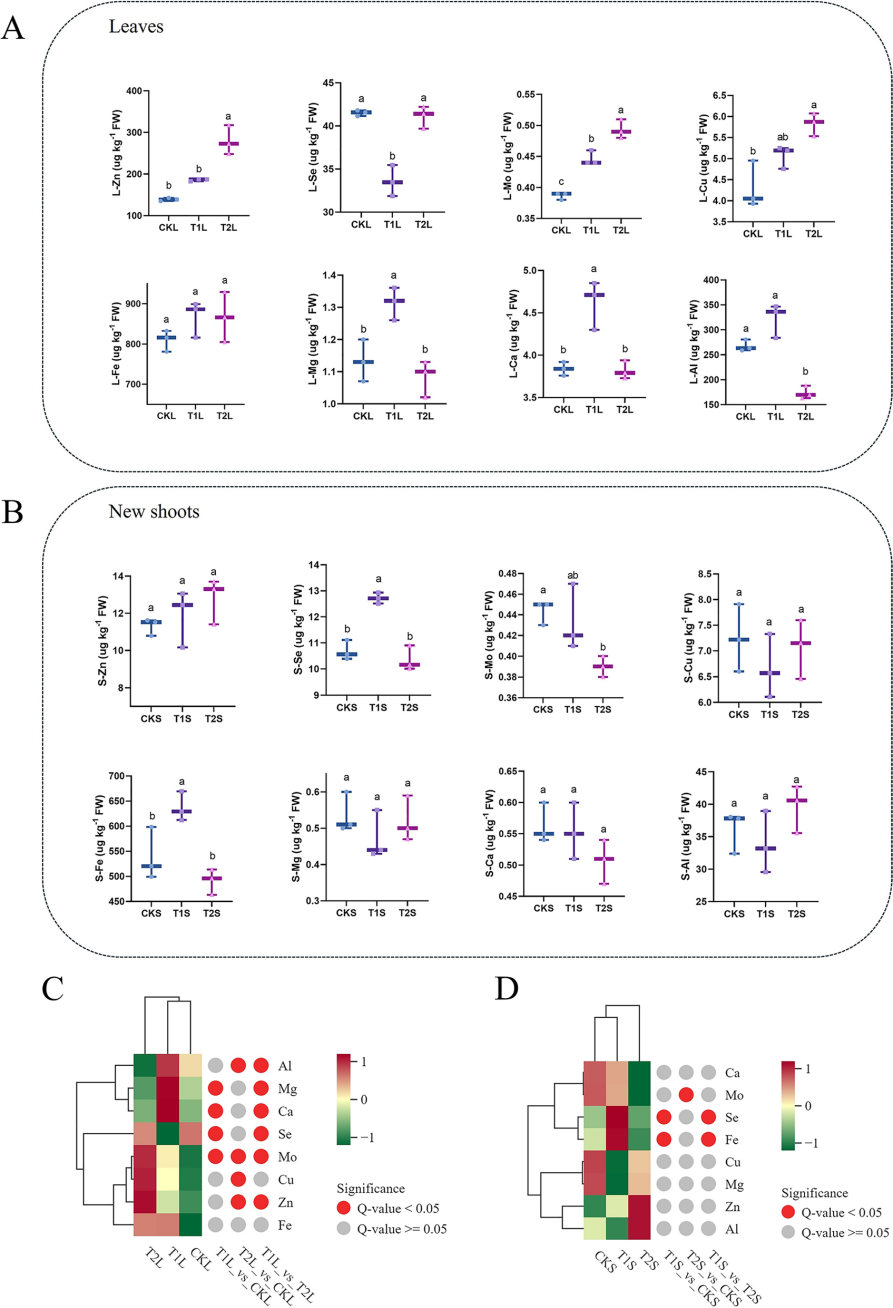
Effects of ZnO NPs on the content of mineral elements in tea leaves (**A**) and new shoots (**B**). Data were analyzed using one-way ANOVA, and different letters show significantly different values (p < 0.05). “FW” stands for fresh weight. The content of elements in tea leaves (**C**) and new shoots (**D**) under the influence of ZnO NPs is normalized at the sample level, reflecting the trends and similarities in substance variation across different samples. Q-value Multiple hypothesis test corrected p value

To explore the impact of applying one mineral element (ZnO NPs) on other minerals in tea leaves and new shoots, we measured the content of eight mineral elements related to the growth and quality of tea plants (Fig. 3). Under the influence of ZnO NPs, the zinc content in tea leaves increased, but a significant difference was only observed at the concentration of 100 mg L^−1^ (T2) (Fig. 3A). Selenium showed significant differences under the influence of ZnO NPs; at the concentration of 50 mg L^−1^ (T1), the selenium content in the leaves significantly decreased, while it significantly increased in the new shoots, suggesting that at this concentration, ZnO NPs promoted the transfer of selenium from the leaves to the new shoots. This difference was not observed at T2 (Fig. 3A, B). Meanwhile, as the concentration of ZnO NPs increased, the molybdenum content in the leaves significantly increased, but the molybdenum content in the new shoots significantly decreased at T2 (Fig. 3A, B). The copper content in the leaves significantly increased under T2, but no significant difference was observed in the copper content in the new shoots. At the same time, no significant changes were found in the iron content in the leaves, but the iron content in the new shoots significantly increased at T1 (Fig. 3A, B). The magnesium content in the leaves significantly increased at T1, with no significant differences observed in the new shoots. Similar to magnesium, the calcium content in both leaves and new shoots showed a consistent expression trend, with a significant increase in calcium content in the leaves only at T1. Analogous to magnesium, the calcium levels in the leaves and new shoots displayed a uniform trend, with a notable elevation in leaf calcium content observed exclusively at T1 (Fig. 3A, B).Unlike the other mineral elements, the aluminum content in the leaves significantly decreased at T2, with no significant differences observed in the new shoots (Fig. 3A, B). Cluster analysis results indicate that the use of ZnO NPs has a significant impact on the mineral element content in tea plant leaves, while the effect on the mineral content in new shoots is relatively minor (Fig. 3C, D). In summary, ZnO NPs had a significant impact on the content of mineral elements in tea leaves, with a greater effect on the leaves than on the new shoots.

### Gene expression in tea plant leaves and new shoots under the influence of ZnO NPs

In this study, we conducted transcriptomic sequencing on tea plant leaves and buds treated with ZnO NPs. A total of 65.10 Gb Clean Data was obtained from 9 leaf samples, and 70.66 Gb Clean Data from 9 new shoot samples. The percentage of Q20 bases was above 97.63%, and the percentage of Q30 bases was above 93.57% for all 18 samples, indicating high transcriptome quality suitable for subsequent analysis (Table S2). Clean Reads were aligned with the reference genome shuchazao_V2_Genome.fas (downloadable at: http://tpia.teaplants.cn/download.html) to obtain positional information on the reference genome or genes and unique sequence characteristics of the sequencing samples.

To explore the impact of ZnO NPs on gene expression in tea plants, we screened for differentially expressed genes (DEGs) in leaves and new shoots. The selection criteria for DEGs in this experiment were |log2Fold Change|≥ 1 and FDR (False Discovery Rate) < 0.05. A total of 1771 DEGs were identified between T1L and CKL, with 976 upregulated and 804 downregulated; 2448 DEGs were identified between T2L and CKL, with 1308 upregulated and 1140 downregulated (Fig. S1A). Between T1S and CKS, 169 DEGs were identified, with 126 upregulated and 43 downregulated; between T2S and CKS, 88 DEGs were identified, with 69 upregulated and 19 downregulated (Fig. S1B). The Venn diagram also displays the number of DEGs that are either shared or unique to leaves (Fig. S2A) and new shoots (Fig. S2B) under three different concentrations. To explore the expression patterns of DEGs in tea plant leaves (Fig. S3A) and new shoots (Fig. S3B) after ZnO NPs treatment, we normalized the data using Z-score and clustered genes with similar or close expression patterns to infer the function of unknown genes or unknown functions of known genes. Furthermore, to study the expression patterns of genes under ZnO NPs treatment, we first normalized the FPKM of all DEGs using R language and then performed K-means clustering analysis. Genes in the same cluster have similar trends under ZnO NPs treatment and may have similar functions. We also conducted K-means clustering analysis, with leaf DEGs divided into 10 clusters (Fig. S4A) and new shoot DEGs into 9 clusters (Fig.S4B), indicating significant differences in gene expression in tea plant leaves and new shoots under different concentrations of treatment. The KEGG enrichment analysis, GO enrichment analysis, and qRT-PCR Analysis of differentially expressed genes in tea plant leaves and new shoots under the influence of ZnO NPs can be found in Additional file 2.

### Changes in metabolite composition and content in tea plant leaves and new shoots under the influence of ZnO NPs

In this study, we conducted a comprehensive targeted metabolomics analysis of tea plants treated with ZnO NPs using UPLC-MS/MS. A total of 2177 metabolites were detected in the leaves, and 2181 metabolites were detected in the new shoots. Principal component analysis (PCA) showed that in the leaves, PC1 accounted for 26.86% and PC2 accounted for 18.94% of the metabolite variance (Fig. S5A); in the new shoots, PC1 accounted for 20.79% and PC2 accounted for 15.64% (Fig. S5B). The criteria for selecting differential metabolites in this experiment were based on a Variable Importance in Projection (VIP) > 1 from the OPLS-DA model, along with a fold change ≥ 2 or fold change ≤ 0.5. Between T1L and CKL, 179 differential metabolites were detected, with 92 upregulated and 87 downregulated (Fig. S6A); between T2L and CKL, 175 differential metabolites were detected, with 99 upregulated and 76 downregulated (Fig. S6B). Between T1S and CKS, 83 differential metabolites were detected, with 38 upregulated and 45 downregulated (Fig. S6C); between T2S and CKS, 96 differential metabolites were detected, with 48 upregulated and 48 downregulated (Fig. S6D). Based on the trend of metabolite concentration changes after treatment with different concentrations of ZnO NPs, the metabolites in tea plant leaves were divided into 8 clusters (Fig. S7A); the metabolites in tea plant new shoots were divided into 7 clusters (Fig. S7B). The differential metabolites and KEGG enrichment analysis of differential metabolites in tea plant leaves and new shoots under the influence of ZnO NPs can also be found in Additional file 2.

### Integrated analysis of differential gene expression and differential metabolite expression reveals the intrinsic mechanism behind ZnO NPs enhancing photosynthetic capacity and promoting new shoot development in tea plants

#### photosynthesis-related pathways and endogenous auxin pathway metabolites and gene expression

**Fig. 4:**
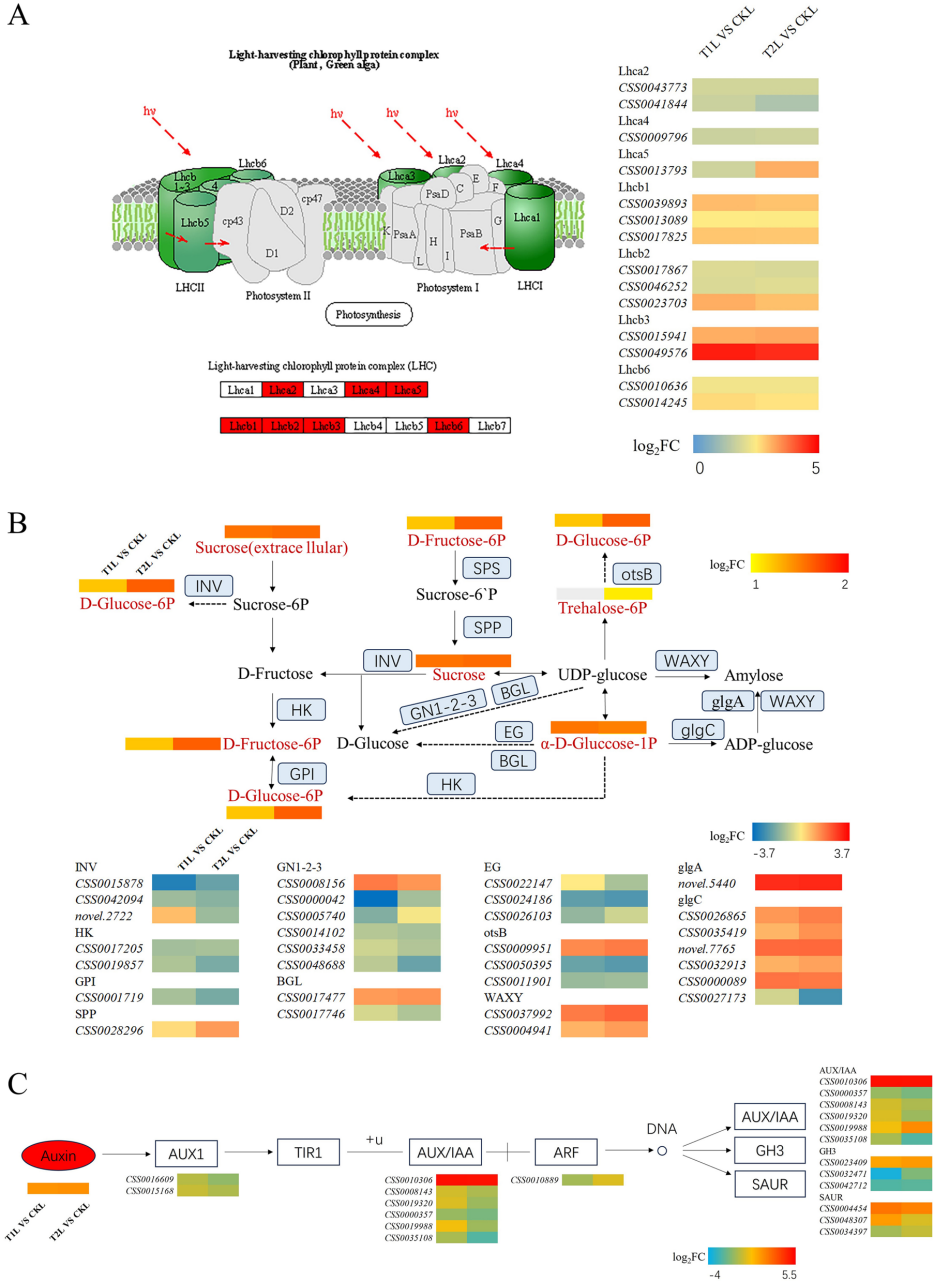
Expression levels of genes related to Photosynthesis—antenna proteins under the influence of ZnO NPs (**A**). Expression levels of genes and metabolites related to starch and sucrose metabolism under the influence of ZnO NPs (**B**). Expression levels of genes and metabolites related to the auxin synthesis pathway under the influence of ZnO NPs (**C**). The color scale ranges from blue to red, where red and blue boxes indicate that the expression of differential genes or the content of differential metabolites is higher or lower, respectively, compared to the untreated control group. A gray icon in the figure indicates that the substance was not detected in that treatment group

To comprehensively elucidate the response of tea plants to ZnO NPs from both gene expression and metabolite perspectives, we conducted an integrated analysis of the transcriptome and metabolome. Under the treatment of two concentrations of ZnO NPs, tea leaves were significantly enriched in pathways related to photosynthesis, specifically Photosynthesis—antenna proteins (Fig. 4A). In this pathway, all the differentially expressed genes compared to the control were significantly upregulated, mainly regulating the light-harvesting complex II chlorophyll a/b binding proteins (Lhca2, Lhca4, Lhca5, Lhcb1, Lhcb2, Lhcb3, and Lhcb6). Interestingly, in the Starch and sucrose metabolism pathway closely related to photosynthesis, all differential metabolites such as Sucrose, D-Glucose-6P, D-Fructose-6P, D-Glucose-6P, etc., showed a significant increase in content. We also presented the significant gene expression levels related to this pathway (Fig. 4B). Furthermore, under both concentrations of ZnO NPs treatment, the endogenous auxin content was significantly increased, and the gene and metabolite expression levels in the related metabolic pathway are shown in Fig. 4C.

#### Weighted gene co-expression network analysis (WGCNA) of differential genes and key differential metabolites (identification of coexpressed gene networks and key candidates)

**Fig. 5:**
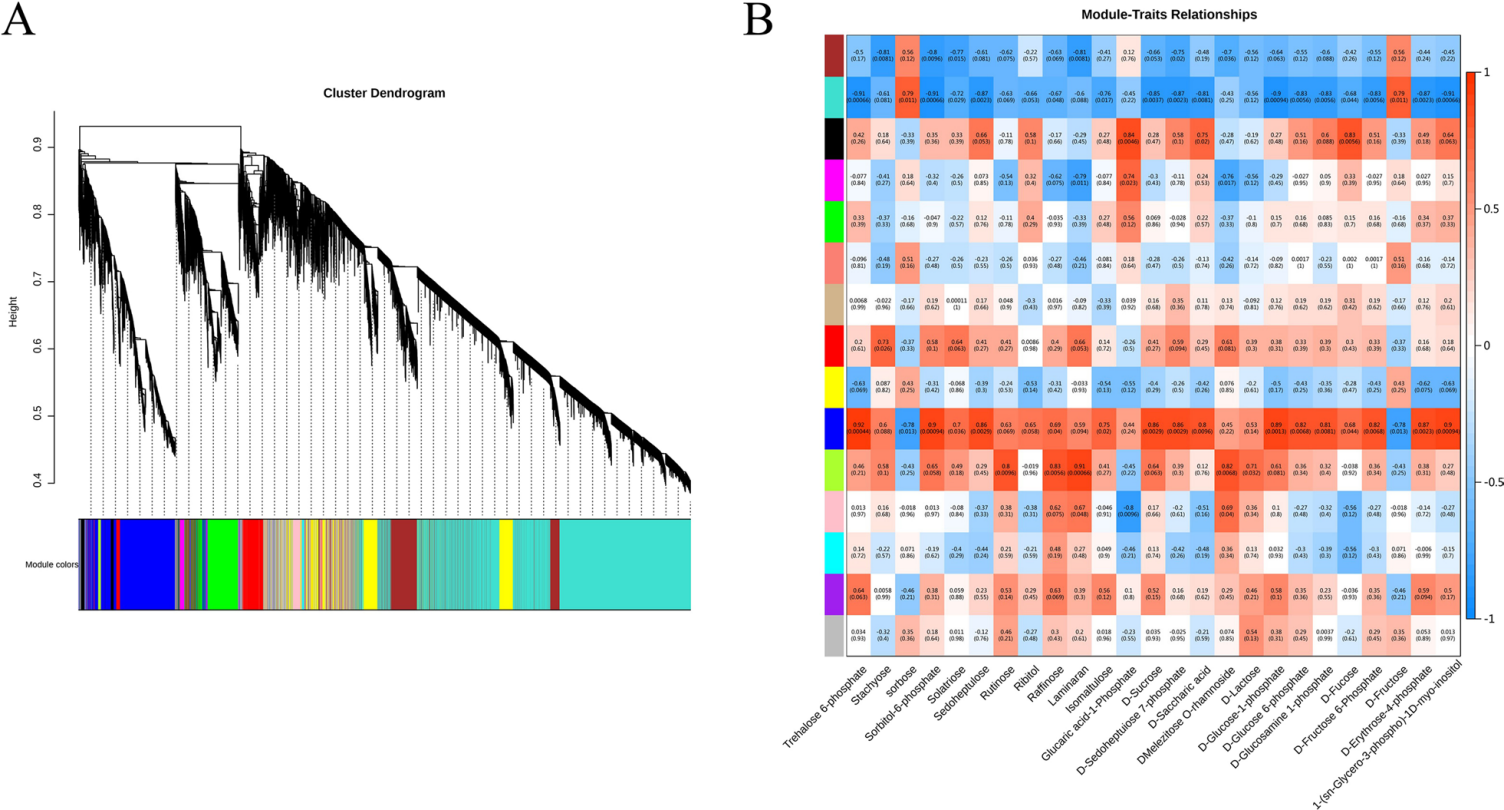
Hierarchical clustering of gene networks in tea leaves under the influence of ZnO NPs and key candidate genes with co-expressed genes in 14 modules. Each leaflet on the tree corresponds to an individual gene (**A**), with the horizontal axis representing metabolite names and the vertical axis with each color representing a module. The deeper the color (deep red or deep blue), the stronger the correlation between the metabolite and the module, and the lighter the color, the weaker the correlation between the sample and the module. Red indicates a positive correlation, while blue indicates a negative correlation (**B**)

To further understand the regulation of sucrose metabolism changes in tea leaves caused by ZnO NPs, WGCNA was conducted to study the co-expression network of differentially expressed genes (DEGs) (WGCNA is an algorithm for constructing gene co-expression networks, identifying modules of highly correlated genes from mRNA expression data to elucidate biological pathways.). Based on similar expression patterns, a total of 14 co-expressed modules were identified (Fig. 5). Each color in Fig. 5A represents that each gene on the cluster tree corresponding to a color belongs to the same module. If some genes always have similar expression changes in a physiological process or different tissues, then these genes may be functionally related. They can be defined as a module. For the upper half of the tree, the vertical distance represents the distance between two nodes (between genes), and the horizontal distance is meaningless. Genes preferentially expressed in sugar metabolism-related significant differential metabolites were mainly concentrated in the MEblue module, with 11 metabolites having a correlation R^2^ greater than 0.8 with this module. The key product of sugar metabolism, Sucrose, also had the highest correlation with this module (R^2^ = 0.86, p = 0.0027) (Fig. 5B). Within the MEblue module, 54 genes related to starch and sucrose metabolism were identified, including 11 regulating glucose-1-phosphate adenylyltransferase (*CSS0000089, CSS0026130, CSS0026865, CSS0032913, CSS0035261, CSS0035419, CSS0050226, novel.1469, novel.4393, novel.4625, novel.7765*); 2 regulating glucose-6-phosphate isomerase (*CSS0002435, CSS0045493*); 2 regulating trehalose 6-phosphate synthase/phosphatase (*CSS0002735, CSS0035721*); 5 regulating beta-glucosidase (*CSS0003115, CSS0006278, CSS0016696, CSS0017477, CSS0036292*); 6 regulating beta-amylase (*CSS0003801, CSS0018030, CSS0027311, CSS0032302, CSS0043627, CSS0047759*); 3 regulating granule-bound starch synthase (*CSS0004941, CSS0037992, CSS0045869*); 5 regulating starch synthase (*CSS0005914, CSS0021129, CSS0023626, CSS00248447, novel.5440*); 5 regulating glucan endo-1,3-beta-glucosidase 1/2/3 (*CSS0008156, CSS0012190, CSS0043010, CSS0048079, CSS0050142*); 1 regulating alpha,alpha-trehalase (*CSS0008297*); 1 regulating alpha-amylase (*CSS0008836*); 3 regulating trehalose 6-phosphate phosphatase (*CSS0009951, CSS0045703, novel.736*); 3 regulating glycogen phosphorylase (*CSS0015277, CSS0022613, CSS0038552*); 2 regulating 1,4-alpha-glucan branching enzyme (*CSS0016120, novel.877*); 1 regulating sucrose-phosphate synthase (*CSS0024623*); 1 regulating sucrose-6-phosphatase (*CSS0028296*); 2 regulating beta-fructofuranosidase (*CSS0033878, novel.2722*); 1 regulating ADP-sugar diphosphatase (*CSS0042147*).

#### Two-way orthogonal partial least squares (O2PLS) analysis

**Fig. 6:**
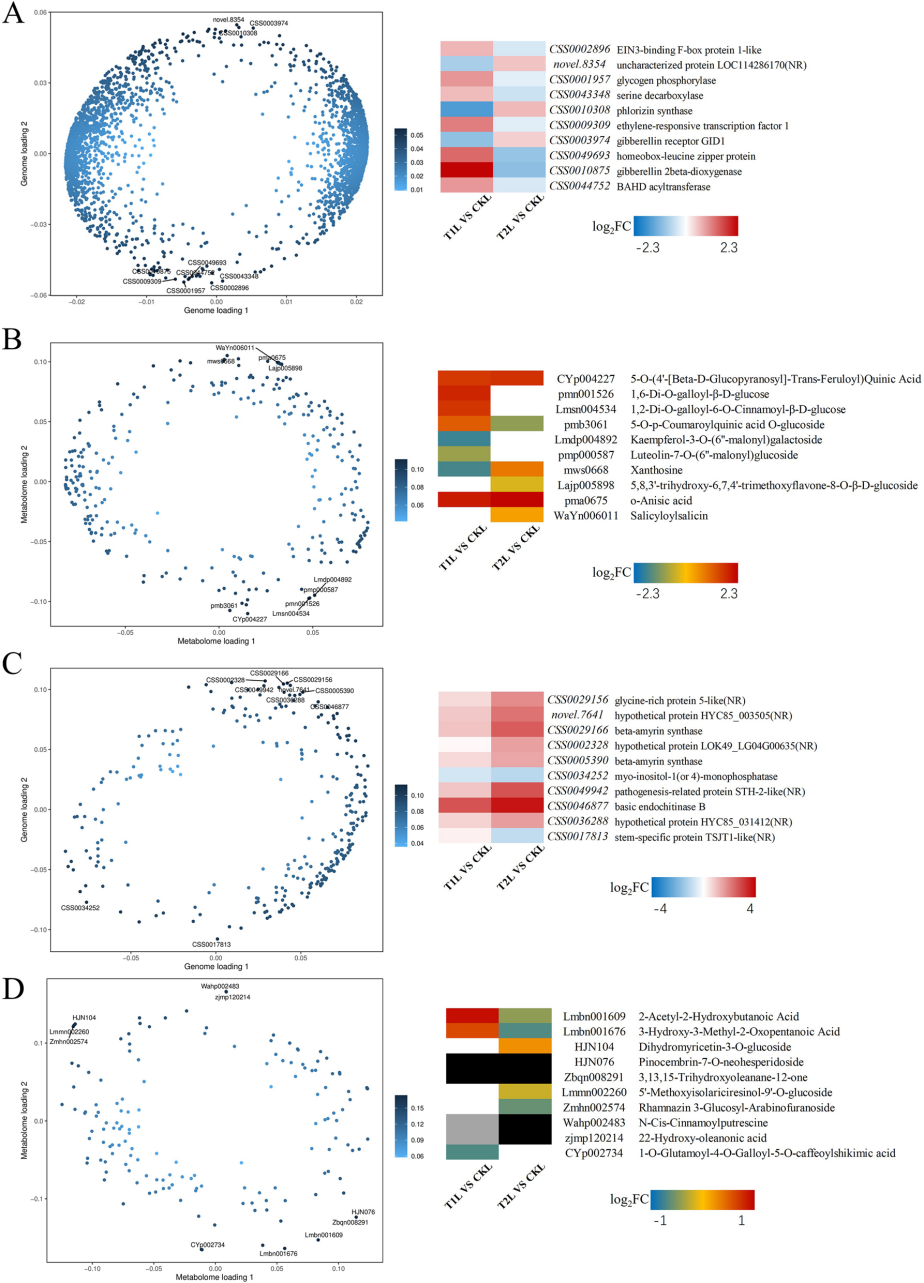
Integrated analysis of gene and metabolite expression in tea leaves and new shoots based on O2PLS under the influence of ZnO NPs. The top ten genes with the greatest impact on metabolite changes in tea leaves and their expression levels (**A**); the top ten metabolites with the greatest impact on gene expression in tea leaves and their expression levels (**B**); the top ten genes with the greatest impact on metabolite changes in new shoots and their expression levels (**C**); the top ten metabolites with the greatest impact on gene expression in new shoots and their expression levels (**D**); white boxes represent metabolites not detected in the treatment group (ZnO NPs); black boxes represent substances not detected in the control group; gray boxes represent substances not detected in both the treatment and control groups. The redder the color in the box, the higher the level of gene expression or the greater the content of the substance; the bluer the color, the lower the level of gene expression or the lesser the content of the substance

To uncover the internal connections between gene expression and metabolite changes in tea plants in response to ZnO NPs, and to determine the degree of association between them, as well as to identify the main genes and metabolites causing this association, we conducted an O2PLS analysis (Fig. 6). Under the influence of ZnO NPs, the top ten genes affecting changes in metabolites in tea leaves and their expression levels are shown in Fig. 6A, while the top ten metabolites affecting transcript expression in tea leaves and their expression levels are shown in Fig. 6B. Similarly, the top ten genes affecting changes in metabolites in new shoots and their expression levels are shown in Fig. 6C, and the top ten metabolites affecting transcript expression in new shoots and their expression levels are shown in Fig. 6D. Interestingly, among the top ten metabolites with the greatest impact on transcript expression in tea leaves, seven are related to starch and sucrose metabolism.

### Analysis of the phyllosphere epiphytic microbial structure of tea plants under the influence of ZnO NPs

#### ASV clustering analysis of epiphytic bacteria and fungi

To investigate the community composition of endophytic microorganisms in the phyllosphere of tea plants under the influence of ZnO NPs, similar to the epiphytic microorganisms, we performed ASV clustering analysis. As shown in Fig. S9A, under the influence of different concentrations of ZnO NPs, the shared ASVs of endophytic bacteria in the tea plant phyllosphere numbered 16, with CKED having 58 unique ASVs, T1ED having 47 unique ASVs, and T2ED having 161 unique ASVs. As depicted in Fig. S9B, under the effect of different concentrations of ZnO NPs, the shared ASVs of endophytic fungi in the tea plant phyllosphere numbered 112, with CKED having 595 unique ASVs, T1ED having 186 unique ASVs, and T2ED having 317 unique ASVs. The results indicate that the use of ZnO NPs altered the number of ASVs.

#### Analysis of alpha diversity in phyllosphere epiphytic bacteria and fungi

To investigate the microbial community diversity under the influence of ZnO nanoparticles, we conducted an Alpha Diversity analysis, the results of which are shown in Table 1. The use of ZnO NPs did not produce a significant difference in the diversity of the tea plant phyllosphere epiphytic bacterial community. Similarly, the T1 treatment did not show a significant difference in the diversity of the phyllosphere epiphytic fungal community, but the T2 treatment significantly reduced the diversity of the phyllosphere epiphytic fungal community.

**Table 1:** The alpha diversity index of phyllosphere epiphytic microorganisms under the influence of ZnO NPs

Sample	Shannon		Simpson		Chao1		Ace	
	Bacteria	Fungi	Bacteria	Fungi	Bacteria	Fungi	Bacteria	Fungi
CKEP	6.82 ± 1.51a	4.04 ± 0.01a	0.89 ± 0.08a	0.89 ± 0.01a	2004.90 ± 730.48a	285.01 ± 32.94a	2004.90 ± 730.48a	319.12 ± 35.23a
T1EP	7.10 ± 1.03a	4.23 ± 0.98a	0.95 ± 0.02a	0.79 ± 0.27a	2367.58 ± 613.91a	275.05 ± 39.51a	2367.58 ± 603.92a	262.65 ± 49.37a
T2EP	5.91 ± 1.41a	2.56 ± 0.59b	0.85 ± 0.12a	0.61 ± 0.19b	4186.13 ± 706.81a	133.63 ± 18.46b	1872.39 ± 376.51a	153.14 ± 35.54b

There were significant differences in values with different letters (p < 0.05)

#### NMDS analysis and permutational MANOVA (*ADONIS*) analysis of phyllosphere epiphytic microorganisms

**Fig. 7:**
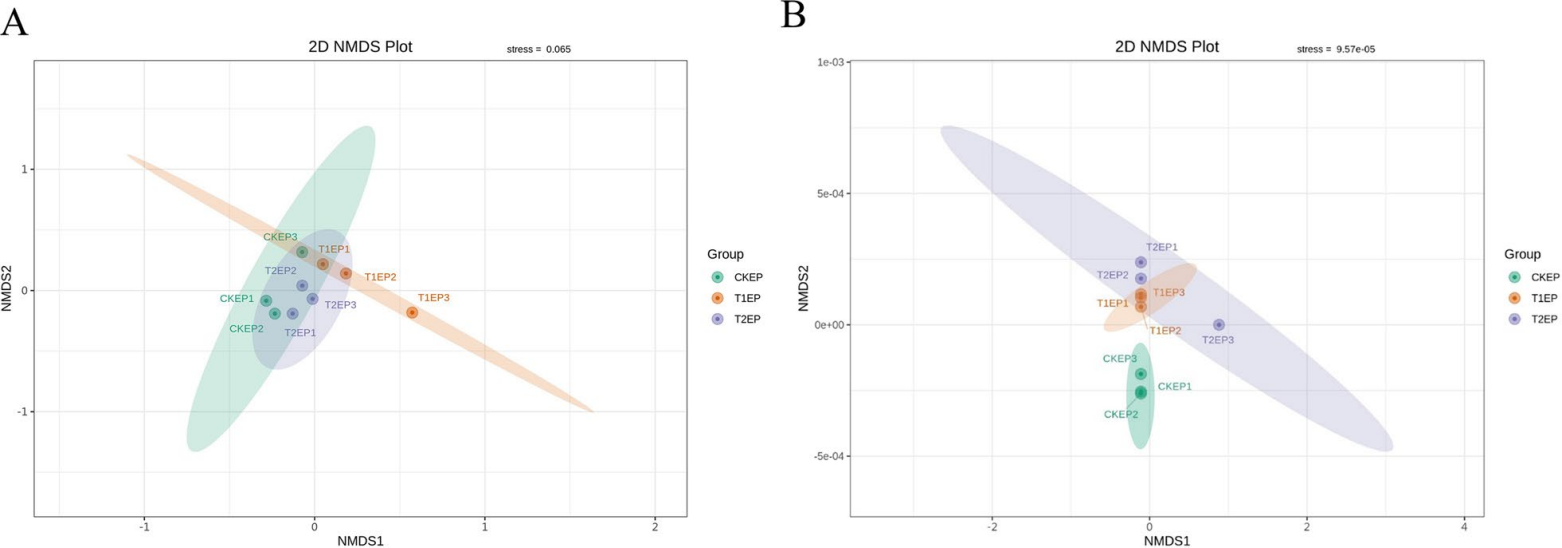
NMDS analysis of tea plant phyllosphere epiphytic bacteria (**A**) and fungi (**B**) under the influence of ZnO nanoparticles. Each point in the figure represents a sample, with the distance between points indicating the degree of difference. Samples from the same group are represented by the same color

To demonstrate the extent of differences in phyllosphere epiphytic microorganisms under the influence of ZnO NPs, we performed NMDS analysis, the results of which are shown in Fig. 7. The stress values for the NMDS analysis of epiphytic bacteria and fungi were 0.065 and 9.57 × 10^–5^ (Fig. 7B), respectively, both of which are less than 0.2 (a stress value less than 0.2 indicates that NMDS can accurately reflect the degree of differences between samples). Concurrently, the Principal Coordinates Analysis (PCoA) of phyllosphere epiphytic fungi showed a P-value of 0.011, indicating a significant difference, similar to the Alpha Diversity analysis. At the same time, we also conducted an ADONIS analysis, the results of which are shown in Table S3.

#### Genus-level clustering analysis of epiphytic bacteria and fungi

**Fig. 8:**
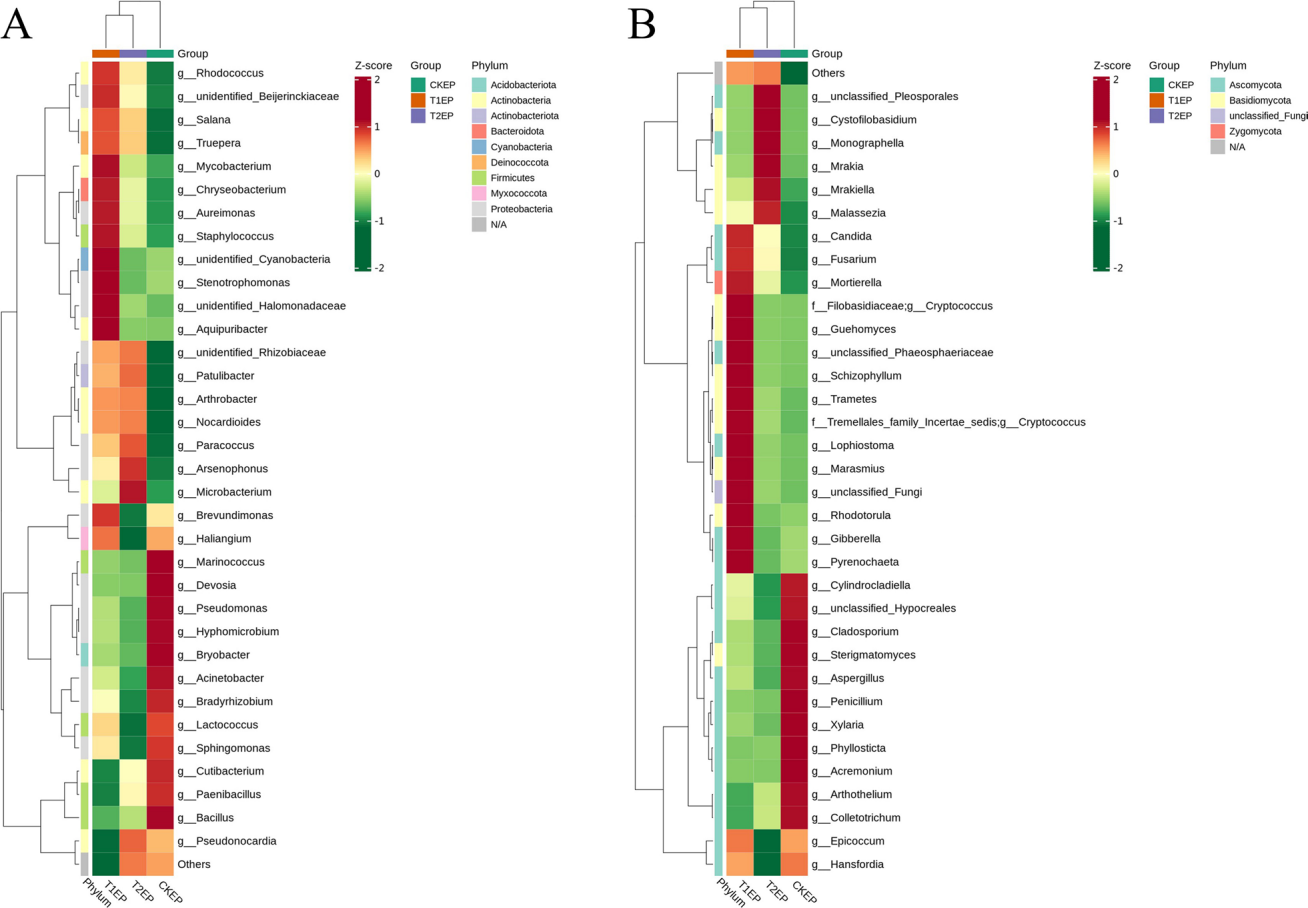
Heatmaps of ASV-based clustering of epiphytic bacterial (**A**) and fungal (**B**) communities in the phyllosphere of tea plants under the influence of ZnO NPs. Vertically, the heatmaps display sample information, and horizontally, they show species classification information. The clustering trees within the figure represent species clustering; the values in the heatmap correspond to Z-Score normalized relative quantitative data

To analyze the differences in the community composition of epiphytic microbes in the phyllosphere of tea plants under different concentrations of ZnO NPs, based on the sum of the quantitative values of microorganisms across all samples, we selected the top 35 microbial taxa. Utilizing their quantitative data in each sample, we performed clustering at both the species and sample levels and generated a heatmap. This visualization facilitates the identification of which species are more abundant or less prevalent in certain samples, and allows for the assessment of the clustering relationships between samples. As shown in Fig. 8A, the bacterial communities in CKEP were mainly clustered in genera such as *Marinococcus, Devosia, Pseudomonas, Hyphomicrobium, Bryobacter, Acinetobacter, Bradyrhizobium, Lactococcus, Sphingomonas, Cutibacterium, Paenibacillus*, and *Bacillus*. The bacterial communities in T1EP were mainly clustered in *Rhodococcus*, unidentified *Beijerinckiaceae*, *Salana, Truepera, Mycobacterium, Chryseobacterium, Aureimonas, Staphylococcus,* unidentified *Cyanobacteria*, *Stenotrophomonas*, unidentified *Halomonadaceae, Aquipuribacter,* unidentified *Rhizobiaceae, Patulibacter, Arthrobacter, Nocardioides, Paracoccus, Arsenophonus, Microbacterium,* and *Brevundimonas*. The bacterial communities in T2EP were enriched in Arsenophonus and Microbacterium. As shown in Fig. 8B, the fungal communities in CKEP were mainly clustered in *Cylindrocladiella,* unclassified *Hypocreales, Cladosporium, Sterigmatomyces, Aspergillus, Penicillium, Xylaria, Phyllosticta, Acremonium, Arthothelium,* and *Colletotrichum*. The fungal communities in T1EP were mainly clustered in *Candida, Fusarium, Mortierella, Cryptococcus* (*f Filobasidiaceae*)*, Guehomyces,* unclassified *Phaeosphaeriaceae, Schizophyllum, Trametes, Cryptococcus* (*Tremellales family Incertae sedis*)*, Lophiostoma, Marasmius,* unclassified *Fungi, Rhodotorula, Gibberella,* and *Pyrenochaeta*. The fungal communities in T2EP were mainly enriched in unclassified *Pleosporales, Cystofilobasidium, Monographella, Mrakia, Mrakiella, and Malassezia*. The clustering results show significant differences in the dominant microbial species, indicating that different concentrations of ZnO NPs have selective effects on epiphytic bacteria and fungi.

#### Functional clustering analysis of phyllosphere epiphytic bacteria and fungi

**Fig. 9:**
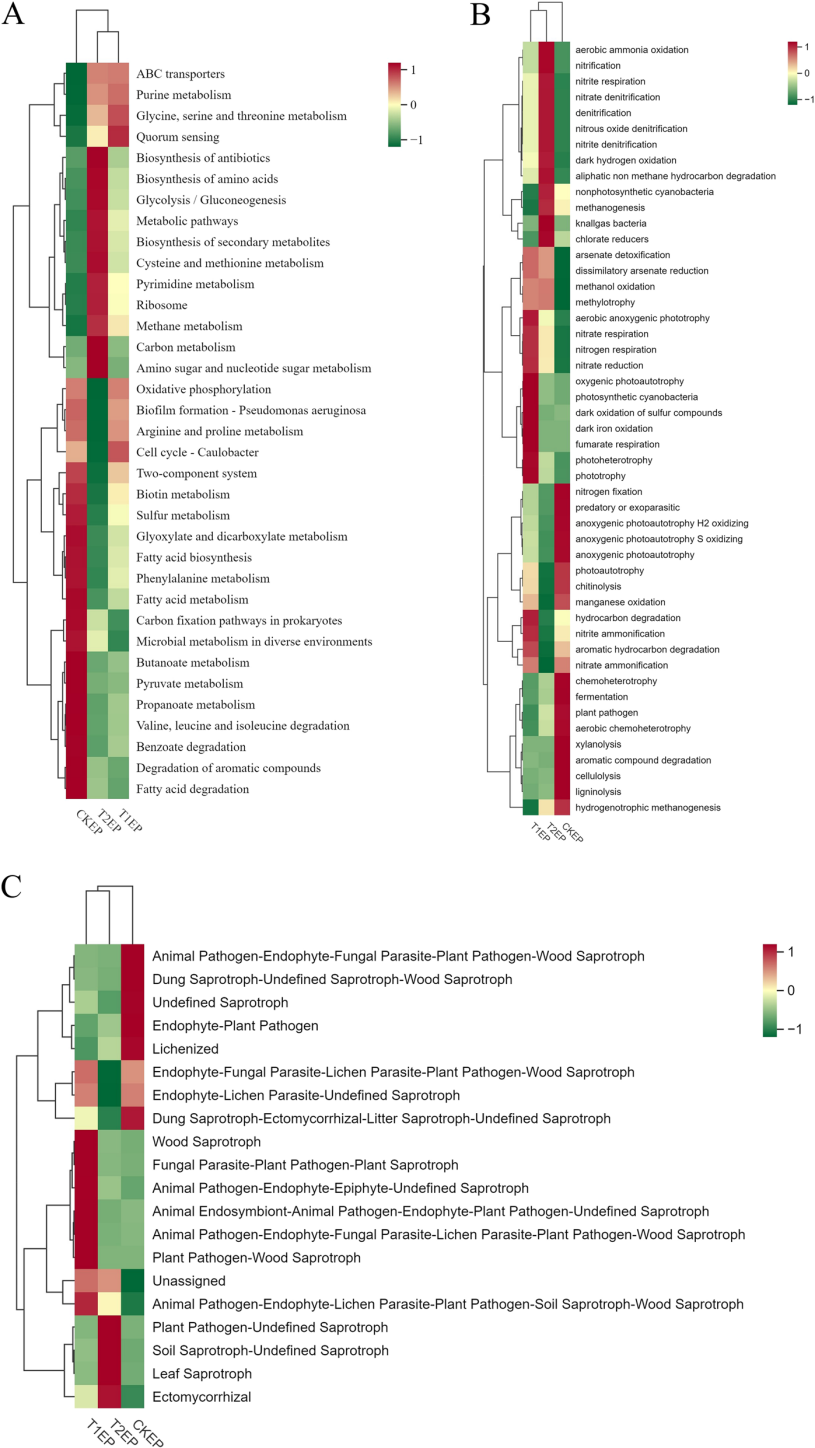
The heatmaps for the functional annotation clustering of epiphytic bacteria on tea plant leaves across different groups are based on Tax4Fun2 (**A**); the functional annotation clustering of epiphytic bacteria based on FAPROTAX (**B**); and the functional annotation clustering of epiphytic fungi based on FunGuild (**C**). Horizontally, the heatmaps display functional annotation information, while vertically, they show sample information. The squares indicate relative abundance, with colors shifting towards red for higher relative abundance and towards green for lower relative abundance

To analyze the impact of ZnO-NPs on the functional potential of epiphytic bacteria associated with tea plant leaves, we employed Tax4Fun2 and FAPROTAX (Functional Annotation of Prokaryotic Taxa) for functional annotation and clustering. As shown in Fig. 9A, the bacterial functions in CKEP were mainly enriched in Microbial metabolism in diverse environments, Carbon fixation pathways in prokaryotes, Valine, leucine and isoleucine degradation, Propanoate metabolism, Benzoate degradation, Butanoate metabolism, Pyruvate metabolism, Degradation of aromatic compounds, Fatty acid degradation, Glyoxylate and dicarboxylate metabolism, Fatty acid biosynthesis, Phenylalanine metabolism, Fatty acid metabolism, Sulfur metabolism, and Biotin metabolism. The bacterial functions in T1EP were primarily focused on Quorum sensing. The bacterial functions in T2EP were mainly concentrated in Carbon metabolism, Amino sugar and nucleotide sugar metabolism, Methane metabolism, Pyrimidine metabolism, Ribosome, Biosynthesis of amino acids, Glycolysis/Gluconeogenesis, Biosynthesis of antibiotics, Metabolic pathways, Cysteine and methionine metabolism, Biosynthesis of secondary metabolites. As depicted in Fig. 9B, the bacterial functions in CKEP were mainly enriched in chemoheterotrophy, fermentation, plant pathogen, aerobic chemoheterotrophy, xylanolysis, aromatic compound degradation, cellulolysis, ligninolysis, hydrogenotrophic methanogenesis, nitrogen fixation, predatory or exoparasitic, anoxygenic photoautotrophy H2 oxidizing, anoxygenic photoautotrophy S oxidizing, anoxygenic photoautotrophy, photoautotrophy, chitinolysis, manganese oxidation. The bacterial functions in T1EP were primarily focused on aerobic anoxygenic phototrophy, nitrate respiration, nitrogen respiration, nitrate reduction, oxygenic photoautotrophy, photosynthetic cyanobacteria, dark oxidation of sulfur compounds, dark iron oxidation, fumarate respiration, photoheterotrophy, phototrophy, hydrocarbon degradation, nitrite ammonification. The bacterial functions in T2EP were mainly concentrated in aerobic ammonia oxidation, nitrification, nitrite respiration, nitrate denitrification, denitrification, nitrous oxide denitrification, nitrite denitrification, dark hydrogen oxidation, aliphatic non methane hydrocarbon degradation, nonphotosynthetic cyanobacteria, methanogenesis, knallgas bacteria, chlorate reducers. Notably, the plant pathogen function, which was significantly enriched in CKEP, had a very low abundance in T1EP and T2EP.

To analyze the impact of ZnO-NPs on the functional potential of epiphytic fungi associated with tea plant leaves, we used FunGuild for functional annotation and clustering. As shown in Fig. 9C, the fungal functions in CKEP were mainly focused on Dung Saprotroph-Undefined Saprotroph-Wood Saprotroph, Animal Pathogen-Endophyte-Fungal Parasite-Plant Pathogen-Wood Saprotroph, Undefined Saprotroph, Lichenized, Endophyte-Plant Pathogen, Dung Saprotroph-Ectomycorrhizal-Litter Saprotroph-Undefined Saprotroph. The fungal functions in T1EP were primarily enriched in Plant Pathogen-Wood Saprotroph, Endophyte-Lichen Parasite-Plant Pathogen-Undefined Saprotroph, Animal Pathogen-Endophyte-Fungal Parasite-Lichen Parasite-Plant Pathogen-Wood Saprotroph, Animal Endosymbiont-Animal Pathogen-Endophyte-Plant Pathogen-Undefined Saprotroph, Animal Pathogen-Endophyte-Epiphyte-Undefined Saprotroph, Wood Saprotroph, Animal Pathogen-Endophyte-Lichen Parasite-Plant Pathogen-Soil Saprotroph-Wood Saprotroph, and Fungal Parasite-Plant Pathogen-Plant Saprotroph. The fungal functions in T2EP were mainly concentrated in Ectomycorrhizal, Plant Pathogen-Undefined Saprotroph, Leaf Saprotroph, and Soil Saprotroph-Undefined Saprotroph.

### Analysis of the phyllosphere endophytic microbial structure of tea plants under the influence of ZnO NPs

#### ASV clustering analysis of endophytic bacteria and fungi

To study the community composition of endophytic microorganisms in the phyllosphere of tea plants under the influence of ZnO NPs, similar to the epiphytic microorganisms, we performed ASV clustering analysis. As shown in Fig. S9A, under the influence of different concentrations of ZnO NPs, the shared ASVs of endophytic bacteria in the tea plant phyllosphere numbered 16, with CKED having 58 unique ASVs, T1ED having 47 unique ASVs, and T2ED having 161 unique ASVs. As shown in Fig. S9B, under the effect of different concentrations of ZnO NPs, the shared ASVs of endophytic fungi in the tea plant phyllosphere numbered 112, with CKED having 595 unique ASVs, T1ED having 186 unique ASVs, and T2ED having 317 unique ASVs. The results indicate that the use of ZnO NPs altered the number of ASVs.

#### Analysis of alpha diversity in endophytic bacteria and fungi of the phyllosphere

To explore the microbial community diversity under the influence of ZnO nanoparticles, we conducted an Alpha Diversity analysis, the results of which are shown in Table 2. The use of ZnO NPs did not produce a significant difference in the diversity of the tea plant phyllosphere endophytic bacterial community. However, there were significant differences in the Chao1 and ACE indices for the phyllosphere endophytic fungi.

**Table 2:** The alpha diversity index of phyllosphere endophytic microorganisms under the influence of ZnO NPs

Sample	Shannon		Simpson		Chao1		Ace	
	Bacteria	Fungi	Bacteria	Fungi	Bacteria	Fungi	Bacteria	Fungi
CKEP	0.42 ± 0.18a	4.67 ± 1.70a	0.14 ± 0.08a	0.80 ± 0.25a	39.35 ± 5.14a	383.88 ± 19.21a	40.84 ± 5.46a	391.56 ± 24.34a
T1EP	0.50 ± 0.07a	4.45 ± 0.20a	0.17 ± 0.03a	0.87 ± 0.02a	34.53 ± 7.39a	225.51 ± 330.93b	35.43 ± 7.74a	236.20 ± 33.06b
T2EP	0.68 ± 0.25a	4.78 ± 0.04a	0.25 ± 0.12a	0.93 ± 0.01a	44.97 ± 29.11a	270.34 ± 108.30ab	47.62 ± 31.88a	288.74 ± 20.48ab

There were significant differences in values with different letters (p < 0.05)

#### NMDS analysis and permutational MANOVA (*ADONIS*) analysis of endophytic microorganisms in the phyllosphere

**Fig. 10:**
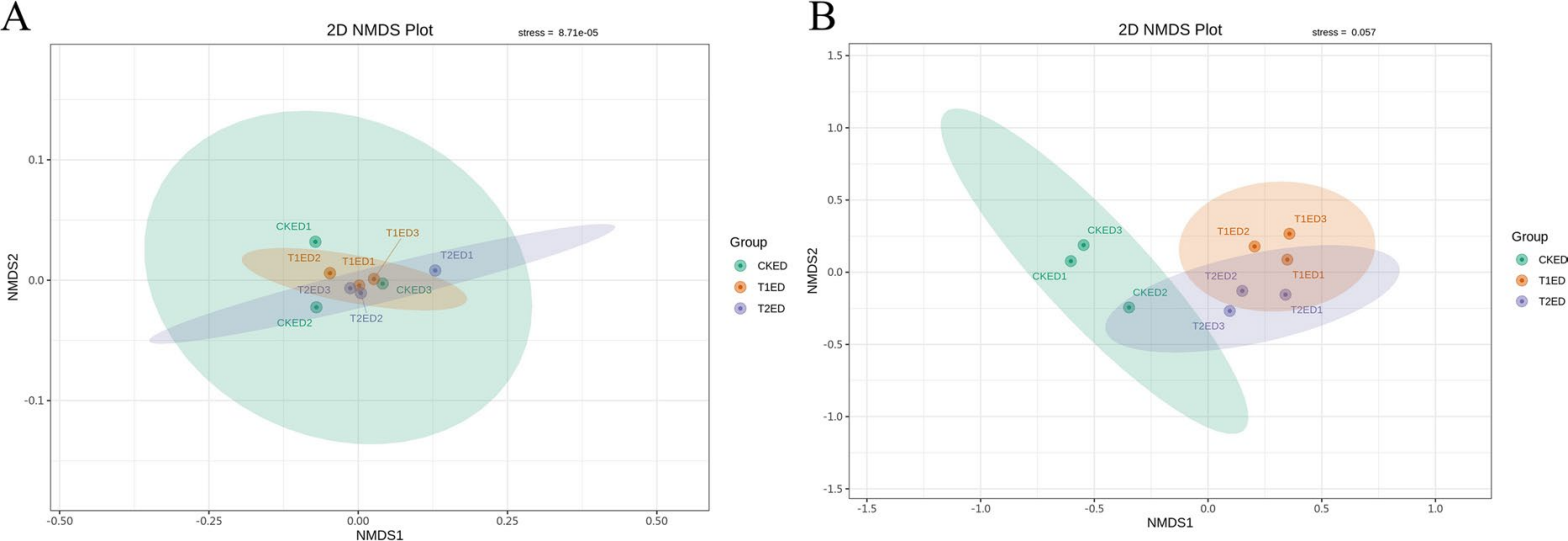
NMDS analysis of tea plant phyllosphere endophytic bacteria (**A**) and endophytic fungi (**B**) under the influence of ZnO NPs. Each point in the figure represents a sample, with the distance between points indicating the degree of difference. Samples from the same group are represented by the same color

To demonstrate the extent of differences in phyllosphere endophytic microorganisms under the influence of ZnO NPs, we performed NMDS analysis, the results of which are shown in Fig. 10. The stress values for the NMDS analysis of endophytic bacteria and fungi were 8.71 × 10^–5^ (Fig. 10A) and 0.057 (Fig. 10B), respectively, both of which are less than 0.2 (a stress value less than 0.2 indicates that NMDS can accurately reflect the degree of differences between samples). Concurrently, the Principal Coordinates Analysis (PCoA) of phyllosphere endophytic fungi showed a P-value of 0.001, indicating a significant difference, similar to the Alpha Diversity analysis. At the same time, we also conducted an ADONIS analysis, the results of which are shown in Table S4.

#### Genus-level clustering analysis of endophytic bacteria and fungi and their association with starch and sucrose metabolism products

**Fig. 11:**
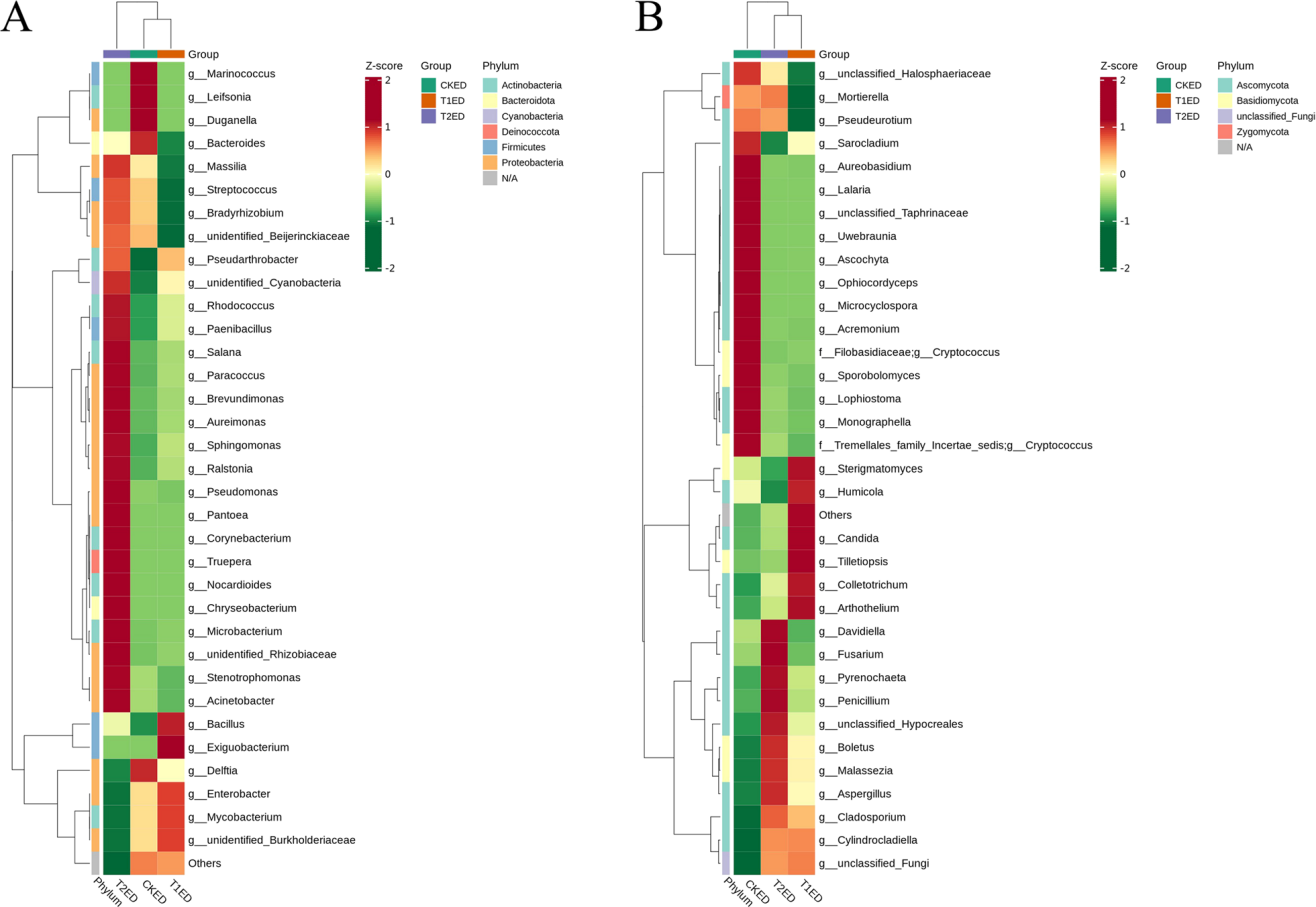
Heatmaps of ASV-based clustering of endophytic bacterial (**A**) and fungal (**B**) communities in the phyllosphere of tea plants under the influence of ZnO NPs. Vertically, the heatmaps display sample information, and horizontally, they show species classification information. The clustering trees within the figure represent species clustering; the values in the heatmap correspond to Z-Score normalized relative quantitative data

To analyze the differences in the community composition of endophytic microbes in the phyllosphere of tea plants under different concentrations of ZnO NPs, we performed clustering analysis at the genus level. As shown in Fig. 11A, the bacterial communities in CKED were mainly clustered in *Marinococcus, Leifsonia, Duganella, Bacteroides,* and *Delftia*. The bacterial communities in T1ED were mainly clustered in *Bacillus, Exiguobacterium, Delftia, Enterobacter, Mycobacterium, and* unidentified *Burkholderiaceae*. The bacterial communities in T2ED were mainly clustered in *Massilia, Rhodococcus, Paenibacillus, Salana, Paracoccus, Brevundimonas, Aureimonas, Sphingomonas, Ralstonia, Pseudomonas, Pantoea, Corynebacterium, Truepera, Nocardioides, Chryseobacterium, Microbacterium,* unidentified *Rhizobiaceae, Stenotrophomonas,* and *Acinetobacter*. As shown in Fig. 11B, the fungal communities in CKED were mainly clustered in *Aureobasidium, Lalaria,* unclassified *Taphrinaceae, Uwebraunia, Ascochyta, Ophiocordyceps, Microcyclospora, Acremonium, Cryptococcus (Filobasidiaceae), Sporobolomyces, Lophiostoma, Monographella,* and *Cryptococcus* (*Tremellales family Incertae sedis*). The fungal communities in T1ED were mainly clustered in S*terigmatomyces, Humicola, Candida, Tilletiopsis, Colletotrichum,* and *Arthothelium*. The fungal communities in T2ED were mainly enriched in *Davidiella, Fusarium, Pyrenochaeta, Penicillium,* unclassified *Hypocreales, Boletus, Malassezia, and Aspergillus*. The clustering results show significant differences in the dominant microbial species, indicating that different concentrations of ZnO NPs have selective effects on endophytic bacteria and fungi.

**Fig. 12:**
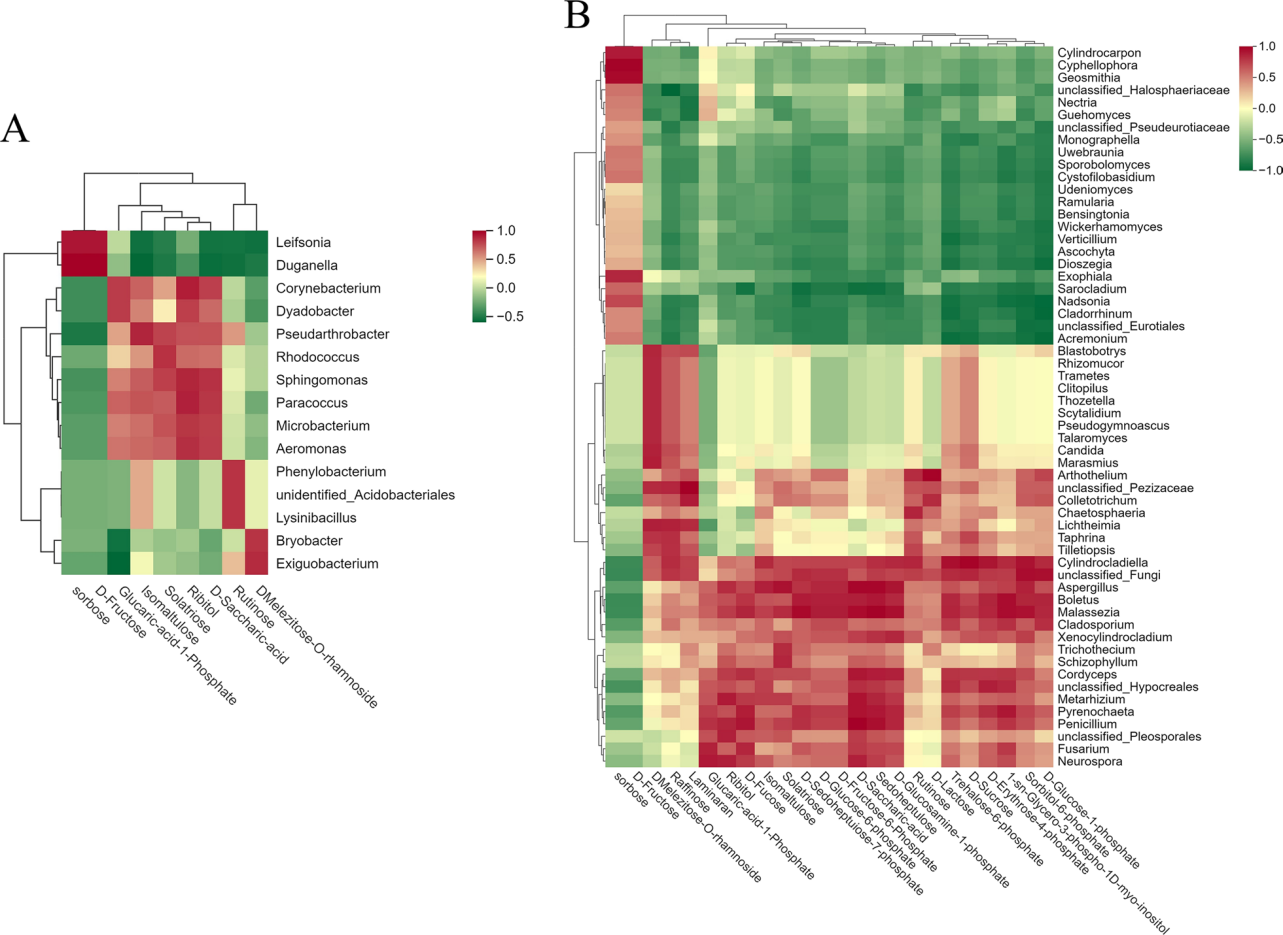
Spearman correlation between endophytic bacteria (**A**) and fungi (**B**) in the phyllosphere of tea plants under the influence of ZnO NPs and differential starch and sucrose metabolism products. The selected threshold for p-value is 0.05, and for cor.value is 0.80

To investigate the correlation between endophytic microbes in the phyllosphere of tea plants and differential starch and sucrose metabolism products under the influence of ZnO NPs, we conducted a Spearman correlation hierarchical clustering analysis (Fig. 12). Several endophytic bacteria (Fig. 12A) and fungi (Fig. 12B) showed significant correlations with sugar metabolism (with fungi demonstrating stronger correlations). Specific correlation coefficients and significance values between each microbe and sugars are presented in Table S5. *Notably, Taphrina, Cylindrocladiella, Aspergillus, Boletus, Malassezia, Cladosporium, Xenocylindrocladium, Cordyceps,* and *Pyrenochaeta* showed significant positive correlations with sucrose.

### Functional Clustering Analysis of Phyllosphere Endophytic *Bacteria* and Fungi

**Fig. 13:**
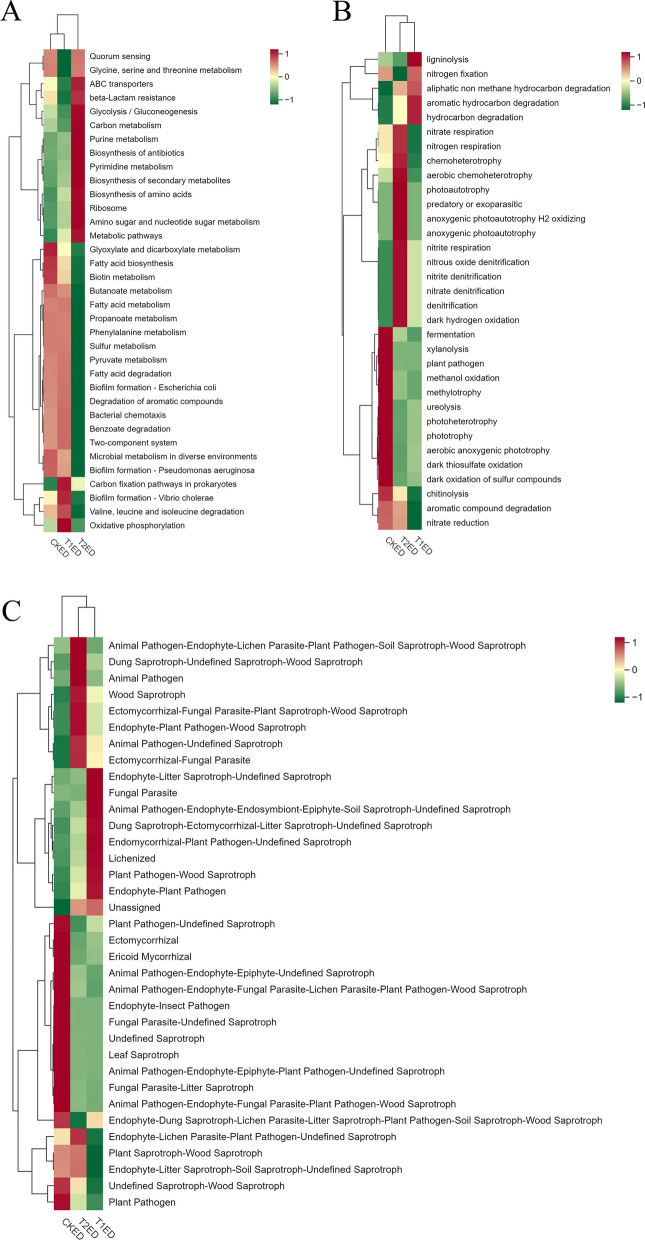
Clustered heatmap of functional annotation of endophytic bacteria in the phyllosphere among different groups based on Tax4Fun2 (**A**); Clustered heatmap of functional annotation of endophytic bacteria in the phyllosphere among different groups based on FAPROTAX (**B**); Clustered heatmap of functional annotation of endophytic fungi in the phyllosphere among different groups based on FunGuild (**C**). Horizontally, the heatmaps display functional annotation information, while vertically, they show sample information. The squares indicate relative abundance, with colors shifting towards red for higher relative abundance and towards green for lower relative abundance

To analyze the effects of ZnO NPs on the functional capabilities of endophytic bacteria in the phyllosphere of tea plants, similar to the approach taken with endophytic microorganisms, we employed Tax4Fun2 and FAPROTAX for functional annotation and clustering. As shown in Fig. 13A, the bacterial functions in CKED were mainly enriched in Fatty acid biosynthesis, Biotin metabolism, and Glyoxylate and dicarboxylate metabolism. The bacterial functions in T1ED were primarily concentrated in Carbon fixation pathways in prokaryotes, Biofilm formation—Vibrio cholerae, and Oxidative phosphorylation. The bacterial functions in T2ED were mainly focused on Metabolic pathways, Biosynthesis of amino acids, Ribosome, Amino sugar and nucleotide sugar metabolism, Biosynthesis of antibiotics, Purine metabolism, Pyrimidine metabolism, Biosynthesis of secondary metabolites, ABC transporters, Glycolysis/Gluconeogenesis, and Carbon metabolism.As depicted in Fig. 13B, the bacterial functions in CKED were mainly enriched in ureolysis, photoheterotrophy, phototrophy, aerobic anoxygenic phototrophy, dark thiosulfate oxidation, dark oxidation of sulfur compounds, methanol oxidation, methylotrophy, xylanolysis, plant pathogen, fermentation. The bacterial functions in T1ED were predominantly clustered in ligninolysis, aliphatic non-methane hydrocarbon degradation, hydrocarbon degradation. The bacterial functions in T2ED were mainly focused on nitrate respiration, nitrogen respiration, chemoheterotrophy, aerobic chemoheterotrophy, anoxygenic photoautotrophy H2 oxidizing, anoxygenic photoautotrophy, predatory or exoparasitic, photoautotrophy, denitrification, dark hydrogen oxidation, nitrate denitrification, nitrite denitrification, nitrous oxide denitrification, nitrite respiration. Notably, the plant pathogen function, significantly enriched in CKED, was found to be of low abundance in T1ED and T2ED.

To analyze the impact of ZnO NPs on the functions of phyllosphere endophytic fungi in tea plants, similar to epiphytic microorganisms, we used FunGuild for functional annotation and clustering. As shown in Fig. 13C, the fungal functions in CKED were mainly clustered in Plant Pathogen-Undefined Saprotroph, Ectomycorrhizal, Ericoid Mycorrhizal, Undefined Saprotroph, Endophyte-Insect Pathogen, Fungal Parasite-Undefined Saprotroph, Leaf Saprotroph, Animal Pathogen-Endophyte-Epiphyte-Plant Pathogen-Undefined Saprotroph, Fungal Parasite-Litter Saprotroph, Animal Pathogen-Endophyte-Fungal Parasite-Plant Pathogen-Wood Saprotroph, Animal Pathogen-Endophyte-Fungal Parasite-Lichen Parasite-Plant Pathogen-Wood Saprotroph, Animal Pathogen-Endophyte-Epiphyte-Undefined Saprotroph, Plant Pathogen. The fungal functions in T1ED were mainly enriched in Endophyte-Litter Saprotroph-Undefined Saprotroph, Fungal Parasite, Animal Pathogen-Endophyte-Endosymbiont-Epiphyte-Soil Saprotroph-Undefined Saprotroph, Endomycorrhizal-Plant Pathogen-Undefined Saprotroph, Lichenized, Dung Saprotroph-Ectomycorrhizal-Litter Saprotroph-Undefined Saprotroph, Plant Pathogen-Wood Saprotroph, Endophyte-Plant Pathogen. The fungal functions in T2ED were mainly enriched in Ectomycorrhizal-Fungal Parasite-Plant Saprotroph-Wood Saprotroph, Endophyte-Plant Pathogen-Wood Saprotroph, Dung Saprotroph-Undefined Saprotroph-Wood Saprotroph, Animal Pathogen, Animal Pathogen-Endophyte-Lichen Parasite-Plant Pathogen-Soil Saprotroph-Wood Saprotroph. Notably, the plant pathogen function, which was significantly enriched in CKED, had a very low abundance in T1ED and T2ED.

## Discussion

Photosynthesis is pivotal for plant vitality and productivity, and its optimization is crucial for enhancing crop quality and yield [40, 41]. Our study reveals that ZnO nanoparticles positively influence tea plant photosynthesis by improving key physiological and chlorophyll fluorescence parameters, RubisCO enzyme content, gene expression related to photosynthesis, and energy reserves. Specifically, ZnO NPs at 100 mg L^−1^ significantly enhanced the photosynthetic capacity of tea plants, as demonstrated by increased Photo, Cond, and Trmmol values, which represent plant growth potential, CO_2_ utilization efficiency, and water circulation intensity, respectively [42–45]. Notably, Photo was also significantly improved at 50 mg L^−1^ ZnO NPs.

ZnO NPs were found to enhance key photosynthetic enzyme levels, such as RubisCO, which is crucial for CO_2_ assimilation, particularly in C3 plants like tea. The study observed a significant increase in RubisCO content with ZnO NP treatment, correlating with improved photosynthetic rates [46, 47]. Similarly, FBP and PEPC, enzymes involved in sugar metabolism and malate synthesis respectively, also showed increased levels, indicating a broader impact on photosynthetic and metabolic processes [48–51]. Additionally, ZnO NPs promoted chlorophyll synthesis, leading to improved chlorophyll fluorescence parameters such as Fv/Fm, qP, and Y(II), signifying a positive effect on photosystem II efficiency [52]. Gene expression analysis post ZnO NP application revealed upregulation in genes related to the light-harvesting complex II (LHCB), essential for capturing light energy and driving photosynthesis [53, 54]. This suggests that ZnO NPs may influence the light-dependent reactions and overall photosynthetic capacity.

ZnO NPs have been shown to enhance the accumulation of sucrose, the primary photosynthetic product and transport sugar in plants, which facilitates carbon and energy transfer to sink tissues [55]. Our findings demonstrate that ZnO NPs significantly increase sucrose and other starch metabolism-related metabolites in tea plant leaves, with a notable twofold rise in content. This metabolic enhancement is corroborated by WGCNA and O2PLS analyses, which reveal strong associations between ZnO NP treatment, and the upregulated genes involved in starch and sucrose metabolism.

Our findings suggest ZnO NPs can enhance photosynthesis in C3 tea plants. While distinct from C3 plants, C4 species may also benefit from ZnO NPs, as they improve fundamental photosynthetic components such as enzyme activities and chlorophyll content. For instance, maize, which are C4 plants, have shown positive growth and biochemical responses to ZnO NPs [56].

The study also highlights the role of sucrose in bud development, acting as a signaling molecule that regulates gene expression related to growth and photosynthesis. Furthermore, our results suggest that ZnO NPs induce auxin production, a hormone essential for bud development and germination processes [57–60]. The observed increase in auxin content upon ZnO NP exposure implies a potential mechanism by which ZnO NPs may stimulate tea bud development and contribute to plant growth cycles.

Application of ZnO NPs has been found to influence the mineral element composition in tea plants, enhancing zinc, molybdenum, and copper levels in the leaves, which are vital for enzyme activation, carbohydrate metabolism, chlorophyll synthesis, and photosynthesis [61, 62]. While zinc content in new shoots did not significantly change, possibly due to the timing and direct contact of ZnO NP application, the consistent trend in calcium and magnesium levels underlines their role in plant growth [63]. Notably, ZnO NPs decreased aluminum levels in leaves, suggesting a mitigating effect on potential aluminum toxicity [64, 65]. Additionally, an increase in selenium and iron in new shoots was observed at 50 mg L^−1^ ZnO NP treatment, which may contribute to tea quality and health benefits [66, 67].

The use of ZnO NPs altered the dominant community composition of phyllosphere epiphytic microorganisms on tea plants, with different dominant microbial community structures at varying concentrations. Alpha Diversity analysis indicated that the use of ZnO NPs did not significantly affect the diversity of the epiphytic bacterial community on tea plant leaves, however, 100 mg L^−1^ ZnO NPs reduced the diversity of epiphytic fungi. Genus-level clustering analysis showed that the composition of the epiphytic microbial community changed significantly under different concentrations of ZnO NPs, suggesting that ZnO NPs have a selective effect on epiphytic bacteria and fungi. This is consistent with previous studies that metal nanoparticles can affect the composition and abundance of microbial communities, potentially related to the antimicrobial properties of nanoparticles [68]. Similarly, the varying abundance of specific taxa suggests that ZnO NPs may exert selective pressure, favoring certain epiphytic microorganisms to become dominant [69]. For instance, *Stenotrophomonas* and *Microbacterium* are both known to be beneficial to plants, improving plant living conditions through mechanisms such as hormone production and nitrogen fixation [70, 71].

LefSe analysis identified several key biomarkers that could differentiate between communities of epiphytic bacteria and fungi at different concentrations. The presence of specific taxa such as *Marinococcus* and *Microbacterium* in different treatments highlights the potential of ZnO NPs to affect microbial community structure. This is similar to findings in Aconitum where ZnO NPs were shown to affect microbial community structure and plant secondary metabolism [72]. Random forest analysis identified *Marinococcus* and *Microbacterium* as key species contributing to the greatest variation in microbial community structure under different ZnO NP concentrations. This suggests that ZnO NPs may influence specific taxa within the phyllosphere epiphytic microbial community, which could have implications for the overall health and disease resistance of tea plants [73].

Functional clustering analysis revealed changes in metabolic pathways and ecological functions within the epiphytic bacterial and fungal communities. Notably, the reduction in plant pathogen functions in epiphytic microorganisms at two concentrations of ZnO NPs may indicate their potential role in plant disease management. At the same time, the enrichment of nutrient cycling and metabolism-related functions in phyllosphere epiphytic microorganisms under the influence of ZnO NPs suggests that ZnO NPs may also regulate microbial communities to contribute to plant health [74].

The use of ZnO NPs altered the dominant community composition of endophytic microorganisms in the phyllosphere of tea plants, with different dominant microbial community structures at varying concentrations. Cluster analysis at the genus level revealed significant differences in the dominant species of endophytic microorganisms under different concentrations of ZnO NPs. For example, at 0 mg L^−1^ ZnO NPs, the endophytic bacterial community was mainly composed of genera such as *Marinococcus*, *Leifsonia*, and *Duganella*, while at 50 mg L^−1^ ZnO NPs, it was primarily composed of *Bacillus*, *Exiguobacterium*, *Delftia*, and others. These results are consistent with previous studies, indicating that nanoparticles have a selective effect on microbial communities [75]. For example, after the use of ZnO NPs, *Bacillus* becomes a dominant species. *Bacillus* is a beneficial phyllosphere microorganism that can promote plant growth and photosynthesis through multiple mechanisms [76]. Additionally, under salt stress conditions, *Bacillus* can improve the growth of radish plants by increasing both growth and photosynthetic pigment content [77]. At the same time, plants inoculated with *Bacillus* can enhance gas exchange, increase CO_2_ assimilation, and improve the growth parameters of chili plants [78].

Alpha diversity analysis and NMDS analysis further confirmed significant differences in the diversity and structure of endophytic microbial communities under different treatments. This is in line with previous studies that reported significant changes in microbial diversity and community structure upon exposure to nanoparticles [79, 80].

Differential endophytic microbial biomarkers screened through LefSe analysis, as well as key species identified by random forest analysis (such as *Sphingomonas* and *Colletotrichum*), suggest that ZnO NPs may affect specific microbial taxa, which could impact the overall health and disease resistance of tea plants [73]. Furthermore, functional clustering analysis revealed the effects of ZnO NPs on the functional capabilities of endophytic bacteria and fungi. The functions of endophytic bacteria in the phyllosphere not treated with ZnO NPs were mainly enriched in fatty acid synthesis, biotin metabolism, and glyoxylate and dicarboxylate metabolism, while after ZnO NPs treatment, they were enriched in carbon fixation pathways, biofilm formation, and oxidative phosphorylation. More importantly, the abundance of plant pathogens identified by FAPROTAX decreased after the use of ZnO NPs. This suggests that ZnO NPs can not only change the structure of endophytic microbial communities in the phyllosphere but can also regulate their functions, which may have profound effects on the functionality and health of the tea plant ecosystem [81].

In summary, the use of ZnO NPs has improved the community composition of both phyllosphere epiphytic and endophytic microorganisms on tea plants. Many beneficial microorganisms have become dominant, and there is an inhibitory effect on potential pathogenic plant pathogens, which may be related to the antimicrobial properties of ZnO NPs. This suggests that ZnO NPs could play a role in promoting plant health and protecting against diseases, potentially offering an advantage for sustainable agriculture practices.

The integration of ZnO NPs into agricultural systems offers potential growth and health benefits for plants, yet it is accompanied by considerations of cost, environmental impact, and safety [82]. The financial aspect is a critical factor, as the synthesis and application of ZnO NPs must be cost-effective to be feasible for large-scale use [83]. Environmental concerns also play a significant role, particularly regarding the potential for nanoparticle accumulation in the ecosystem and their long-term effects on soil and plant health [84]. Moreover, the interaction of NPs with plants and soil microorganisms is complex, requiring extensive research to ensure that applications are safe and beneficial [10]. Regulatory and public acceptance issues are further challenges that must be addressed to facilitate the responsible adoption of nanotechnologies in agriculture.

## Conclusions

This study investigated the effects of ZnO NPs on tea plants, focusing on photosynthesis (photosynthetic physiological parameters, photosynthetic enzymes, chlorophyll fluorescence parameters), new shoot germination, transcriptome (leaves and new shoots), metabolome (leaves and new shoots), mineral element content (leaves and new shoots), and phyllosphere microbial communities (epiphytic and endophytic microorganisms). The study showed that (1). ZnO NPs enhanced the photosynthesis of tea plants by upregulating the expression of some genes related to photosynthesis and increasing the accumulation of photosynthetic products; (2). ZnO NPs increased the auxin content in tea plants, promoting the development of new shoots; (3). ZnO NPs altered the mineral element composition of tea plants, increasing the content of zinc, molybdenum, and copper elements in the leaves, and changing the content of selenium and iron elements in the new shoots, with a minor impact on the mineral content of the new shoots; and (4). ZnO NPs improved the community composition of both epiphytic and endophytic microorganisms in the tea plant phyllosphere, suppressing some potentially pathogenic microorganisms and promoting many beneficial microbes that potentially enhance plant photosynthesis and growth to become dominant populations. This research provides new insights into how ZnO NPs can improve the growth of tea plants.

### Supplementary Information

Additional file 1. Steps for RNA extraction in transcriptome sequencing

Additional file 2. Gene expression and metabolite expression in tea plant leaves and shoots under different concentrations of ZnO NPs.

Additional file 3. Analysis of phyllosphere epiphytic microorganisms in tea plants under different concentrations of ZnO NPs.

Additional file 4. Analysis of phyllosphere endophytic microorganisms in tea plants under different concentrations of ZnO NPs.

Additional file 5: Table S1. Primer sequences used for qRT-PCR validation.

Additional file 6: Table S2. Transcriptome raw data filtering, sequencing error rate checking, and GC content distribution checking.

Additional file 7: Table S3. ADONIS analysis of phyllosphere epiphytic microorganisms.

Additional file 8: Table S4. ADONIS analysis of phyllosphere endophytic microorganisms.

Additional file 9: Table S5. Correlation coefficients and significance of differences in the analysis of the correlation between phyllosphere endophytic microorganisms and starch and sucrose metabolism under different concentrations of ZnO NPs.

Additional file 10: Table S6. Types and contents of metabolites in tea plant leaves under different concentrations of ZnO NPs.

Additional file 11: Table S7. Types and contents of metabolites in new shoots of tea plants under different concentrations of ZnO NPs.

Additional file 12: Fig. S1. Number of differentially expressed genes in tea plant leaves (**A**) and new shoots (**B**) under the influence of ZnO NPs.

Additional file 13: Fig. S2. Venn diagrams of differentially expressed genes in tea plant leaves (**A**) and new shoots (**B**) under the influence of ZnO NPs.

Additional file 14: Fig. S3. Cluster heatmaps of differentially expressed genes in tea plant leaves (**A**) and new shoots (**B**) under the influence of ZnO NPs.

Additional file 15: Fig. S4. K-means clustering diagrams of differentially expressed genes in tea plant leaves (**A**) and new shoots (**B**) under the influence of ZnO NPs.

Additional file 16: Fig. S5. Principal component analysis (PCA) of the comprehensive targeted metabolome in tea plant leaves (**A**) and new shoots (**B**) under the influence of ZnO NPs.

Additional file 17: Fig. S6. Volcano plots of differential metabolites in tea plant leaves (**A**, **B**) and new shoots (**C**, **D**) under the influence of ZnO NPs.

Additional file 18: Fig. S7. K-means clustering diagrams of differential metabolites in tea plant leaves (**A**) and new shoots (**B**) under the influence of ZnO NPs.

Additional file 19: Fig. S8. Venn diagrams showing the ASV clustering of epiphytic bacteria (**A**) and fungi (**B**) in the phyllosphere of tea plants under the influence of ZnO NPs.

Additional file 20: Fig. S9. Venn diagrams showing the ASV clustering of endophytic bacteria (**A**) and fungi (**B**) in the phyllosphere of tea plants under the influence of ZnO NPs.

## Data Availability

The raw sequencing data were deposited in NCBI Sequence Read Archive (SRA) under accession number SUB14254443. The original metabolome datasets generated in the current study are available in Table S6 and S7. Additional data will be made available on reasonable request.

## Abbreviations

ZnO NPs: Zinc oxide nanoparticles
CK: Foliar spraying with pure water
CKL: Tea leaves under CK treatment
CKS: Tea shoots under CK treatment
CKEP: Phyllosphere epiphytic microorganisms in tea leaf under CK treatment
CKED: Phyllosphere endophytic microorganisms in tea leaf under CK treatment
T1: Foliar spraying with 50mg L^−^^1^ZnO NPs
T1L: Tea leaves under T1 treatment
T1S: Tea shoots under T1 treatment
T1EP: Phyllosphere epiphytic microorganisms in tea leaf under T1 treatment
T1ED: Phyllosphere endophytic microorganisms in tea leaf under T1 treatment
T2: Foliar spraying with 100mg L^−^^1^ZnO NPs
T2L: Tea leaves under T2 treatment
T2S: Tea shoots under T2 treatment
T2EP: Phyllosphere epiphytic microorganisms in tea leaf under T2 treatment
T2ED: Phyllosphere endophytic microorganisms in tea leaf under T2 treatment
Photo: Net photosynthetic rate
Cond: Stomatal conductance
Ci: Intercellular CO_2_ concentration
Trmmol: Transpiration rate
RubisCO: Ribulose-1,5-bisphosphate carboxylase/oxygenase
FBP: Fructose-1,6-bisphosphatase
PEPC: Phosphoenolpyruvate carboxylase
SPAD: Soil plant analysis development
chl a: Chlorophyll a
chl b: Chlorophyll b
ETR: Relative electron transport rate
Fv/Fm: Maximum photochemical efficiency of photosystem II
qP: Photochemical quenching coefficient
Y(II): Actual photochemical efficiency of photosystem II
WGCNA: Weighted gene co-expression network analysis
DEGs: Differentially expressed genes
O2PLS: Two-way orthogonal partial least squares
PCA: Principal component analysis
ASVs: Amplicon sequence variants
OTUs: Operational taxonomic units
TCA: Tricarboxylic acid
LHCB: Light-harvesting complex II chlorophyll a/b binding protein
