# Supplementary material for: Zno nanoparticles: improving photosynthesis, shoot development, and phyllosphere microbiome composition in tea plants
Source: J Nanobiotechnology. 2024 Jul 2;22:389. doi: 10.1186/s12951-024-02667-2 (PMC11221027; doi:10.1186/s12951-024-02667-2)
Supplement: Supplementary file 1 — Additional file 1. Steps for RNA extraction in transcriptome sequencing [file 12951_2024_2667_MOESM1_ESM.pdf]

Principle of the Experiment:

The CTAB method uses cationic detergent CTAB to lyse cells and precipitate nucleic acids and polysaccharides in low ionic strength solutions. During RNA extraction, CTAB serves as a denaturant in conjunction with β-mercaptoethanol, which denatures proteins and inhibits RNases. After precipitation with ethanol and LiCl, RNA is selectively extracted using chloroform and precipitated with anhydrous ethanol. This method is particularly effective for isolating high-quality RNA from plant tissues that are rich in polysaccharides and polyphenols.

Procedure:

(1) Mix 100 mg of ground plant sample with 1 mL CTAB lysis solution (with β-mercaptoethanol).

(2) Incubate at 800 RPM and 60°C for 10 minutes on a temperature-controlled shaker. Add 200 μL anhydrous ethanol if viscous, mix and let stand for 5 minutes.

(3) Centrifuge at 12,000 g for 5 minutes at 4°C, transferring the supernatant to fresh chloroform after each centrifugation for a total of three chloroform extractions.

(4) Mix the final supernatant with an equal volume of LiCl and leave at -20°C for at least 2 hours.

(5) Following LiCl precipitation, centrifuge, discard the supernatant, wash the pellet with 75% ethanol, and after the final centrifugation, remove any remaining ethanol.

(6) Resuspend the pellet in 30 μL DEPC-treated water, vortex, and store at -80°C.

RNA Quantification and Qualification:

Monitor RNA degradation and contamination on 1% agarose gels. Assess RNA purity with a Nanophotometer (IMPLEN, CA, USA) and measure concentration using the Qubit RNA Assay Kit with a Qubit 2.0 Fluorometer (Life Technologies, CA, USA). Evaluate RNA integrity using the RNA Nano 6000 Assay Kit with the Agilent Bioanalyzer 2100 system (Agilent Technologies, CA, USA).

Library Preparation for Transcriptome Sequencing:

Utilize 1 μg of RNA per sample to prepare sequencing libraries with the NEBNext Ultra RNA Library Prep Kit for Illumina (NEB, USA) according to manufacturer's instructions, adding index

codes to each sample. Purify mRNA using magnetic beads, fragment it with divalent cations, and synthesize first-strand cDNA with M-MuLV Reverse Transcriptase. Perform second-strand synthesis with DNA Polymerase I and RNase H. Blunt ends are created and adenylated, then NEBNext Adapters with hairpin loop structure are ligated for hybridization. Select cDNA fragments of 250-300 bp with the AMPure XP system (Beckman Coulter, USA), and treat with USER Enzyme (NEB, USA) before PCR with Phusion High-Fidelity DNA polymerase. Purify the PCR products with the AMPure XP system and check library quality on the Agilent Bioanalyzer 2100 system.

Clustering and Sequencing:

Cluster index-coded samples using the cBot Cluster Generation System with the TruSeq PE Cluster Kit v3-cBot-HS (Illumina) following manufacturer's instructions. After cluster generation, sequence the libraries on an Illumina platform to obtain 150 bp paired-end reads.
