# Supplementary material for: Zno nanoparticles: improving photosynthesis, shoot development, and phyllosphere microbiome composition in tea plants
Source: J Nanobiotechnology. 2024 Jul 2;22:389. doi: 10.1186/s12951-024-02667-2 (PMC11221027; doi:10.1186/s12951-024-02667-2)
Supplement: Supplementary file 2 — Additional file 2. Gene expression and metabolite expression in tea plant leaves and shoots under different concentrations of ZnO NPs. [file 12951_2024_2667_MOESM2_ESM.pdf]

Gene Expression in Tea Plant Leaves and New Shoots Under the Influence of ZnO NPs

KEGG Enrichment Analysis of Differentially Expressed Genes

To identify the functions or metabolic pathways enriched by differentially expressed genes and to elucidate the differences at the gene function and metabolic pathway levels in tea plant leaves and new shoots after ZnO NPs treatment, we performed Kyoto Encyclopedia of Genes and Genomes (KEGG) enrichment analysis on the differentially expressed genes. The results are shown in Fig. 14. DEGs between T1L and CKL were mainly enriched in Photosynthesis - antenna proteins (ko00196, Q-value=$3.79*10^{-10}$), Biosynthesis of secondary metabolites (ko01110, Q-value=$1.37*10^{-8}$), Circadian rhythm – plant (ko04712, Q-value=$1.45*10^{-8}$), Metabolic pathways (ko01100, Q-value=$2.92*10^{-5}$), alpha-Linolenic acid metabolism (ko00592, Q-value=0.019) and other metabolic pathways (Fig. 14A); DEGs between T2L and CKL were mainly enriched in Circadian rhythm - plant (ko04712, Q-value=$8.24*10^{-12}$), Biosynthesis of secondary metabolites (ko01110, Q-value=$1.22*10^{-7}$), Photosynthesis - antenna proteins (ko00196, Q-value=$2.57*10^{-7}$), Metabolic pathways (ko01100, Q-value=0.005), Nitrogen metabolism (ko00910, Q-value=0.007), Starch and sucrose metabolism (ko00500, Q-value=0.020), Biosynthesis of various plant secondary metabolites (ko00999, Q-value=0.025), Plant hormone signal transduction (ko04075, Q-value=0.027), Cutin, suberine and wax biosynthesis (ko00073, Q-value=0.036) and other metabolic pathways (Fig. 14B). DEGs between T1S and CKS were mainly enriched in Biosynthesis of secondary metabolites (ko01110, Q-value=$1.17*10^{-5}$), Metabolic pathways (ko01100, Q-value=0.013), Ascorbate and aldarate metabolism (ko00053, Q-value=0.031) (Fig. 14C); DEGs between T2S and CKS were mainly enriched in Biosynthesis of secondary metabolites (ko01110,

Q-value=0.032) (Fig. 14D).

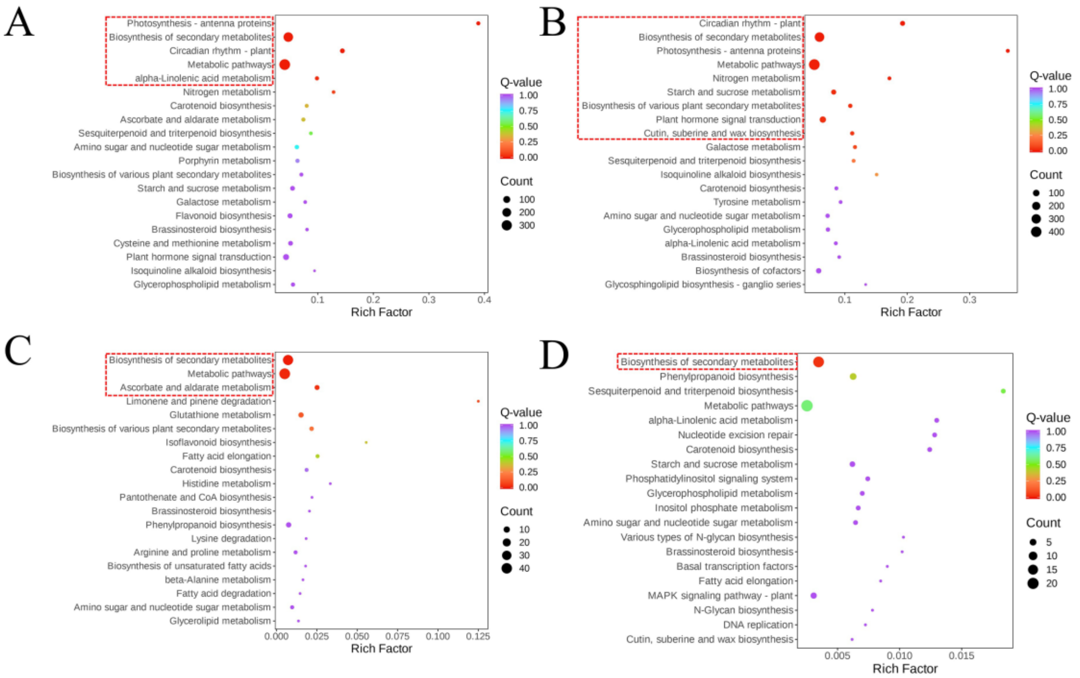

Fig. 14 KEGG enrichment pathways of differentially expressed genes in tea plant leaves (AB) and new shoots (CD) under the influence of ZnO NPs. Enrichment analysis between CKL and T1L (A); between CKL and T2L (B); between CKS and T1S (C); between CKS and T2S (D). Pathways within the red dashed box have a Q-value < 0.05, where Q represents the p-value after correction for multiple hypothesis testing.

GO Enrichment Analysis of Differentially Expressed Genes

To explore the roles of differentially expressed genes in molecular function (MF), biological process (BP), and cellular component (CC) in tea plant leaves and new shoots after ZnO NPs treatment, we conducted a Gene Ontology (GO) enrichment analysis at the second hierarchical level. We selected the top 20 most significantly enriched GO terms for presentation, and the results are shown in Fig. 15. The differential pathways between T1L and CKL were mainly enriched in isoflavonoid

metabolic process, photosynthesis, light harvesting, chlorophyll binding, photosynthesis, light reaction, response to blue light, photosystem I, and photosynthesis, light harvesting in photosystem I, among others (Fig. 15A); between T2L and CKL, the differential pathways were mainly enriched in isoflavonoid biosynthetic process, secondary metabolite biosynthetic process, response to blue light, plant-type secondary cell wall biogenesis, photosynthesis, light reaction, and photosynthesis, light harvesting, among others (Fig. 15B). The differential metabolic pathways between T1S and CKS were mainly enriched in terpenoid biosynthetic process, transferase activity, transferring alkyl or aryl (other than methyl) groups, lactone biosynthetic process, regulation of jasmonic acid mediated signaling pathway, chitinase activity, jasmonic acid metabolic process, phenylpropanoid catabolic process, geraniol 10-hydroxylase activity, and proanthocyanidin biosynthetic process, among others (Fig. 15C); between T2S and CKS, the differential metabolic pathways were mainly enriched in abscisic acid binding, isoprenoid binding, lignin catabolic process, phenylpropanoid catabolic process, hormone binding, phenol-containing compound biosynthetic process, proanthocyanidin biosynthetic process, ether metabolic process, and organic acid binding, among others (Fig. 15D).

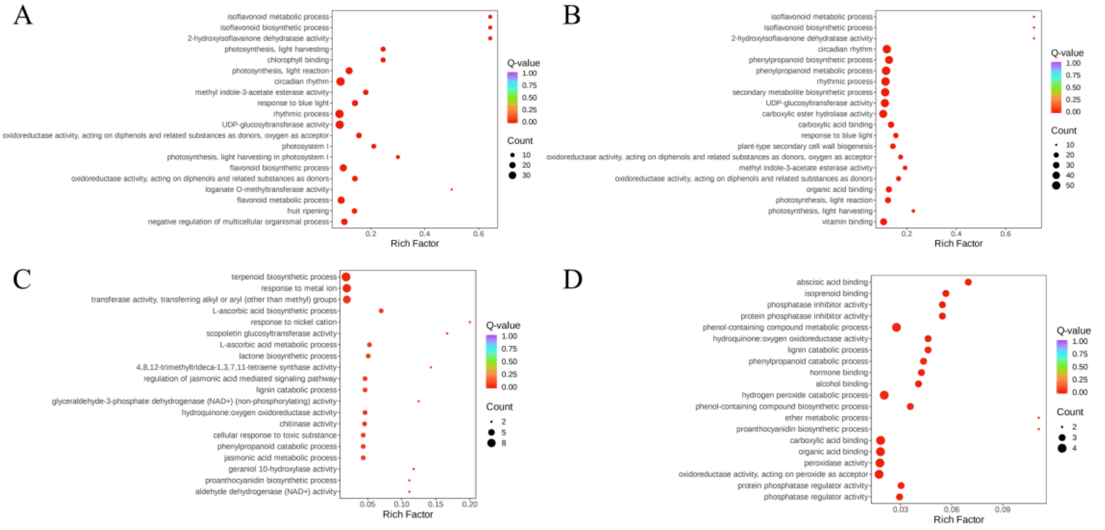

Fig. 15 GO enrichment pathways of differentially expressed genes in tea plant leaves (AB) and new shoots (CD) under the influence of ZnO NPs. Enrichment analysis between CKL and T1L (A); between CKL and T2L (B); between CKS and T1S (C); between CKS and T2S (D). The pathways displayed in the figure all have a Q-value less than 0.05, where Q represents the p-value after correction for multiple hypothesis testing.

qRT-PCR Analysis

To validate the accuracy of the RNASeq data, we randomly selected 20 differentially expressed genes (DEGs), including those related to photosynthesis, sucrose metabolism, and hormones, and verified them using the qRT-PCR method. The expression data of these DEGs from qRT-PCR and RNA-seq are represented by line charts and bar graphs, respectively (Fig. 16). The results showed that the expression trends of the genes were highly correlated with the RNA-Seq results, and their patterns of change were essentially consistent. These findings confirm the reproducibility and reliability of the transcriptome data in this study.

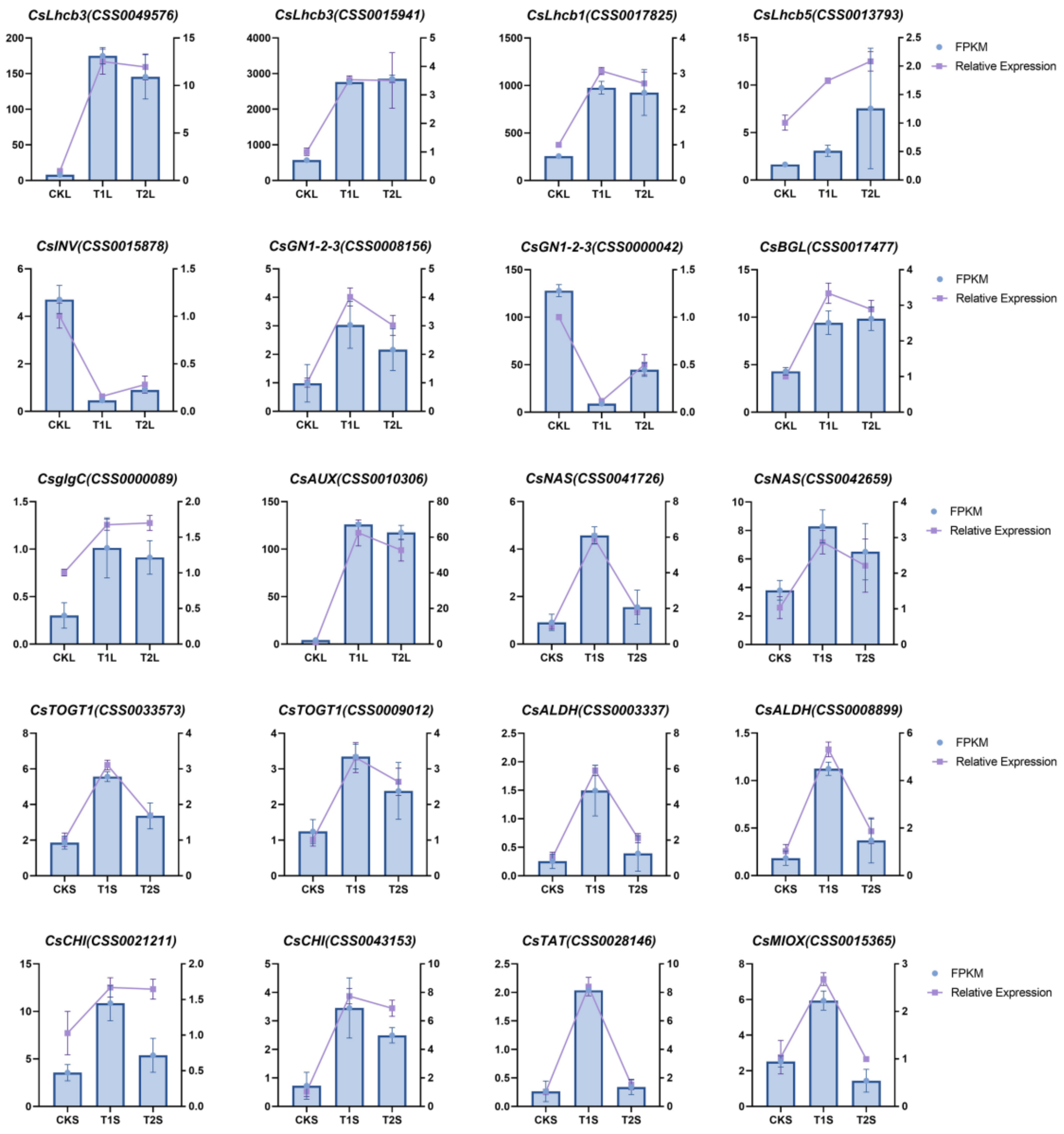

Fig. 16 qRT-PCR validation of 20 DEGs in tea plant leaves and new shoots under the influence of ZnO NPs. The Relative Expression and FPKM of these DEGs are represented by line charts and bar graphs, respectively.

Changes in Metabolite Composition and Content in Tea Plant Leaves and New Shoots Under the Influence of ZnO NPs

Differential Metabolites in Tea Plant Leaves and New Shoots

To cluster and classify samples in the metabolome to reveal similarities and differences between them, we performed a clustering analysis of the samples. As shown in Fig. 17, samples within the same group clustered into the same cluster, while samples from different groups clustered into different clusters, indicating good clustering of the samples. The cluster analysis of T1L and CKL in leaves is shown in Fig. 17A, and T2L and CKL are shown in Fig. 17B. The cluster analysis of T1S and CKS was shown in Fig. 17C, and T2S and CKS were shown in Fig. 17D.

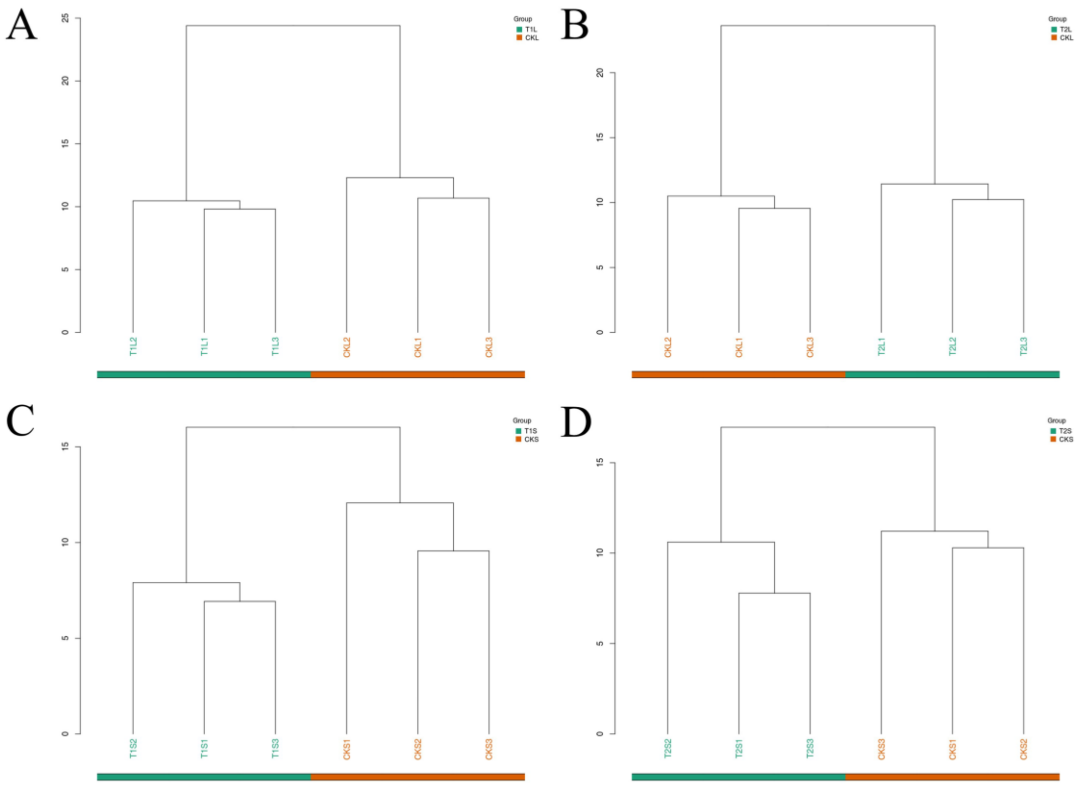

Fig. 17 Clustering analysis of differential metabolites in tea plant leaves (AB) and new shoots (CD) under the influence of ZnO NPs.

To elucidate the differences in types and content of metabolites in tea plant leaves and new shoots after ZnO NPs treatment, we selected metabolites with VIP > 1 and FC (fold change) ⩾2 or ⩽0.5 as differential metabolites. The results are shown in Fig. 18. Fig. 18 only displays the top 20

metabolites with the highest fold changes in each comparison group, while all differential metabolites between groups are listed in Table. S6 (leaves) and Table. S7 (new shoots). The top 20 most significant differential metabolites between T1L and CKL (12 increased and 8 decreased) include Theaflavin-3-gallate, Theaflavin-3'-Gallate, Apigenin-4'-O-(2''-O-p-coumaroyl)-β-D-glucopyranoside, Theaflavin-3,3'-di-O-gallate, Theaflavin, Pinocembrin-7-O-sophoroside, L-Glutamine-O-glycoside, 7-O-(4''-O-glucosyl)coumaroyl-loganic acid, Uridine 5'-diphosphate, Epitheaflagallin-3-O-Gallate, Prodelphinidin A2 3'-gallate, 4-O-p-Coumaroylquinic acid, Benzoylmalic acid, Rhamnazine-5-O-β-D-glucoside, 3-Cinnamoyl-5-Caffeoylquinic acid, N-Acetyl-L-glycine, Jasmonoyl-L-Isoleucine, LysoPC 19:0, Centaurein, 3,4-Di-O-p-Coumaroylquinic acid (Fig. 18A); the top 20 most significant differential metabolites between T2L and CKL (16 increased and 4 decreased) include Theaflavin-3-gallate, Theaflavin-3'-Gallate, 7-O-(4''-O-glucosyl)coumaroyl-loganic acid, Theaflavin-3,3'-di-O-gallate, Apigenin-4'-O-(2''-O-p-coumaroyl)-β-D-glucopyranoside, Theaflavin, Uridine 5'-diphosphate, L-α-Glutamyl-L-Glutamic Acid, Quercetagetin-7-O-glucoside(Quercetagitrin), Gossypetin-3-O-glucoside, Pinocembrin-7-O-sophoroside, 3-O-Galloylepiafzelechin-(4beta->6)-Epigallocatechin-3-O-Gallate, Lys-Asp, Met-Asn, Argininosuccinic acid, 2,6-Dimethyl-6-hydroxy-2,7-octadienyl-β-D-glucoside (Betulalbuside A; Betulabuside A), Benzoylisogomisin O, 6-Deoxyfagomine, Piperidine, Dehydrodiconiferyl alcohol-gamma'-O-glucoside (Fig. 18B). The top 20 most significant differential metabolites between T1S and CKS (10 increased and 10 decreased) include 2-[(1R,2R)-3-oxo-2-[(Z)-5-[3,4,5-trihydroxy-6-(hydroxymethyl)oxan-2-yl]oxypent-2-enyl]cyclopentyl]acetic acid, Dicaffeoylquinic acid-O-glucoside, 4'-O-Methyl-6-hydroxygallocatechin 3-O-(N-Ethylglutamine ester) 3'-Gallate, L-Carnitine, Theaflavin-3-gallate, Theaflavin-3'-Gallate, 3-Ureidopropionic Acid, Bletilols B,

Tricin-7-O-(4'-O-glucoside)-guaiacylglycerol-ether, Pinoresinol-4,4'-O-diglucoside, LysoPC 20:4, 1-O-Feruloylquinic acid, Planteose, 8R-Dihydrodehydrodiconferyl alcohol 4-O-β-D-glucopyranoside, 1,4,8-Trihydroxynaphthalene-1-O-[6'-O-(3",4",5"-trimethylbenzoyl)]glucoside, Luteolin-5,7-di-O-rutinoside, 1-(4-Hydroxybenzoyl)Glucose; 25545-07-7, Phenyl acetate, Glucan, Theasinensin F (Fig. 18C); the top 20 most significant differential metabolites between T2S and CKS (10 increased and 10 decreased) include Dicaffeoylquinic acid-O-glucoside, Pinocembrin-7-O-sophoroside, Adenylocuccinic Acid, L-Carnitine, Theaflavin-3,3'-di-O-gallate, Apigenin-4'-O-(2"-O-p-coumaroyl)-β-D-glucopyranoside, Fagomine, 2,3-O-(S)-hexahydroxydiphenoyl-D-glucose, Sinapoyl malate, Theaflavin, Punigluconin, 1,4,8-Trihydroxynaphthalene-1-O-[6'-O-(3",4",5"-trimethylbenzoyl)]glucoside, 1-Methoxybenzoyl-6-O-Galloyl-D-Glucose, Juglanin, D-Glucosamine, Dehydrodiconiferyl alcohol-gamma'-O-glucoside, Ethyl 3-hydroxybutyrate, Methyl 3-(3-hydroxy-4-methoxyphenyl)propanoate, 3,3'-O-Dimethylellagic Acid-4'-O-arabinoside, Theasinensin F (Fig. 18D).

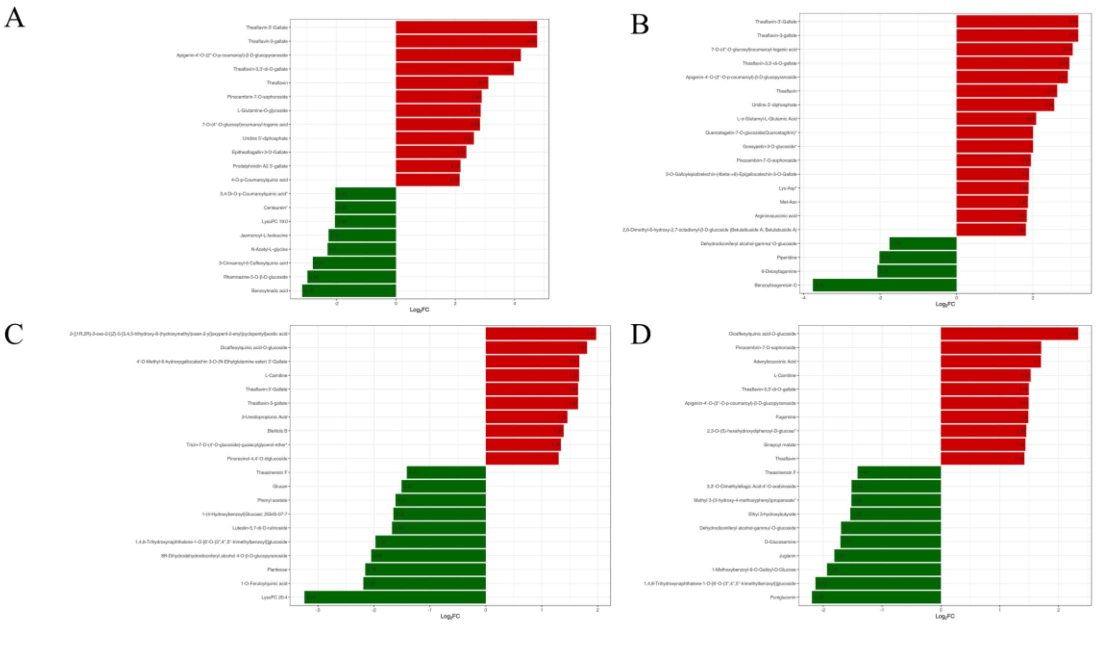

Fig. 18 The top 20 metabolites with the greatest difference in tea plant leaves (AB) and new

shoots (CD) under the influence of ZnO NPs. Differential metabolites between CKL and T1L (A); between CKL and T2L (B); between CKS and T1S (C); between CKS and T2S (D). The colors in the figure indicate an increase (red) or decrease (green) in content.

KEGG Enrichment Analysis of Differential Metabolites

To study the impact of ZnO NPs on the metabolic pathways in tea plants, we performed KEGG enrichment analysis on the differential metabolites in leaves and new shoots treated with ZnO NPs. The results are shown in Fig. 19. The differential metabolic pathways between T1L and CKL were mainly enriched in Starch and sucrose metabolism (ko00500, p=0.002), Linoleic acid metabolism (ko00591, p=0.014), Arginine biosynthesis (ko00220, p=0.02), Anthocyanin biosynthesis (ko00942, p=0.031), Betalain biosynthesis (ko00965, p=0.031), Biotin metabolism (ko00780, p=0.031), Aminoacyl-tRNA biosynthesis (ko00970, p=0.037), Galactose metabolism (ko00052, p=0.037), Caffeine metabolism (ko00232, p=0.0415); the differential metabolic pathways between T2L and CKL were mainly enriched in Starch and sucrose metabolism (ko00500, p=0.002), Carbon fixation in photosynthetic organisms (ko00710, p=0.010), Glucosinolate biosynthesis (ko00966, p=0.010), Tropane, piperidine and pyridine alkaloid biosynthesis (ko00960, p=0.010), Metabolic pathways (ko01100, p=0.012), Biosynthesis of amino acids (ko01230, p=0.013), 2-Oxocarboxylic acid metabolism (ko01210, p=0.014), Aminoacyl-tRNA biosynthesis (ko00970, p=0.0154), Betalain biosynthesis (ko00965, p=0.041), Cyanoamino acid metabolism (ko00460, p=0.043). The differential metabolic pathways between T1S and CKS were mainly enriched in Pantothenate and CoA biosynthesis (ko00770, p=0.019), One carbon pool by folate (ko00670, p=0.021), beta-Alanine metabolism (ko00410, p=0.023); the differential metabolic pathways between

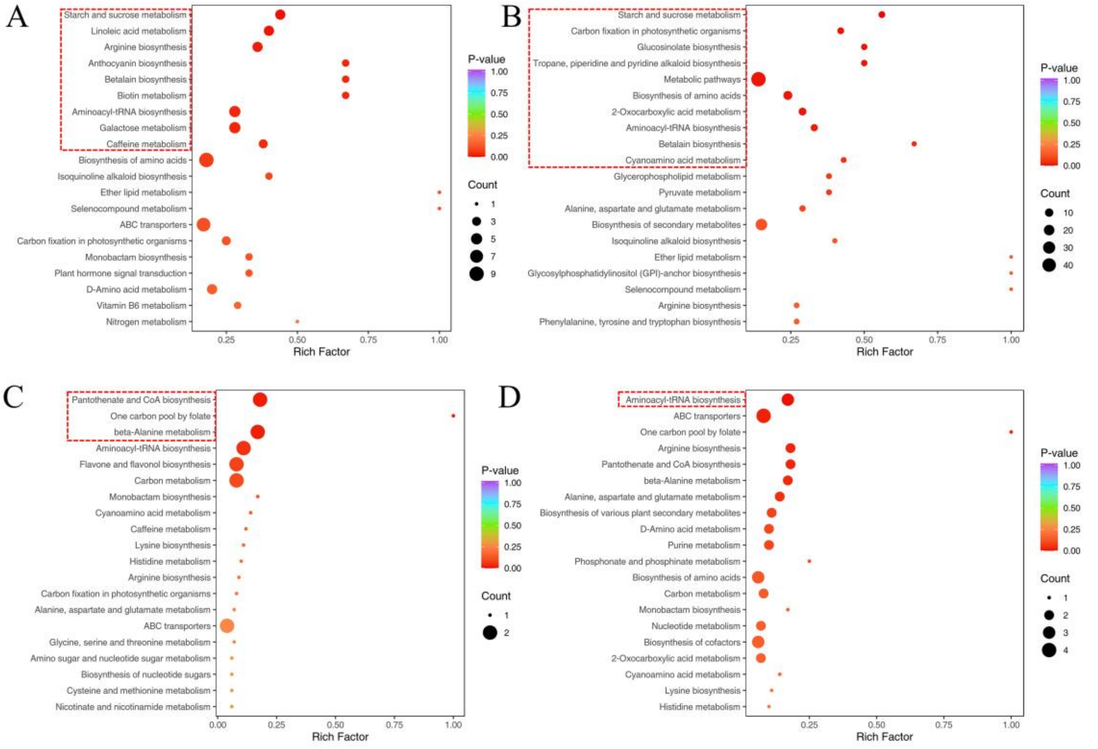

Fig. 19 KEGG enrichment pathways of differential metabolites in tea plant leaves (AB) and new shoots (CD) under the influence of ZnO NPs. Enrichment analysis between CKL and T1L (A); between CKL and T2L (B); between CKS and T1S (C); between CKS and T2S (D). Pathways within the red dashed box have a p-value < 0.05.
