# Supplementary material for: Zno nanoparticles: improving photosynthesis, shoot development, and phyllosphere microbiome composition in tea plants
Source: J Nanobiotechnology. 2024 Jul 2;22:389. doi: 10.1186/s12951-024-02667-2 (PMC11221027; doi:10.1186/s12951-024-02667-2)
Supplement: Supplementary file 3 — Additional file 3. Analysis of phyllosphere epiphytic microorganisms in tea plants under different concentrations of ZnO NPs. [file 12951_2024_2667_MOESM3_ESM.pdf]

Screening and Random Forest Analysis of Differential Epiphytic Microorganisms

To clarify which microbial groups cause the differences in the phyllosphere epiphytic microorganisms of tea plants caused by ZnO NPs, we used LefSe (LDA Effect Size) analysis to screen for differentially abundant microorganisms, i.e., to find statistically significant biomarkers. There were 14 important biomarkers affecting the bacterial community structure differences between CKEP and T1EP, with CKEP dominated by *Marinococcus sp JL786*, *Marinococcus*, *Bacilli*, unidentified *Bacillales*, *Bacillales*, unidentified *Myxococcota*, and *metagenome*, while T1EP was dominated by *Halomonadaceae*, unidentified *Actinobacteria*, *Actinobacteria*, *Micrococcales*, *Microbacterium*, *Microbacteriaceae*, and *Micrococcaceae* (Fig. 20A). There were 18 important biomarkers affecting the bacterial community structure differences between CKEP and T2EP, with CKEP dominated by *Marinococcus sp JL786*, *Marinococcus*, *Bacilli*, unidentified *Bacillales*, *Bacillales*, *Firmicutes*, *Xanthobacteraceae*, unidentified *Myxococcota*, and unidentified *Myxococcota*, while T2EP was dominated by *Microbacterium*, unidentified *Actinobacteria*, *Micrococcales*, *Microbacteriaceae*, *Actinobacteria*, *Candidatus Portiera aleyrodidarum*, *Patulibacter minatonensis*, *Micrococcaceae*, and unidentified *Halomonadaceae* (Fig. 20B). There were 29 important biomarkers affecting the fungal community structure differences between CKEP and T1EP, with CKEP dominated by *Hypocreales*, *Acremonium alternatum*, *Sterigmatomyces halophilus*, *Glomerellaceae*, *Acremonium*, *Sterigmatomyces*, *Agaricostilbaceae*, *Sordariomycetes order Incertae sedis*, *Hypocreales family Incertae sedis*, *Agaricostilbales*, *Agaricostilbomycetes*, *Colletotrichum*, *Trichocomaceae*, *Penicillium*, *Eurotiales*, and *Eurotiomycetes*, while T1EP was dominated by *Pleosporales*, *Schizophyllum commune*, *Agaricales*, *Schizophyllum*, *Lophiostoma*,

Pleosporales family Incertae sedis, *Schizophyllaceae*, *Lophiostomataceae*, *Filobasidiales*, *Monographella cucumerina*, *Zygomycota*, *Polyporales*, and *Monographella* (Fig. 20C). There were 17 important biomarkers affecting the fungal community structure differences between CKEP and T2EP, with CKEP dominated by *Sterigmatomyces halophilus*, *Acremonium alternatum*, *Hypocreales*, *Sterigmatomyces*, *Agaricostilbaceae*, *Acremonium*, *Agaricostilbales*, *Hypocreales family Incertae sedis*, *Agaricostilbomycetes*, *Trichocomaceae*, *Penicillium*, *Eurotiomycetes*, *Eurotiales*, *Hansfordia pulvinata*, and *Pleosporaceae*, while T2EP was dominated by *Cystofilobasidiales* and *Cystofilobasidiaceae* (Fig. 20D). The results indicate that ZnO NPs have a significant impact on both phyllosphere epiphytic bacteria and fungi in tea plants.

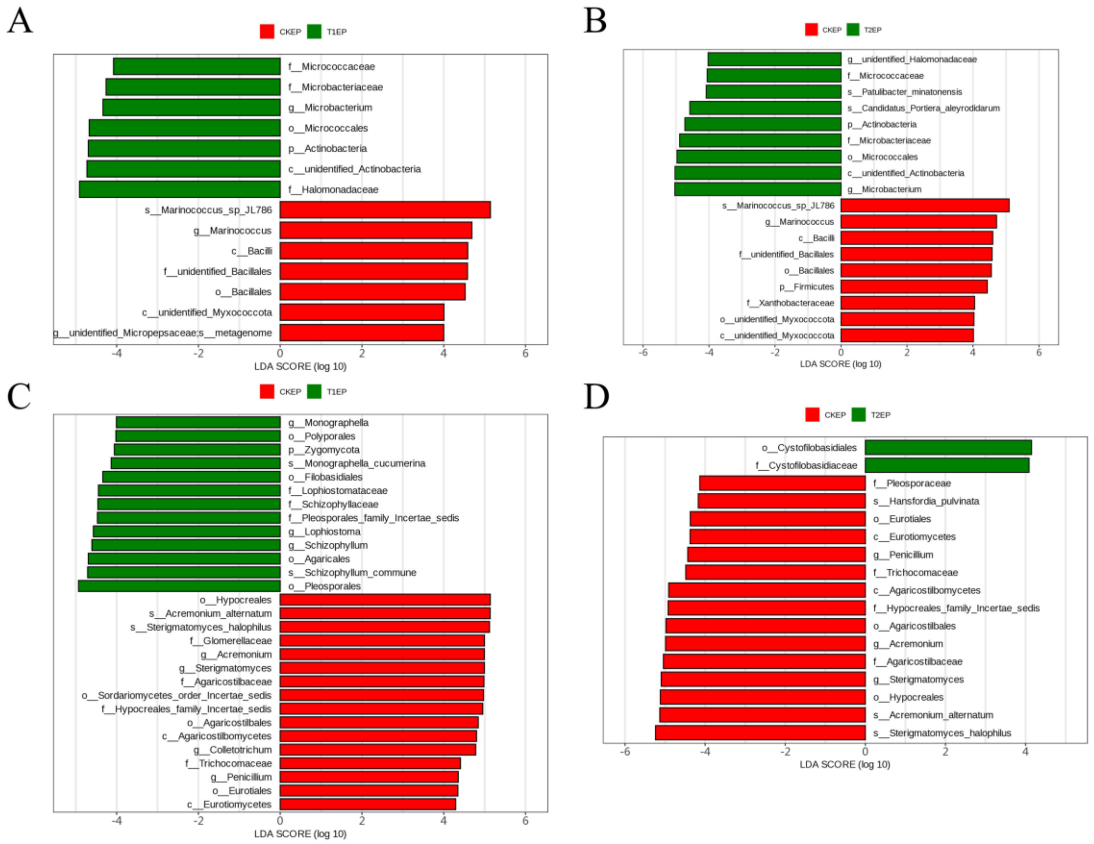

Fig. 20 Significant species with differential bacterial abundance between CKEP and T1EP under the influence of ZnO NPs (A); significant species with differential bacterial abundance between CKEP and T2EP (B); significant species with differential fungal abundance between

CKEP and T1EP (C); significant species with differential fungal abundance between CKEP and T2EP (D). The LDA value distribution bar chart shows species with an LDA Score greater than the set value (default is 4), i.e., biomarkers with statistical differences between groups, and the length of the bar chart represents the impact size of the differential species.

To identify key species of phyllosphere epiphytic microorganisms in tea plants under the influence of ZnO NPs, and thus understand the diversity and function of the microbial community, we conducted a random forest analysis (Fig. 21). MeanDecreaseGini calculates the impact of each variable on the heterogeneity of observations at each node of the classification tree through the Gini index, thereby comparing the importance of variables. The larger the value, the more important the variable is. *Marmoricola* (Fig. 21A) and *Penicillium* (Fig. 21C) were able to explain the largest changes in the epiphytic bacterial and fungal communities, respectively, at 50mg L^{-1} ZnO NPs; *Microbacterium* (Fig. 21B) and *Pseudeurotium* (Fig. 21D) were able to explain the largest changes in the epiphytic bacterial and fungal communities, respectively, at 100mg L^{-1} ZnO NPs. The Area Under Curve (AUC) was 1 for both, indicating good accuracy and discrimination ability.

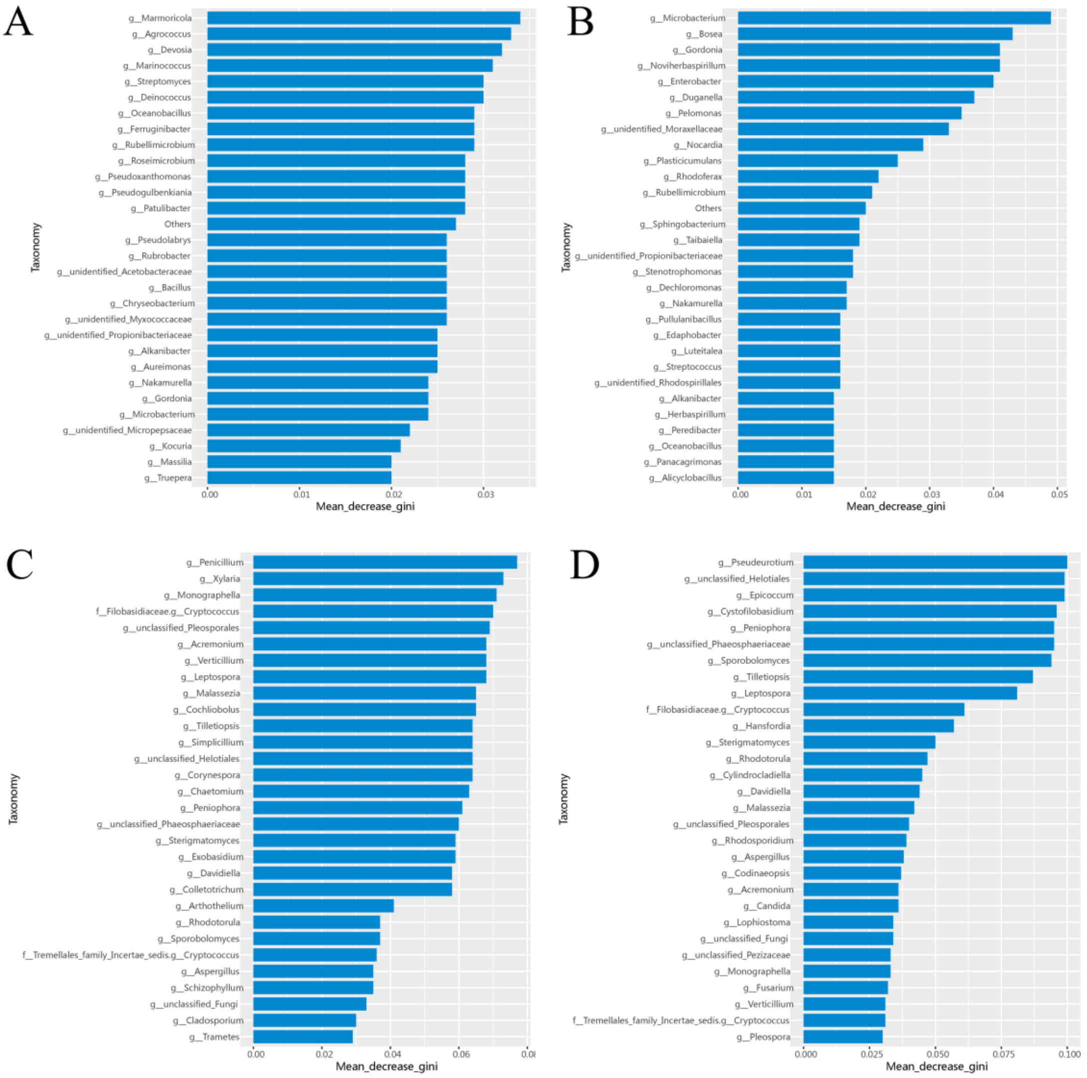

Fig. 21 Key epiphytic microbial species (at the genus level) in the phyllosphere of tea plants under the influence of ZnO NPs. Key epiphytic bacteria in the tea plant phyllosphere under the influence of T1 (A); key epiphytic bacteria in the tea plant phyllosphere under the influence of T2 (B); key epiphytic fungi in the tea plant phyllosphere under the influence of T1 (C); key epiphytic fungi in the tea plant phyllosphere under the influence of T2 (D).
