# Supplementary material for: Zno nanoparticles: improving photosynthesis, shoot development, and phyllosphere microbiome composition in tea plants
Source: J Nanobiotechnology. 2024 Jul 2;22:389. doi: 10.1186/s12951-024-02667-2 (PMC11221027; doi:10.1186/s12951-024-02667-2)
Supplement: Supplementary file 4 — Additional file 4. Analysis of phyllosphere endophytic microorganisms in tea plants under different concentrations of ZnO NPs. [file 12951_2024_2667_MOESM4_ESM.pdf]

Analysis of the Structure of Tea Plant Phyllosphere Endophytic Microorganisms Under the Influence of ZnO NPs

Screening and Random Forest Analysis of Differential Endophytic Microorganisms

To clarify which microbial groups are responsible for the differences in the phyllosphere endophytic microorganisms of tea plants caused by ZnO NPs, we used LefSe analysis to screen for differentially abundant microorganisms. There were 3 important biomarkers affecting the bacterial community structure differences between CKED and T1ED, with CKED dominated by *Oxalobacteraceae*, and T1ED dominated by *Sphingomonadaceae* (Fig. 22A). There were 5 important biomarkers affecting the bacterial community structure differences between CKED and T2ED, with CKED dominated by *Oxalobacteraceae*, and T2ED dominated by *Agrobacterium radiobacter*, *Microbacterium*, *Microbacteriaceae*, and *Sphingomonas* (Fig. 22B). There were 37 important biomarkers affecting the fungal community structure differences between CKED and T1ED, with CKED dominated by *Lophiostoma*, *Lophiostomataceae*, *Pleosporales, Uwebraunia musae, Ascomycota, Aureobasidium pullulans, Sarocladium strictum, Acremonium alternatum, Taphrinaceae sp,* and *Monographella cucumerina*, while T1ED was dominated by *Sordariomycetes, Hypocreales, fungal sp P1S11,* and *Pyrenochaeta* (Fig. 22C). There were 39 important biomarkers affecting the fungal community structure differences between CKED and T2ED, with CKED dominated by *Lophiostomataceae Lophiostoma Ascomycota Dothideomycetes Uwebraunia musae Uwebraunia Sarocladium strictum Aureobasidium pullulans Hypocreales family Incertae sedis Acremonium alternatum Monographella cucumerina Sarocladium Taphrinaceae sp* and *Microcyclospora*, while T2ED was dominated by *Pyrenochaeta inflorescentiae Pyrenochaeta Pleosporales family Incertae sedis fungal*

sp P1S11 unclassified Fungi unclassified Fungi unclassified Fungi unclassified Fungi unclassified Fungi Trichocomaceae Nectriaceae Eurotiomycetes Malassezia Aspergillus subversicolor Malasseziales family Incertae sedis Boletales Agaricomycetes Eurotiales Fusarium Boletus Basidiomycota class Incertae sedis Aspergillus Boletaceae and *Malassezia restricta* (Fig. 22D). The results indicate that ZnO NPs have a significant impact on both phyllosphere endophytic bacteria and fungi in tea plants.

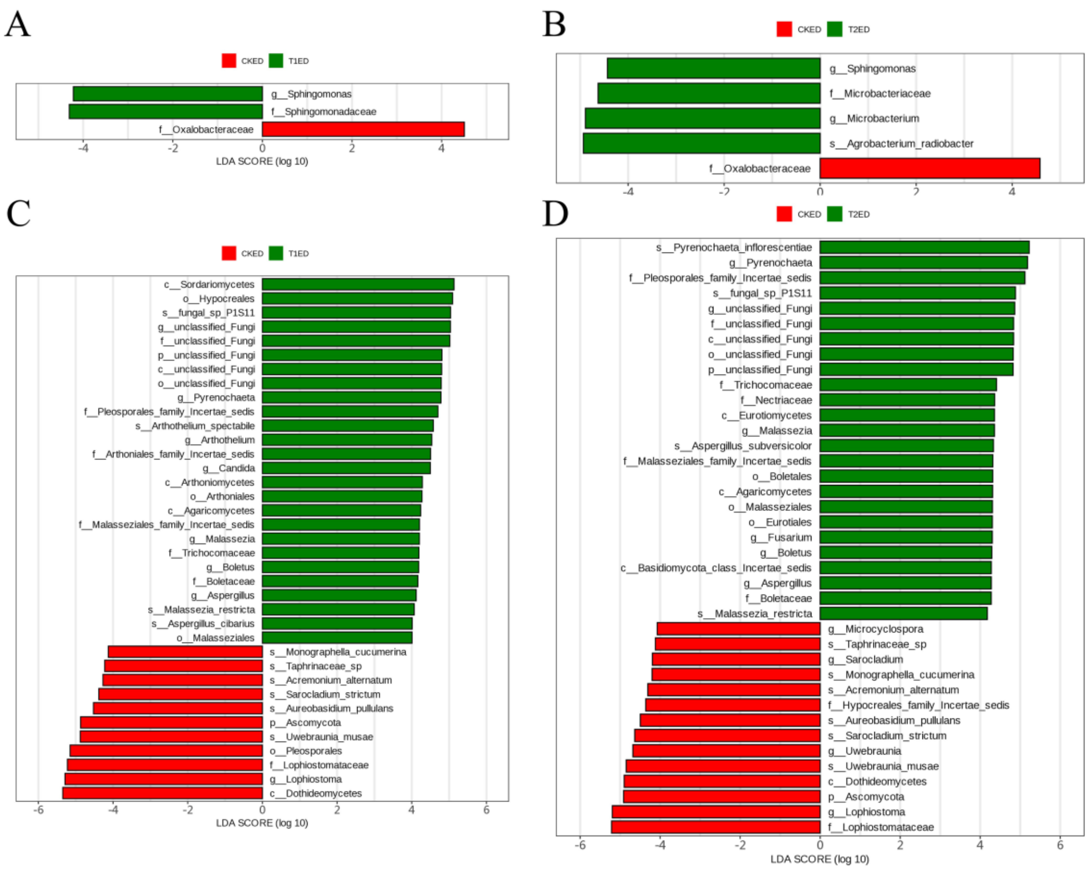

Fig. 22 Significant species with differential bacterial abundance between CKED and T1ED under the influence of ZnO NPs (A); significant species with differential bacterial abundance between CKED and T2ED (B); significant species with differential fungal abundance between CKED and T1ED (C); significant species with differential fungal abundance between CKED and T2ED (D). The LDA value distribution bar chart shows species with an LDA Score greater

than the set value (default is 4), i.e., biomarkers with statistical differences between groups, and the length of the bar chart represents the impact size of the differential species.

To identify key species of phyllosphere endophytic microorganisms in tea plants under the influence of ZnO NPs, similar to epiphytic microorganisms, we conducted a random forest analysis (Fig. 23). *Sphingomonas* (Fig. 23A) and *Colletotrichum* (Fig. 23C) could explain the largest changes in the endophytic bacterial and fungal communities, respectively, at 50mg L^{-1} ZnO NPs; *Microbacterium* (Fig. 21B) and *Nadsonia* (Fig. 23D) could explain the largest changes in the bacterial and endophytic fungal communities, respectively, at 100mg L^{-1} ZnO NPs. The AUC was 1 for both, indicating good accuracy and discrimination ability.

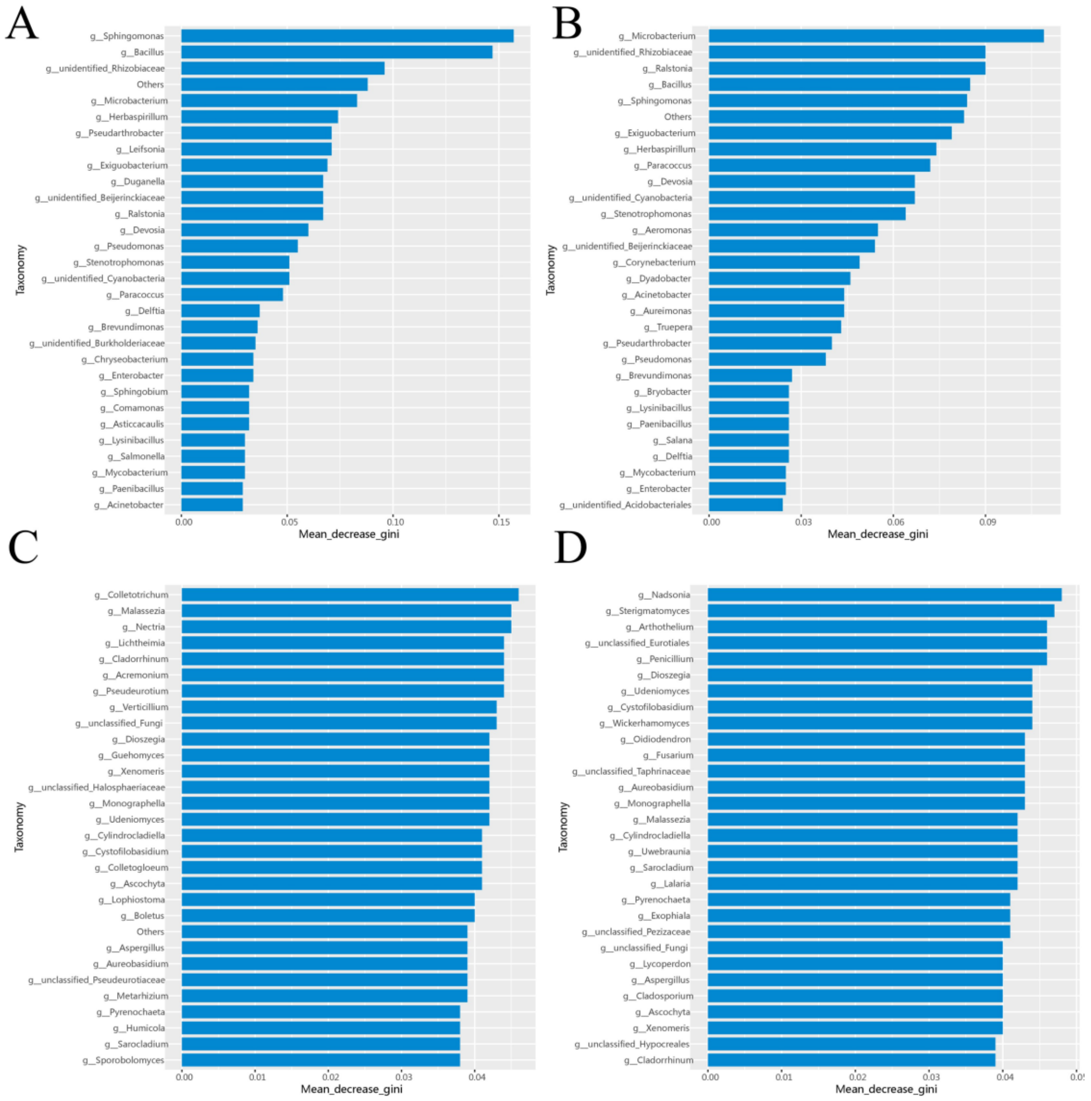

Fig. 23 Key endophytic microbial species (at the genus level) in the phyllosphere of tea plants under the influence of ZnO NPs. Key endophytic bacteria in the tea plant phyllosphere under the influence of T1 (A); key endophytic bacteria in the tea plant phyllosphere under the influence of T2 (B); key endophytic fungi in the tea plant phyllosphere under the influence of T1 (C); key endophytic fungi in the tea plant phyllosphere under the influence of T2 (D).
