# Supplementary material for: Zno nanoparticles: improving photosynthesis, shoot development, and phyllosphere microbiome composition in tea plants
Source: J Nanobiotechnology. 2024 Jul 2;22:389. doi: 10.1186/s12951-024-02667-2 (PMC11221027; doi:10.1186/s12951-024-02667-2)
Supplement: Supplementary file 7 — Additional file 7: Table S3. ADONIS analysis of phyllosphere epiphytic microorganisms. [file 12951_2024_2667_MOESM7_ESM.pdf]

Table. S3 ADONIS analysis of phyllosphere epiphytic microorganisms.

Sample	SumsOfSqs		F.Model		R^2		P	
	Bacteria	Fungi	Bacteria	Fungi	Bacteria	Fungi	Bacteria	Fungi
CK vs T1	0.349	0.581	1.713	2.72	0.3	0.405	0.1	0.1
CK vs T2	0.208	0.687	1.335	2.661	0.25	0.4	0.2	0.01
T1 vs T2	0.27	0.402	1.655	1.152	0.293	0.224	0.1	0.4
