# Supplementary material for: Zno nanoparticles: improving photosynthesis, shoot development, and phyllosphere microbiome composition in tea plants
Source: J Nanobiotechnology. 2024 Jul 2;22:389. doi: 10.1186/s12951-024-02667-2 (PMC11221027; doi:10.1186/s12951-024-02667-2)
Supplement: Supplementary file 8 — Additional file 8: Table S4. ADONIS analysis of phyllosphere endophytic microorganisms. [file 12951_2024_2667_MOESM8_ESM.pdf]

Table. S4 ADONIS analysis of phyllosphere endophytic microorganisms.

Sample	SumsOfSqs		F.Model		R^2		P	
	Bacteria	Fungi	Bacteria	Fungi	Bacteria	Fungi	Bacteria	Fungi
CK vs T1	0.006	0.804	0.303	9.144	0.3	0.696	0.7	0.1
CK vs T2	0.007	0.717	1.602	8.333	0.286	0.676	0.4	0.1
T1 vs T2	0.004	0.424	1.199	18.168	0.231	0.82	0.5	0.1
