# Supplementary figures and images for: Zno nanoparticles: improving photosynthesis, shoot development, and phyllosphere microbiome composition in tea plants
Source: J Nanobiotechnology. 2024 Jul 2;22:389. doi: 10.1186/s12951-024-02667-2 (PMC11221027; doi:10.1186/s12951-024-02667-2)

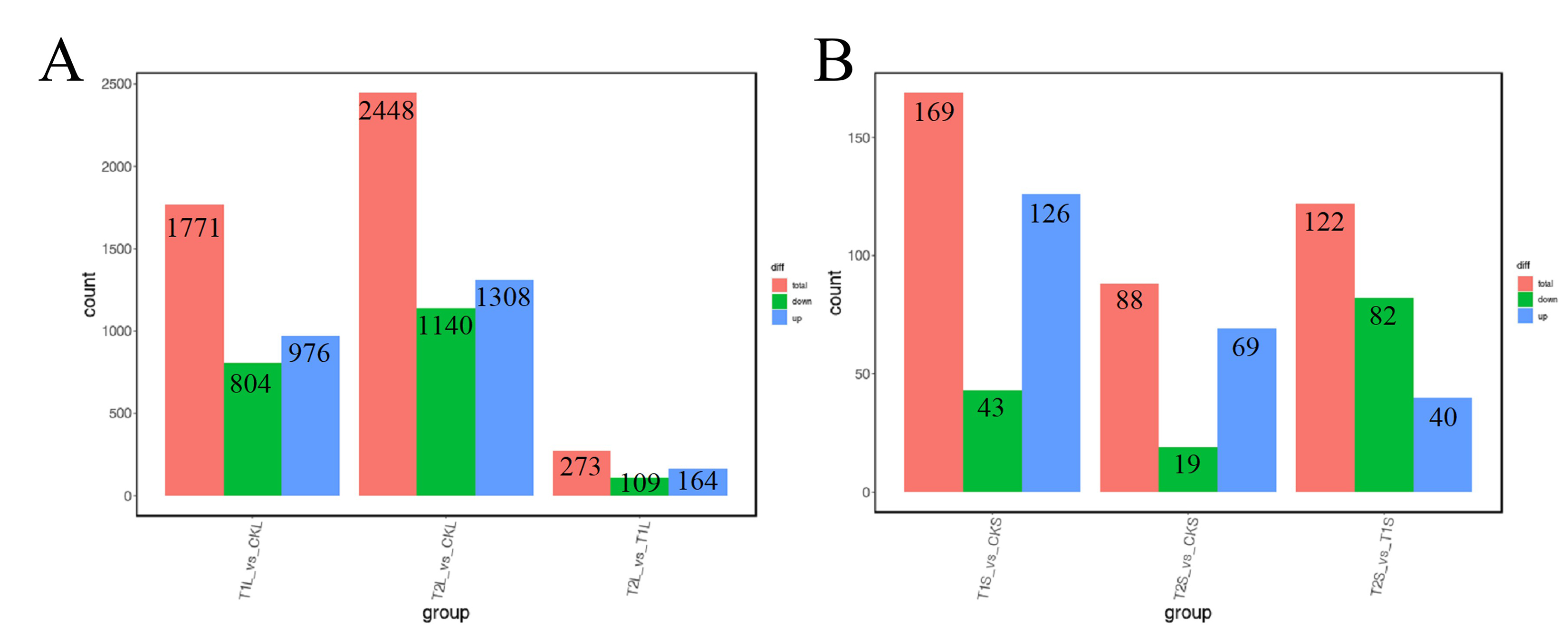

Supplement: Supplementary file 12 — Additional file 12: Fig. S1. Number of differentially expressed genes in tea plant leaves (A) and new shoots (B) under the influence of ZnO NPs. [file 12951_2024_2667_MOESM12_ESM.jpg]

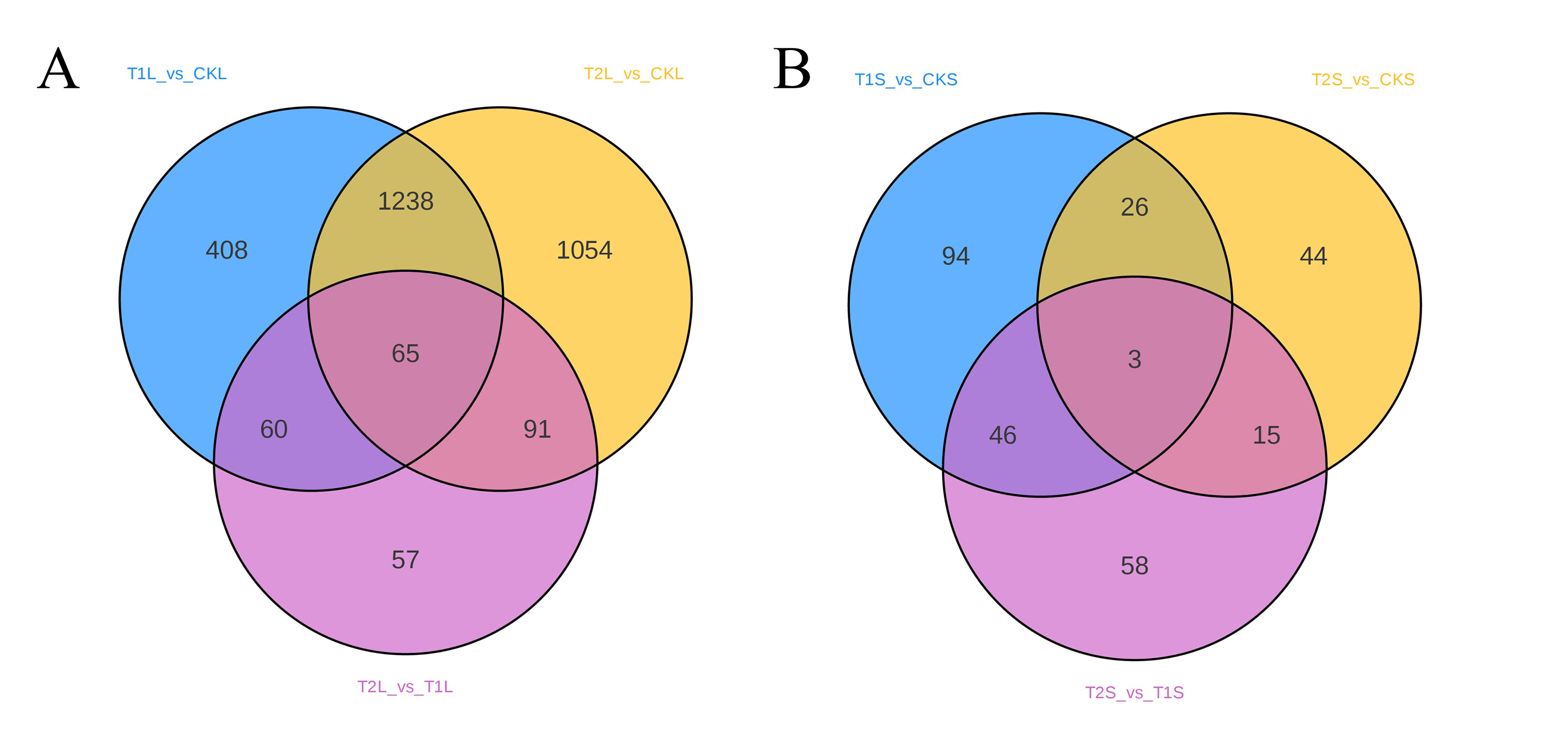

Supplement: Supplementary file 13 — Additional file 13: Fig. S2. Venn diagrams of differentially expressed genes in tea plant leaves (A) and new shoots (B) under the influence of ZnO NPs. [file 12951_2024_2667_MOESM13_ESM.jpg]

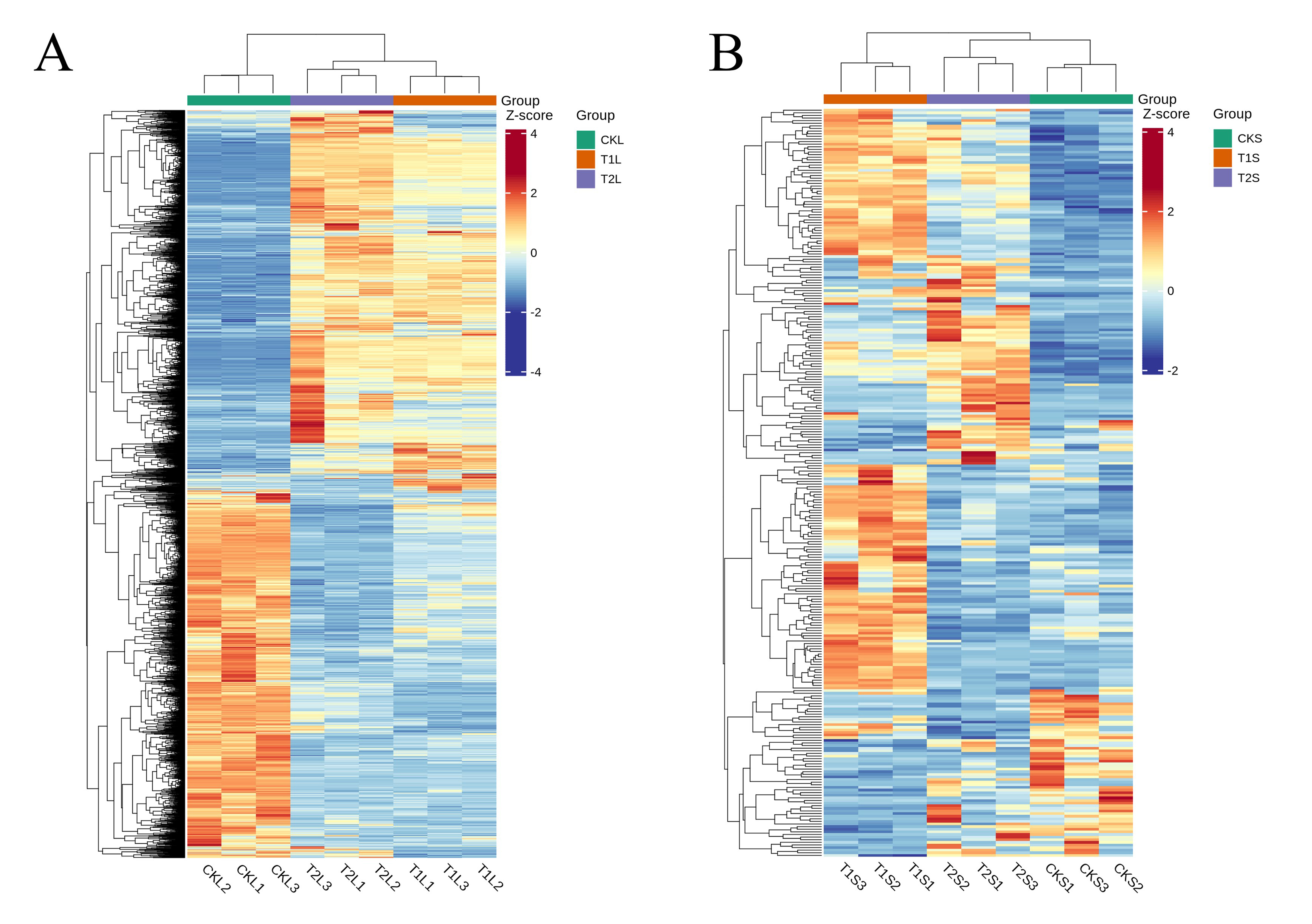

Supplement: Supplementary file 14 — Additional file 14: Fig. S3. Cluster heatmaps of differentially expressed genes in tea plant leaves (A) and new shoots (B) under the influence of ZnO NPs. [file 12951_2024_2667_MOESM14_ESM.jpg]

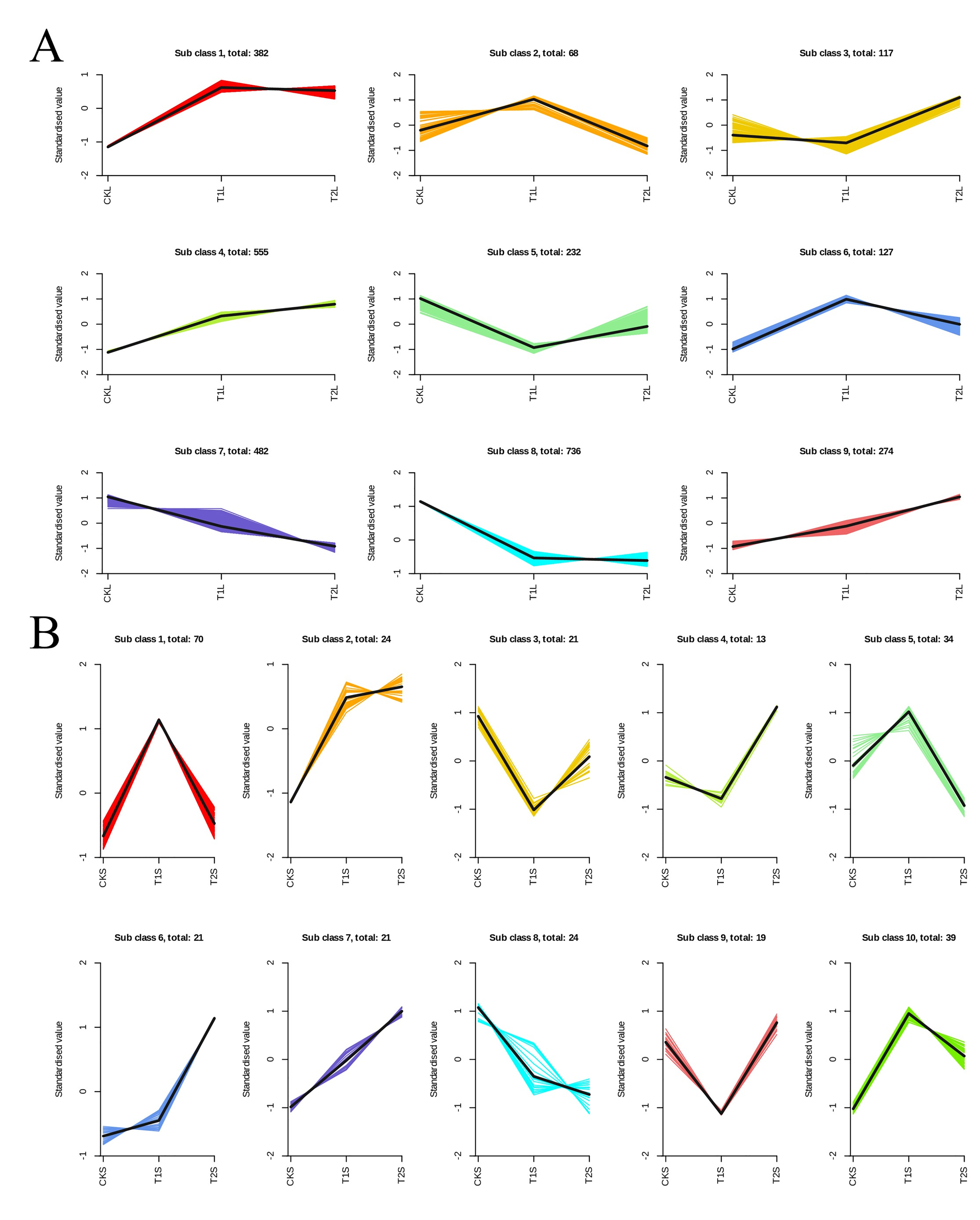

Supplement: Supplementary file 15 — Additional file 15: Fig. S4. K-means clustering diagrams of differentially expressed genes in tea plant leaves (A) and new shoots (B) under the influence of ZnO NPs. [file 12951_2024_2667_MOESM15_ESM.tif]

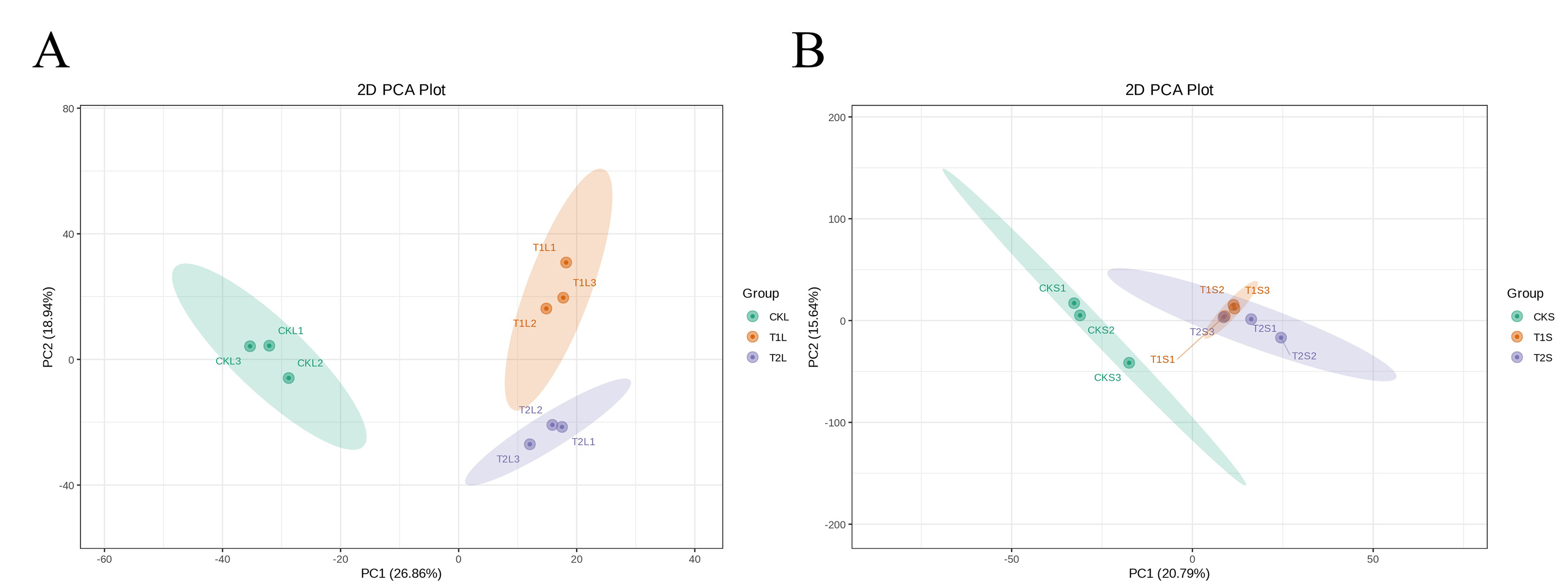

Supplement: Supplementary file 16 — Additional file 16: Fig. S5. Principal component analysis (PCA) of the comprehensive targeted metabolome in tea plant leaves (A) and new shoots (B) under the influence of ZnO NPs. [file 12951_2024_2667_MOESM16_ESM.jpg]

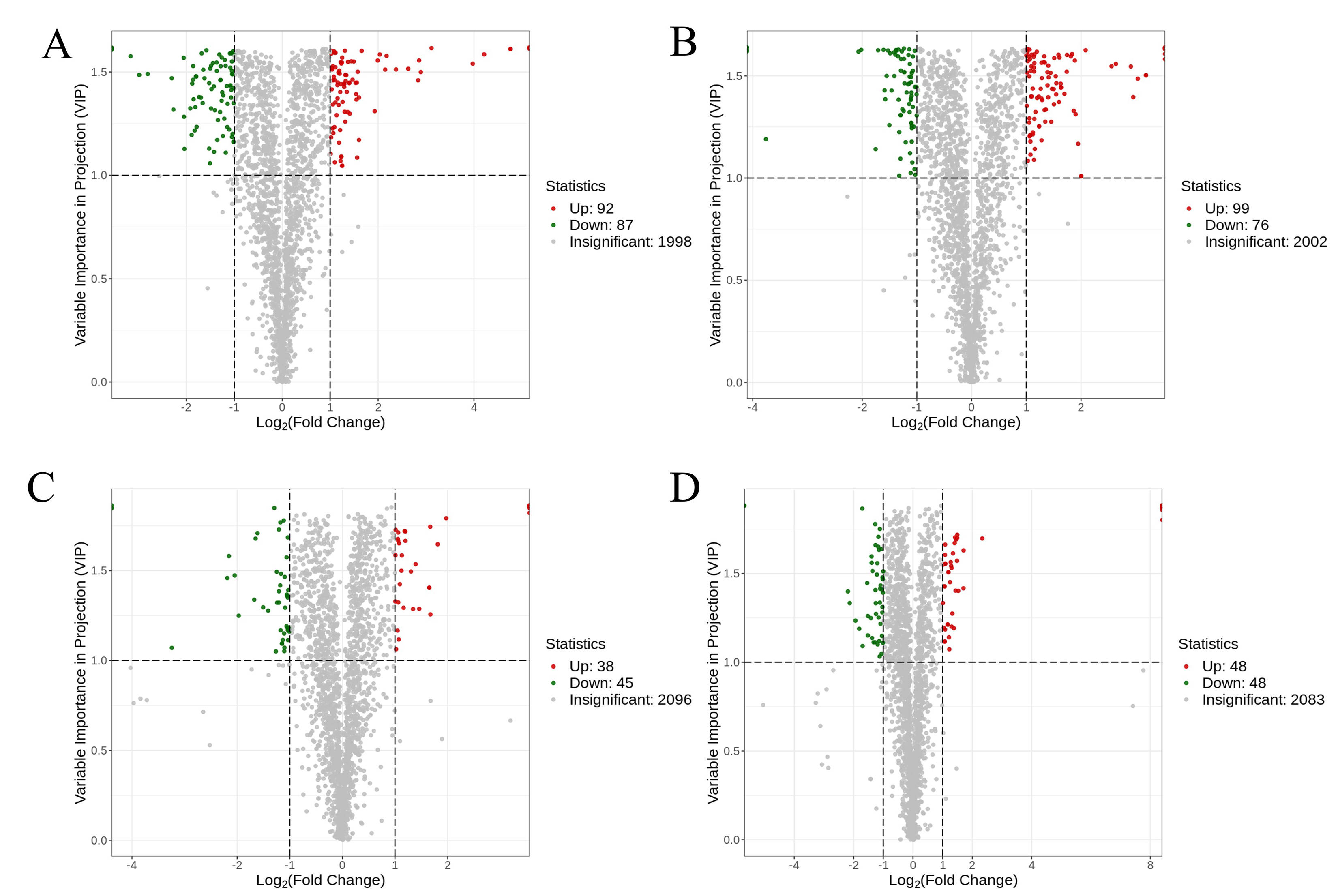

Supplement: Supplementary file 17 — Additional file 17: Fig. S6. Volcano plots of differential metabolites in tea plant leaves (A, B) and new shoots (C, D) under the influence of ZnO NPs. [file 12951_2024_2667_MOESM17_ESM.jpg]

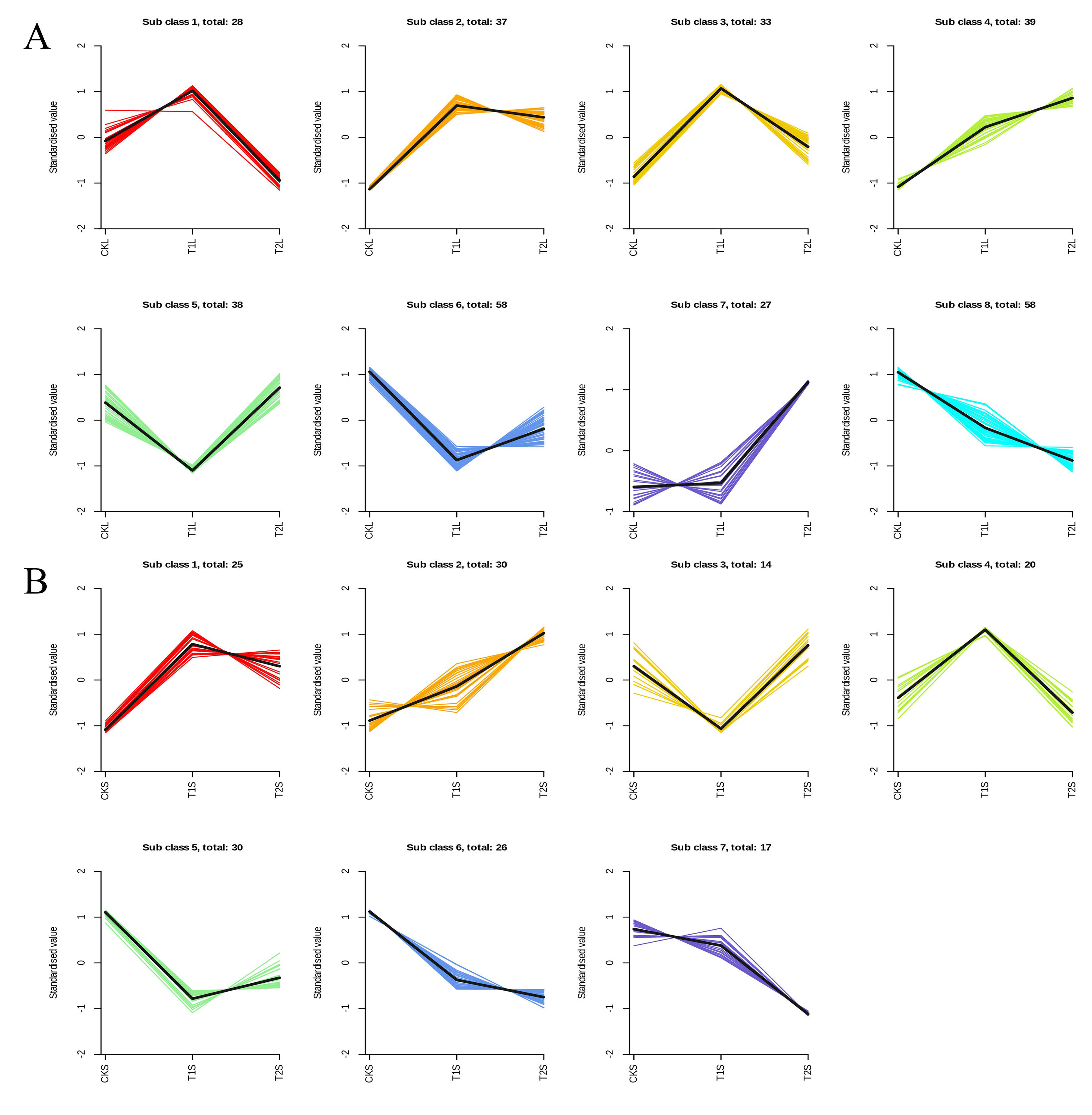

Supplement: Supplementary file 18 — Additional file 18: Fig. S7. K-means clustering diagrams of differential metabolites in tea plant leaves (A) and new shoots (B) under the influence of ZnO NPs. [file 12951_2024_2667_MOESM18_ESM.jpg]

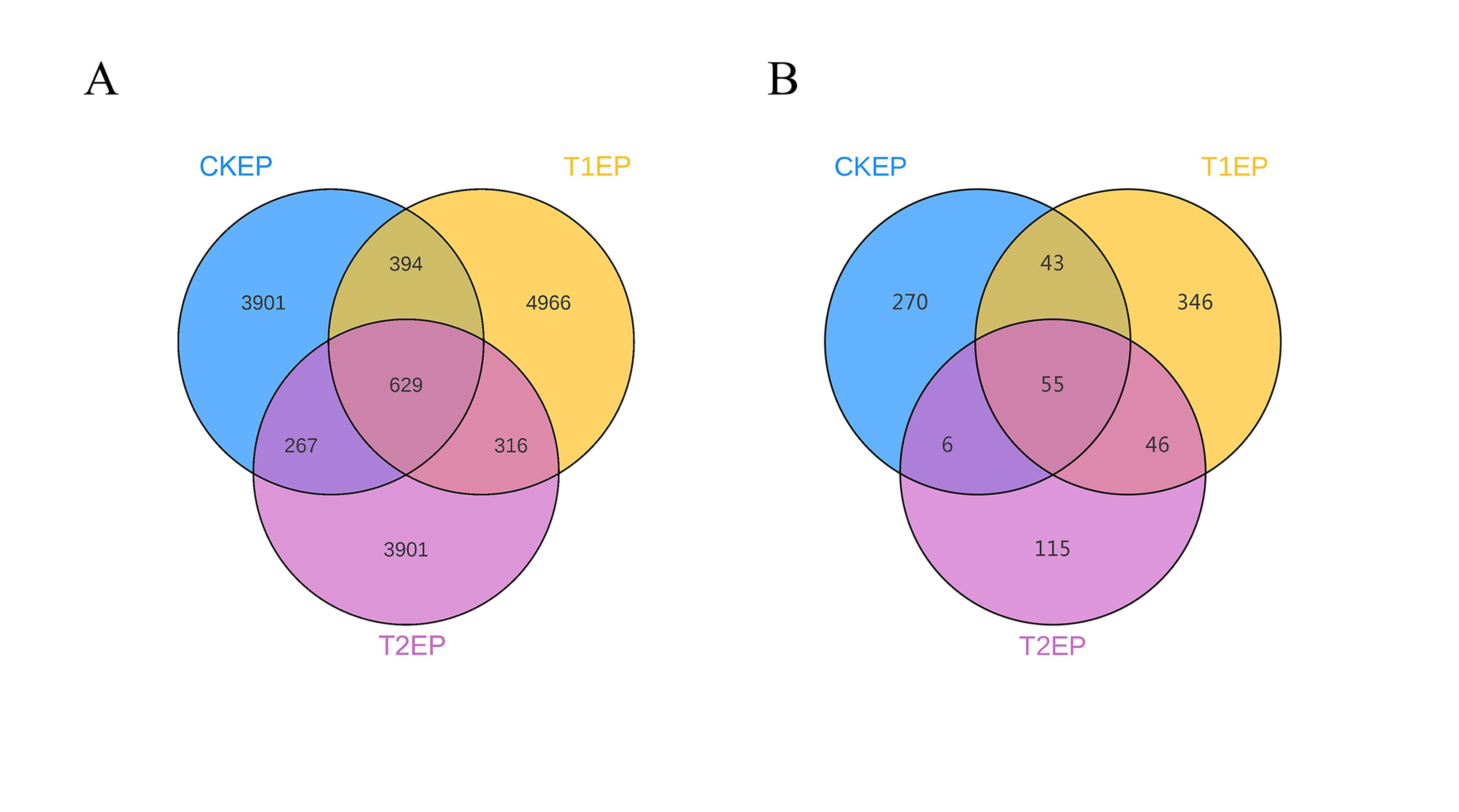

Supplement: Supplementary file 19 — Additional file 19: Fig. S8. Venn diagrams showing the ASV clustering of epiphytic bacteria (A) and fungi (B) in the phyllosphere of tea plants under the influence of ZnO NPs. [file 12951_2024_2667_MOESM19_ESM.jpg]

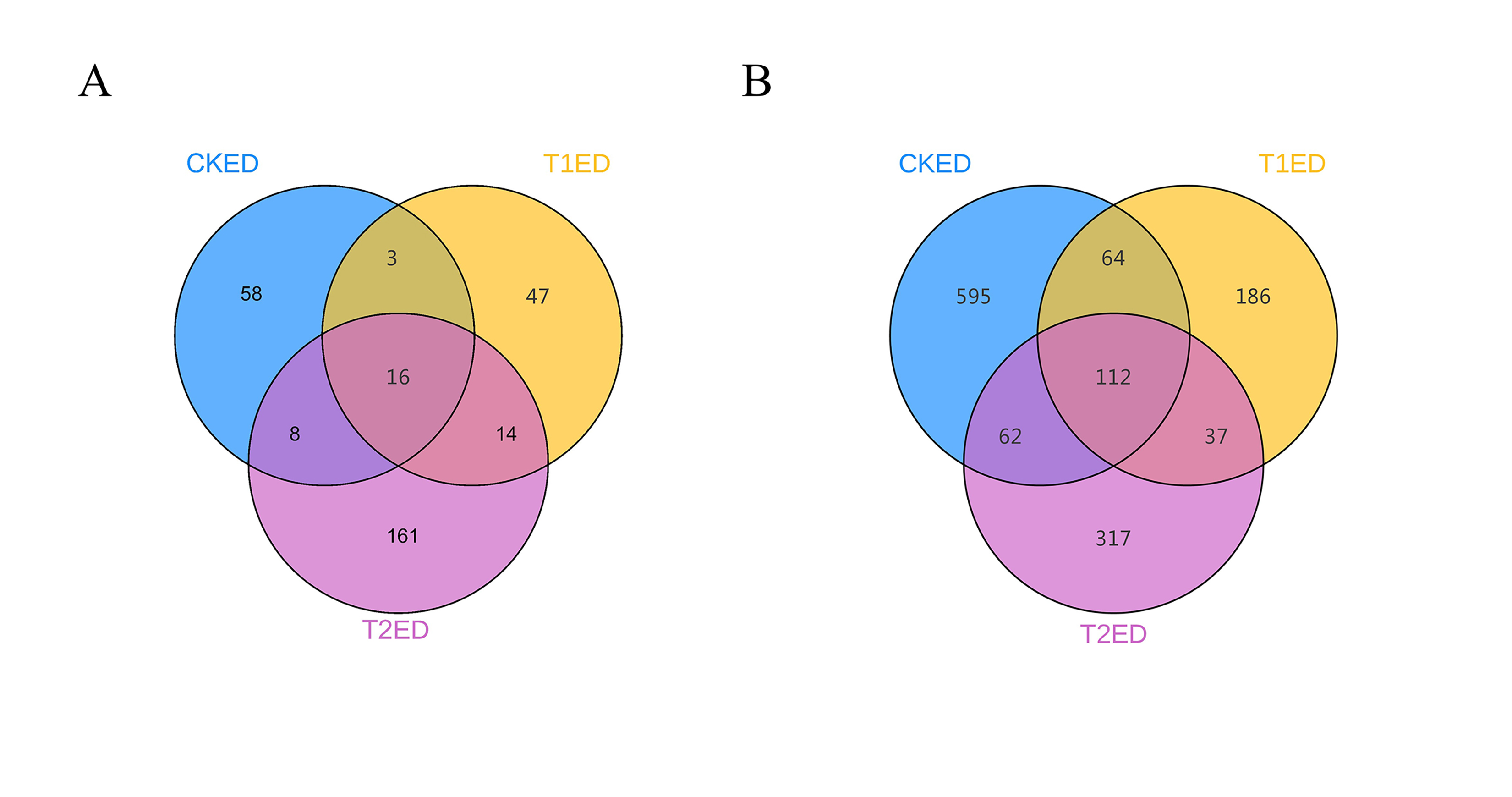

Supplement: Supplementary file 20 — Additional file 20: Fig. S9. Venn diagrams showing the ASV clustering of endophytic bacteria (A) and fungi (B) in the phyllosphere of tea plants under the influence of ZnO NPs. [file 12951_2024_2667_MOESM20_ESM.jpg]
